# Androgen Therapy in 70 Cases of Advanced Mammary Carcinoma

**DOI:** 10.1038/bjc.1950.3

**Published:** 1950-03

**Authors:** D. A. G. Galton

## Abstract

**Images:**


### ANDROGEN THERAPY IN 70 CASES OF ADVANCED MAMMARY CARCINOMA.

D. A. G. GALTON.

*From the Royal Cancer Hospital, Fulham Road, London, S.W.3.*

Received for publication November 2, 1949.

LATHROP AND LOEB (1916) showed that the incidence of mammary cancers in female mice could be reduced, and their time of appearance delayed, by castration at an early age. This was the beginning of intensive experimental study of the relation between new growth and hormonal environment, but clinicians had long been aware of a connection between ovarian or testicular activity and mammary and prostatic tumours. White (1893) described the regression of uterine fibroids after castration, and two years later he and others reported the relief of symptoms due to prostatic hypertrophy by the same means (White, 1893, 1895 ; Hayden, 1895). Beatson (1896) described the beneficial influence of castration in two cases of advanced mammary carcinoma ; his work was rapidly confirmed, and for a decade or so the method achieved popularity. For various reasons which will be discussed later the practice became obsolete, but the records contain many instances of great benefit conferred by it (Beatson, 1896 ; Boyd, 1900 ; Thomson, 1902 ; Morris, 1902 ; Lett, 1905). The method of interrupting ovarian activity by means of X-rays came into use during this period (Halberstaedter, 1905), and was applied at a few centres to cases of advanced mammary carcinoma (de Courmelles, 1909, 1926 ; Wintz, 1926), but more general interest in the subject has been revived only during the past ten years, during which the work of Huggins and others on prostatic carcinoma (Huggins and Hodges, 1941), the application to clinical practice of pure substances with oestrogenic or androgenic properties, and the discovery of retardation or even temporary arrest of activity of various malignant lymphomas in man by means of nitrogen mustards and urethane have opened up fresh lines of inquiry (Rhoads, 1946 ; Paterson, Haddow, ApThomas and Watkinson, 1946). The present widespread use of androgens in cases of advanced mammary cancer dates from their administration, accompanied by castration, by Ulrich (1939*a*), while the beneficial effect of oestrogens in the same malady was first reported by Haddow, Watkinson, Paterson and Koller (1944).

In this paper the clinical aspects of androgen therapy in 70 cases of advanced mammary cancer in women are described, the literature surveyed, and the effect of androgens compared with that of castration carried out by surgery and by X-irradiation. The prophylactic use of androgens in mammary cancer as described by Prudente (1945) is not discussed here.

The investigation was begun in December, 1947, and since then sufficient experience has been gained to permit an assessment to be made in general terms of the place of androgens in treatment. Many problems of practical importance remain unsolved, such as the uncertainty in predicting success, the best preparation to use, the most effective dose, and the prevention of undesirable side effects. The most disappointing fact which emerges from the work is that sooner or later every case, even where no clinical evidence of disease remains, relapses in spite of continued treatment. Nevertheless, the extent and duration of remission is often sufficient to confer great benefit, and the utility of the therapy is beyond question.

The first 20 patients were treated by subcutaneous implantation of pellets of fused testosterone. Almost all improved as a result of this treatment, but it soon appeared that this improvement was unrelated to the progress of the disease, which often advanced unchecked. It consisted in an increased sense of well-being, improved appetite, weight gain, rise in haemoglobin value ; in other words it represented the benefit which the layman expects but rarely derives from a "tonic." Such non-specific benefit generally occurs whenever testosterone is given, and has unfortunately been used to justify testosterone therapy for other specific maladies as well as for mammary carcinoma. In the present series of cases a high percentage of the cases derived such benefit, but this has been ignored in assessing the results.

In only 6 of the first 20 patients was any specific benefit conferred, and it was decided that implantation as an initial method of administration was impracticable. However, when specific improvement did occur, it was already manifest within 2 months of implantation, and it therefore seemed desirable to defer implantation until the response to the hormone given by some other route had been ascertained. Implantation could then be considered in cases responding favourably, non-specific benefit being neglected. Two preparations were available, namely intramuscular testosterone propionate in oily solution, and methyltestosterone for sublingual absorption. The former has been widely used during the past 10 years for mammary carcinoma, but there are no published accounts of the large scale use of methyltestosterone, and it was decided to try this compound in subsequent cases. The early results were surprisingly good, and warranted extended trials of the substance. Most of the later cases were treated with methyl-testosterone. In a few instances testosterone implantation, sublingual methyltestosterone, and testosterone propionate injections were used in the same patient at different times, but not in sufficient numbers to permit a direct comparison of the methods. Lastly, testosterone propionate incorporated in an ointment was applied locally in a few cases of malignant ulceration of the skin.

### PREPARATIONS, ROUTES OF ADMINISTRATION AND DOSAGE.

The number of patients treated by testosterone, testosterone propionate, and by methyltestosterone, is set out in Table I. These three substances differ

TABLE I.—*Response to Different Androgen Preparations.*

| Preparation. | Success. | Moderate success. | Failure. | Total. |
|---|---|---|---|---|
| Testosterone (implant.) | 5 (3) | 7 (5) | 14 (6) | 26 |
| Methyltestosterone (sublingual) | 12 (5) | 8 (2) | 20 (7) | 40 |
| Testosterone propionate (intramuscular) | 2 (1) | 4 (3) | 3 (1) | 9 |
| Combined— | | | | |
| Test. and Meth. | 2 (1) | 1 (1) | .. | 3 |
| Test. and T. prop. | 0 | 0 | 0 | 0 |
| Meth. and T. prop. | 1 | 3 (2) | 2 | 6 |
| All three | 1 (1) | 0 | 0 | 1 |

Figures in brackets indicate cases with skeletal involvement. Cases treated concurrently with X-rays omitted.

considerably in their pharmacological properties. The androgenic potency of a compound can be expressed only in relation to the method of assay; for example, a given compound may be more potent than another in stimulating the growth of the seminal vesicles of immature rats, but less so when the weight of the prostate is measured for assay. In the main these differences probably reflect varying speeds of absorption, cumulation and excretion rather than inherent pharmacological differences. However, testosterone is usually stated to be a slightly less potent androgen than testosterone propionate, but more powerful than methyltestosterone.

*Testosterone.*

Subcutaneous implants in the form of 50 mg. and 100 mg. pellets of fused amorphous testosterone prepared from cholesterol (Butenandt and Hanisch, 1935) were used. In 18 cases 500 mg. was given, in 11 cases 1000 mg., 3 having received a previous implant of 500 mg. The pellets were inserted through a skin incision 1 cm. long in the lower abdominal quadrants, and pushed through radially to rest on the external oblique aponeurosis between 5 and 10 cm. from the incision, the pellets lying at points on a circle with the incision as centre.

Absorption from these pellets depends on a variety of local factors, including their relation to surrounding fat, adjacent fascia, lymphatic plexuses and encapsulation by fibrous tissue. In three patients who came to autopsy 55, 82 and 93 days respectively after insertion, the pellets were removed, allowed to dry for 48 hours and weighed. The weights of these pellets are set out in Table II, from which it is seen that absorption rate is not uniform. The smooth, thin, translucent fibrous tissue capsules in which the pellets were embedded were well established in the case in which implantation was carried out 55 days previously, and were no thicker as judged by naked-eye inspection in the case which came to autopsy 93 days after implantation.

TABLE II.—*Dry Weights of* 100 *mg. Testosterone Pellets Removed at Intervals after Implantation.*

| | Case 48.* | Case 11. | Case 65. |
|---|---|---|---|
| Days since implantation | 55 | 82 | 93 |
| Dry weight in mg. of recovered pellets | 35·6 | 44·7 | 40·8 |
| | 34·2 | 51·0 | 31·9 |
| | 29·3 | 53·5 | 26·4 |
| | 24·8 | 53·0 | 23·7 |
| | | 51·0 | 13·1 |
| Total unabsorbed testosterone (mg.) | 154·8 | 253·2 | 135·9 |
| Total absorption (mg.) | 345·2 | 246·8 | 364·1 |
| Mean absorption per pellet (mg.) | 69·0 | 49·4 | 72·8 |
| Greatest absorption | 75.2 | 55.3 | 86·9 |
| Least absorption | 64·4 | 46·5 | 58·2 |
| Variation | 10·8 | 8·8 | 28·7 |

* One pellet was not recovered from this case. In calculating total absorption the weight of the missing pellet was taken as the mean of the weights of the other 4.

Absorption from implanted pellets is maximal at the time of insertion, and then diminishes at a rate which is proportional to the rate of decrease of the surface area of the pellets. With ten 100 mg. pellets the maximal daily absorption probably never exceeds 10 mg. ; nevertheless the constitutional effects produced by this dose require about 500 mg. per month for their evocation when injections of testosterone propionate are used (Geist, Salmon and Gaines, 1938 ; Geist, Salmon and Walter, 1940), representing daily doses of nearly double the maximal (initial) daily absorption from ten 100 mg. pellets. It seems likely, therefore, that the injection of testosterone propionate is attended by considerable wastage, and that the much smaller quantity of hormone utilized by continuous slow absorption from implanted pellets more nearly represents the pharmacologically effective amount. This view has received experimental confirmation. Carlinfanti, D'Alo and Cutolo (1949) showed that the activity of a single dose of testosterone in preventing post-castration atrophy of the seminal vesicles of guinea-pigs could be greatly prolonged and also enhanced by preliminary adsorption on to aluminium phosphate. Absorption from the suspended particles may be compared to that from multiple small implants.

The observations on which the above conclusions are bases are admittedly few, and the variable absorption from implanted pellets, and the lack of control and of precise knowledge of absorption once implantation has been carried out, may be considered to limit the reliability of the method. But in practice it has yielded results of value, and against its disadvantages may also be set the facts that patients receiving injection therapy require repeated visits by district nurses, as few patients can be taught to give their own intramuscular injections, while sublingual therapy is open to the very real objection that administration is unsupervised and therefore less certain. Further, the question of expense cannot be ignored, and at present the method of implantation is the most economical way of giving androgens provided it is confined to cases already known to respond to androgen.

In retrospect the dose of 500 mg. by implantation which was given 18 times appears insufficient, but implantation of 1000 mg. (10 × 100 mg.) is probably sufficient to secure specific response in mammary carcinoma. It is recommended as the method of choice for maintenance therapy in cases which have shown a satisfactory response to intramuscular or oral therapy, but should not be used without a preliminary trial, owing to the high percentage (80 per cent) of cases which fails to respond to androgen therapy in any form. No instance of pellet extrusion occurred.

*Testosterone propionate.*

This substance in oily solution for intramuscular injection has been the most widely used androgen preparation in cases of mammary carcinoma. In the earlier reports doses of the order of 25–50 mg. were given 3–7 times weekly, but later opinion came to advocate larger and larger doses of the order of 100 mg. daily, or even twice daily. The authors of the latest progress report of the Council on Pharmacy and Chemistry (1949), however, no longer favour heavy dosage, and consider that there is no advantage in exceeding 150 mg. weekly, this view being based on the results obtained in 285 cases in which dosage schedules between 25 mg. and 200 mg. thrice weekly were employed.

In the present series only 9 cases have received testosterone propionate, in all but two instances it was used after methyltestosterone had failed to initiate or maintain a remission, and in only 2 of the 9 cases was it effective in any degree.

In every case the dose given was 100 mg. daily for the first fortnight; one case received no further treatment, 4 others continued daily injections for another month, the remaining 4 continuing on 3 injections weekly for 6–10 weeks.

### 17-*Methyltestosterone.*

This compound is more readily absorbed from mucous membranes than either the pure hormone or its esters, but is a more feeble androgen. Judged by its effect on menstruation, on the skin, hair and general metabolism, it is about 1/5 as active weight for weight as testosterone, and its administration is even more wasteful than that of testosterone propionate. However, in this country it is less expensive in the doses here recommended than the propionate, but more so than testosterone implantation. Of the 44 patients who were given methyltestosterone, 28 received 50 mg. daily for indefinite periods between 3 months and 1 year; the remainder received larger doses, 100 mg. daily being the largest given. The present tendency is to give 100 mg. daily for the first 2–4 weeks, to halve this dose if the patient is responding favourably, or to continue for another month if there is no evidence of specific benefit. All 30 patients who derived benefit from androgen therapy showed clear evidence of it within 8 weeks, and in most cases within the first month. No patient who failed to respond after 8 weeks of treatment did so after a longer interval, and it is probably not worth continuing treatment after 10 weeks in the absence of specific response. It cannot be said from the small number of cases in this series whether such cases should be transferred to testosterone propionate injections, but the general impression gained has been that patients likely to benefit substantially from androgen therapy are extremely sensitive to its action, in whichever form it is given, and do as well on doses just large enough to suppress menstruation in the average premenopausal woman as they do on much larger doses. On the other hand, most cases which fail to respond to the smaller doses do no better when the dose is increased or the route of administration changed, but the minority which does so led to the tendency already mentioned to advocate heavy dosage.

Patients given methyltestosterone were advised to leave the tablets (50 mg.) under the tongue, and to allow them to dissolve without chewing or swallowing. The time taken for solution varied between 10 and 30 minutes in different individuals, and a few patients disliked the bitter taste of the substance.

### *Duration of Treatment in Cases Responding Favourably.*

No definite recommendation can be given with regard to the length of time administration should be continued. Cutler and Schlemenson, reporting 19 cases, advised continuing injections until palliation was no longer being obtained (Cutler and Schlemenson, 1948). The American Council already referred to found that the most favourable results were obtained when injections were continued until a total of 3·0 g. had been given. In this series it has frequently been observed that lesions which heal during treatment do not later break down when recurrences appear at other sites, whether treatment is still in progress or not. Also, in 2 cases (25 and 52) no treatment other than a single implantation of 1000 mg. and 500 mg. respectively was given. In Case 25, malignant pleural effusions and pulmonary deposits having resolved, the patient is recurrence-free 14 months later, while in Case 52 there has been no recurrence of back pain and no clinical or radiological evidence of extension of the disease 18 months later. On the other hand, a majority of cases relapsed in spite of continued treatment. In a few instances the disease process even appeared to be accelerated by continued treatment. In Case 37, reported more fully in the Appendix, dramatic improvement followed implantation of 500 mg. (10 × 50 mg.) testosterone, was maintained by a second implantation of 1000 mg. (10 × 100 mg.) four months later, and by 50 mg. methyltestosterone daily, commenced 6 months after the second implantation and continued for a further 4 months. Widespread evidence of fresh activity of the carcinoma then appeared, accompanied by recurrence of pain and general deterioration. Testosterone propionate injections 100 mg. daily were then given over a period of 6 weeks without any benefit, after which androgen therapy was discontinued. Since then the patient's general condition has again improved, and regression of cutaneous, skeletal and lymph node metastases has occurred, the original implantation having been carried out 20 months ago. Adair (1949) recorded a case in which treatment was continued for many months after initial improvement in spite of deterioration. Cessation of treatment was almost immediately followed by striking and maintained remission. These facts suggest that there is a limit to the amount of testosterone which can be usefully given, beyond which further administration is unlikely to be of value. If a lesion has regressed it will not recur. If it has only partially regressed it will resume its activity whether more hormone is given or not. In a patient with healed lesions, more testosterone will not prevent recurrence at other sites. Further observations are required.

### THE SIDE EFFECTS OF ANDROGEN THERAPY.

These include the beneficial effects which have been referred to earlier, as well as the less desirable masculinizing effects and other disturbances. Evidence will be given later indicating that the masculinization which is a common accompaniment of testosterone therapy is not a necessary condition for success in the treatment of mammary carcinoma. It is therefore included as a " side-effect." The chief side-effects may be grouped as (1) oestrogen withdrawal effects, (2) signs of masculinization, and (3) metabolic effects.

*Oestrogen withdrawal effects.*—These are observed within the first month of treatment, and consist of hot flushes, regression of the endometrium with amenorrhoea, and conversion of the vaginal epithelium to the " oestrin-deficient " stage (Papanicolaou, Ripley and Shorr, 1938). They are met with in cases of surgical castration, and somewhat later in cases of X-ray induced castration.

*Signs of masculinization.*—These become prominent during the second 3 months of treatment, and consist in order of appearance in coarsening and increased sebaceous activity of the skin, facial hirsuties, acne, increased growth of hair on the limbs, huskiness of the voice and enlargement of the clitoris (Geist, Salmon and Walter, 1940 ; Zuckerman, 1937 ; Foss, 1938).

*Metabolic effects.*—Several symptoms and signs are to be ascribed to metabolic changes, which include re-mineralization of the bones (Farrow and Woodard, 1942), retention of water, electrolytes and nitrogen (Kenyon, Sandiford, Hughes, Knowlton and Koch, 1938 ; Abels, Nelson, Young and Taylor, 1944), and others less easy to define. The patients become alert and active, and gain strength and weight. They may complain of swelling of the ankles. When hypercalcaemia occurs the patients complain of drowsiness, thirst, headache, nausea and vomiting.

In one patient with hypertension, the onset of angina of effort which seemed to be precipitated by testosterone therapy was probably due to the increased activity and weight gain increasing the load of a heart already failing (Case 16). This may have been the precipitating cause in 5 of 24 cases reported by Taylor, Slaughter, Smejkal, Fowler and Preston (1948), in which congestive cardiac failure followed androgen therapy.

These phenomena provide a rough measure by which the absorption and activity of the different preparations may be compared. Thus implantation of 500 mg. testosterone was followed by amenorrhoea for 4 months in both patients in whom it was carried out. Implantation of 1000 mg. led to amenorrhoea lasting 7 months. With ten 100 mg. pellets the maximal absorption of testosterone is probably less than 10 mg. daily. By contrast daily administration of 50 mg. methyltestosterone sublingually caused amenorrhoea in 7 out of 11 women, menstruation being unaffected in 1, reduced in another, and disturbed in the remaining 2. Forty mg. daily was without effect on menstruation. Doses over 80 mg. induced amenorrhoea. As far as the effect on menstruation is concerned, it may therefore be said that the hormone absorbed from 50 mg. sublingual methyltestosterone is less potent than 10 mg. of testosterone subcutaneously. With varied doses it has also been possible to compare the thresholds for the appearance of different side-effects. Thus patients who received 500 mg. testosterone implants almost all gained weight in the first month, in one case as much as 8 lb., but skin changes were never marked with this dose, facial hirsuties developed in only 2 out of 18 cases, and voice change was marked in only 1 case. Of the 3 fatal cases from which the pellets were recovered, one gained 7 lb. in weight during the first month following implantation, and at the time of death 9 weeks later had still absorbed only 364·1 mg. testosterone (Table II). Marked facial hirsuties was present in the second of these cases at the time of death, 12 weeks after implantation, not more than 250 mg. testosterone having been absorbed. These changes occurred much more often in patients who received 1000 mg. implants, and in those who took sublingual methyltestosterone in doses over 50 mg. daily. In the case of implantations the effective dose diminishes every day, but even with 500 mg. amenorrhoea persisted for 4 months, and weight gain continued during the same period. Weight gain, and subjective benefit, occurred without other side-effects in patients who were given methyltestosterone in 40 mg. doses daily. It therefore appears that the production of masculinizing effects requires larger and more prolonged dosage than that necessary to initiate the metabolic changes responsible for increased activity, appetite, sense of well-being, and weight gain ; inhibition of menstruation requires intermediate dosage. The order of appearance of these effects in the individual patient runs parallel to their incidence in groups receiving different doses.

*The " Tonic " Effect of Testosterone.*

The speedy improvement brought about by testosterone therapy even in debilitated patients is very striking. Weight gains of 5 lb. in the first month are common, and with the other " tonic " effects are independent of the specific effect of the hormone on mammary carcinoma. In Case 65 for example 7 lb. weight gain occurred in the first month, during which rapid extension of the disease in chest wall, regional lymph nodes and opposite breast occurred. Weight gain may reach embarrassing levels. One patient (Case 35) gained steadily at the rate of 3 lb. per week, and in 3 months had put on 32 lb. Another (Case 44, Appendix) gained 23 lb. in the first 4 months, and a further 8 lb. in the second 4 months. Clinical oedema, however, was seen in only 3 cases, and was always slight in amount, and confined to the ankles and lower half of the legs. In a few of the patients with post-mastectomy oedema of the arm, an increase in the swelling of the arm followed testosterone administration, but this was more closely related to spread of the disease than to the treatment, for when lesions regressed, the lymphoedema sometimes diminished, even when rapid increase in weight was occurring, as in Case 44. During the initial period of weight gain, some of the patients stated that they passed less water, but no measurements have been carried out.

Much of the initial rapid weight gain is due to the effects of electrolyte, and particularly sodium retention, which holds equivalent quantities of water in the body. This effect is a feature of the activity of many steroid hormones, notably desoxycorticosterone, progesterone, oestradiol, and of Kendall's compounds E and F. When administration of the hormone is discontinued, much of the rapidly gained weight is lost, and if it is continued the rate of weight gain diminishes and finally comes to a standstill. Part of the weight gain is due to increased appetite, and in sick patients who obtain specific benefit, improvement in general health also contributes, and extra tissue, especially fat, is laid down.

Rise in haemoglobin occurs during the first few months of treatment, and may be considerable, even when widespread skeletal metastases are present.

*The Effect of Testosterone and Methyltestosterone on Menstruation.*

The number of menstruating women in this series is small, and the results insufficient to permit a full account of the inhibiting action of these androgens. The effect of testosterone propionate has been fully studied by others (Papani-

TABLE III.—*Effect of Testosterone and Methyltestosterone on Menstruation.*

| Day of menstrual cycle treatment commenced. | Age (years). | Preparation and dose. | Next menstrual period. |
|---|---|---|---|
| 3 | 43 | Methyltestosterone 50 mg./d. | Absent. |
| 3 | 48 | Methyltestosterone 50 mg./d. | ,, |
| 3 | 37 | Methyltestosterone 50 mg./d. | Slight "staining" for subsequent 16 days. Next period absent. |
| 4 | 37 | Testosterone implant. 1000 mg. | Absent. |
| 5 | 42 | Methyltestosterone 100 mg./d. | ,, |
| 7 | 40 | Methyltestosterone 50 mg./d. | Next period 8 days early (14 days after). Following period also 8 days early. |
| 9 | 43 | Methyltestosterone 80mg./d. | Reduced in amount but punctual. Following period absent. |
| 10 | 37 | Testosterone implant. 500 mg. | Absent. |
| 11 | 37 | Methyltestosterone 50 mg./d. | Next period 8 days early (11 days later). Following period absent. |
| 16 | 48 | Methyltestosterone 50 mg./d. | Absent. |
| 26 | 39 | Methyltestosterone 50 mg./d. | Slight " staining " for $\frac{1}{2}$ day on 1st day of next 2 periods. |

colaou, Ripley and Shorr, 1938 ; Geist, Salmon and Walter, 1940). In 11 women with regular periods the day on which testosterone therapy was begun was noted, counting the first day of the last menstrual period as day 1. The effect on the next expected menstrual period is indicated in Table III. The next period may be suppressed, reduced in amount of bleeding and duration, or may be brought on about a week early. In one of the cases where methyltestosterone was given during a menstrual period (on day 3), slight bleeding continued until the 19th day of the cycle, the next period being suppressed. Amenorrhoea persists as long as administration of the hormone is continued at the necessary dosage, the periods returning regularly about a month after withdrawal. One patient (Case 37) in whom amenorrhoea lasted for a year had a single " period " a fortnight after treatment was finally discontinued, but has not bled since.

*The Masculinizing Effects of Androgens.*

The skin and voice changes which almost always follow continued administration of androgens are both unpleasant and embarrassing for the patients, but are usually tolerated without complaint when much specific improvement occurs. These changes usually became marked during the third month of treatment, but much variation in their intensity was observed, and a few patients escaped some or all of them. Routine examination of the genitals was not carried out, but in a few patients hypertrophy of the clitoris was noted, involving the shaft, glans and hood, under which much smegma had accumulated.

*Skin changes.*

The primary skin changes are increased activity of the sebaceous glands (Roug and Zakon, 1943), of the hair follicles, and increased keratinization, more particularly at the mouths of the lanugo follicles. The seborrhoea is often severe, especially in dark complexioned subjects, and on the scalp pityriasis capitis may develop. The texture of the skin becomes coarser, the follicles become more prominent and sooner or later some become infected, and a punctate folliculitis ensues, being most marked in the interscapular and intermammary regions. On the forehead and chin the lesions tend to be scantier but larger, and the patients complain of recurrent crops of " pimples." The general coarsening of the skin, with prominent " pores," especially on the forehead, cheeks, chin and sides of the nose sometimes alters the facial appearance of the patient sufficiently to excite the comment of relatives or friends. The skin condition resembles pustular acne, but differs from it in that comedones rarely develop, even when the lesions persist for many months. In two patients only (Cases 63 and 44) did true comedones appear after taking methyltestosterone, 50 mg. daily for 6 months and 1 year respectively. They were abundant across the back, and were also present on the cheeks and chin. The skin, which was heavily infected, had the lumpy appearance typical of adolescent pustular acne. In Case 44 hirsuties was minimal, consisting of a sparse growth of soft hairs on either side of the upper lip, but was much more developed in Case 63. In 7 cases facial hirsuties reached an extreme degree, the sides of the face being covered with thick downy hair between 1 and 2 cm. long (Cases 7, 9, 24, 25, 56, 63). In Cases 9 and 56, after 6 months' treatment the hairs increased in calibre, and resembled the stiff bristles of the male beard. There was some variation in the changes in the scalp hair. Some patients maintained that their hair grew more vigorously, and more thickly, while others averred that their hair "fell out in handfuls." In two cases (25 and 63) marked hair recession in the temporal region was observed. Increased growth of body hair was less commonly seen, but was marked in two cases, both with dark hair (46 and 56), the shoulders, abdomen, forearms, thighs and legs being heavily affected. Apart from these two cases, upward spread of pubic hair towards the umbilicus was not met with.

*Voice changes.*

Voice changes occurred only in a proportion of patients with relatively high-pitched voices. In only 12 was definite lowering of pitch recorded, but lesser degrees of change were noted in several others. The first evidence of change consists in slight huskiness, which may become obvious only after the voice has been used for some time. The condition may then remain stationary, or may progress to more permanent huskiness, and in a smaller number of patients lowering of pitch with increased resonance ensues.

*Duration of effects.*

The persistence of the skin changes is dependent on a continued supply of androgen, and the lesions clear up within two months of withholding the supply of methyltestosterone. The hirsuties remains longer, but rarely persists 6 months after stopping treatment. The voice changes are more permanent, and may last for a year or more. It is difficult to explain the mechanism of the voice change, but its persistence suggests some structural alteration in the larynx. Radiographs have not revealed any changes in calcification of the laryngeal cartilages. In two instances (Cases 43 and 63) the skin condition was most advanced at the end of the third month of treatment, and then regressed considerably in spite of continued treatment with methyltestosterone.

### *Toxic Effects.*

The side effects that have been described above are at most inconvenient, but in themselves are not harmful. On occasion, however, androgen administration can precipitate toxic symptoms of some severity. When fully developed they form a characteristic syndrome, which has been observed in 3 patients in this series. Lesser degrees of it were met with in 7 others. It was first seen when 2 patients (Cases 10 and 45) were given 80 mg. methyltestosterone daily in 5-mg. tablets, both patients having been treated 5 months previously by implantation of 500 mg. testosterone. In Case 10, after taking the tablets for 2 days, the patient complained of extreme weakness, drowsiness, severe headache, nausea, and increase in the dyspnoea which was her initial complaint, and due to pulmonary and pleural metastases. The symptoms subsided rapidly after she stopped taking the tablets. In Case 45 the patient took the tablets for 5 days, during the last 3 of which she felt generally ill, nauseated, and complained of increase in the severity of her backache. On the 5th day she was distressed by vomiting, abdominal distension and pain, and took no more tablets, after which her condition improved rapidly. One week later she again attempted to take the tablets, but with exactly the same result. Case 30 was a woman of 45 who had been previously treated for 2 years by repeated doses of X-rays for widespread skeletal recurrences for a Stage IV carcinoma, and had had induction of an artificial menopause. She was given methyltestosterone 50 mg. daily for one month, towards the end of which she complained of increase in the severity of her pains, dizziness, nausea and vomiting, and general malaise. The symptoms abated within a day or two of discontinuing the drug. She was asked to resume the tablets a fortnight later, but the symptoms recurred after one week. In Case 47, doses of 35 mg. methyltestosterone regularly induced anorexia, extreme thirst, dizziness, headache, fever, nausea and vomiting, these symptoms decreasing rapidly when the substance was withheld. In the mildest cases drowsiness or depression were the only symptoms, and cases intermediate in severity occurred also. This syndrome has been ascribed to the flooding of the circulation with calcium ions mobilized from the bones, and is said to be precipitated more readily by testosterone when the serum calcium level is initially high (Farrow and Woodard, 1942). Similar symptoms occur when large doses of calciferol are administered over a long period, and are also associated with elevation of serum diffusible calcium level (Anning, Dawson, Dolby and Ingram, 1948). The present cases have not provided additional information on this point.

*Gastric symptoms.*

Five patients (Cases 4, 7, 15, 52, 70) developed gastric symptoms during testosterone therapy. Three of them (Cases 4, 52, 70) had a previous history of peptic ulceration, and in all three investigations indicated active ulceration. In one of these patients (Case 52) the testosterone was implanted, but the other 4 took sublingual methyltestosterone. The two patients with no previous ulcer history complained of heartburn, acid regurgitation and eructations coming on about 30 minutes after each meal, lasting for another half hour unless relieved by alkalis. The symptoms were relieved by a few weeks on a milk diet, the testosterone being continued.

*Cardiac symptoms.*

Taylor, Slaughter, Smejkal, Fowler and Preston (1948) refer to the onset of tachycardia, tremor and congestive cardiac failure in their report on hormone therapy. In the present series only one case (Case 16) showed any evidence of an adverse effect on the heart, and as already suggested, this was in all probability only a secondary effect due to rapid weight gain.

### *Relation of Side-effects to Specific Effect.*

The specific effect of testosterone is not directly dependent on any of the side-effects described. Some of the best results were obtained in patients who did not develop marked signs of masculinization, while in Case 20 regression of the disease had begun with a dose of methyltestosterone which did not suppress menstruation (40 mg. daily). On the other hand several of the patients who gained weight most rapidly, and later developed gross signs of masculinization, never obtained the slightest specific benefit, and in some the disease progressed rapidly.

### Clinical Material.

All the patients referred for androgen therapy were in an advanced stage of the disease. Fifteen were Stage IV cases when first seen, 35 cases had severe symptoms, mostly generalized skeletal pain or dyspnoea, and 44 cases were found at follow-up to have recurrences at various sites unsuitable for excision, or unlikely to respond to further irradiation. An impression of the composition of the series may be gained by the classification into 3 groups based on a general clinical assessment at the start of androgen therapy.

*Group I : Good general condition.*—In the first group are 40 patients in good general condition, with no recent weight loss, no anaemia, afebrile, and who would describe themselves as fit except for the particular symptom or sign requiring treatment. Thus 4 cases whose only complaint was dyspnoea are included in this group. Patients with limited disability due to bony metastases are also placed in it, for example Case 4 with a painful right shoulder, Case 70 with stiff neck, and 3 patients otherwise fit, but bedridden because of severe localized backache, with paraplegia in one case (Cases 7 and 60). Most of the patients in whom skin recurrences predominated, whether ulcerated or otherwise, also fall into this group.

*Group II : Poor general condition.*—The second group is composed of 20 cases in poor condition at the commencement of therapy, deteriorating rapidly with regard to appetite, weight, anaemia and general strength. Seven patients in this group were bedridden, and all the others were leading a sedentary existence and unable to look after themselves. Ten had widespread skeletal metastases with severe pain.

*Group III : Fair condition.*—The remaining 10 patients were intermediate in general health between the two groups just described; all but one were ambulant, though complaining of varying degrees of weakness, anorexia, weight loss and malaise. The bedridden patient in this group had cerebral metastases causing headache, vomiting and ataxia (Case 53). In the whole series 31 patients had multiple bone deposits, 12 had intrathoracic recurrence, 30 had lymph-node involvement, 28 more or less extensive skin recurrences. Bone deposits were found radiologically in 6 patients who had not complained at any time of pain, and were probably present in others who were not X-rayed.

*Age composition.*

The age distribution is shown in Table I. Half of the patients were below 50 years, and 2 patients between 50 and 54 were still menstruating at the commencement of treatment. The youngest patient was 35 years, but 12 others were less than 40 years of age.

Twenty-two patients were premenopausal, 11 had had previous induction of artificial menopause, 37 had ceased to menstruate more than 1 year before commencing androgen therapy.

*Recurrent cases. Time since radical surgery.*

It has been suggested that a relation exists between response to androgen therapy and the interval since radical surgery. In this small series it has not been possible to isolate this relation from the age factor and from the menopausal factor, but some of the figures are given (Table I).

Fifteen cases presented with recurrence within 1 year of radical surgery. All but 2 of these were below 50 years of age, and only 3 responded favourably to androgen therapy, the disease progressing rapidly in the remainder, with widespread blood-borne dissemination in most instances.

Twenty-nine cases were alive more than 3 years after radical surgery, 13 between 3 and 5 years, 12 between 5 and 10 years, and 4 over 10 years. In 6 of the 14 cases with the best response to testosterone therapy the disease had recurred more than 3 years after radical surgery, in 7 others under 3 years after, in 1, 10 years after surgery.

*Parity.*

Thirty-four patients were childless, 13 of whom were married before the onset of the menopause. In 5 patients the parity was not recorded.

### *Classification of Results.*

In Table IV the results of treatment have been set out in 3 groups, namely "success," "moderate success" and "failure." By a "success" is meant a case in which relief of specific symptoms was accompanied by objective evidence of improvement and restoration of function, the patient resuming her normal activities, or in which conspicuous and lasting regression of lesions occurred. Eight of the 14 cases scored as "successes" remained well for over 1 year, 4 are still in remission more than 1 year after commencing treatment, the disease being again active in the other 4. Three others relapsed between 6 and 12 months after commencing treatment, and the remaining 3 are still in remission 3–6 months later. By a "moderate success" is meant a case in which symptomatic relief was short-lived (mostly less than 6 months), or not accompanied by considerable objective evidence of regression or by restoration of function. All other cases are classed as failures, even when non-specific benefit was marked and increased the patients' range of activity. Among the failures are included a few patients in whom follow-up was for one reason or another inadequate, and 11 in whom treatment had been abandoned in less than 2 months.

TABLE IV.—*Response to Androgen Therapy in Relation to Menopause and to General Clinical Condition before Treatment.*

| | I. Good condition. | | | | II. Poor condition. | | | | III. Fair condition. | | | | Total. |
|---|---|---|---|---|---|---|---|---|---|---|---|---|---|
| | Relation to menopause. | | | Total. | Relation to menopause. | | | Total. | Relation to menopause. | | | Total. | |
| | Pre- | Artificial menopause. | Post-(natural). | | Pre- | Artificial menopause. | Post (natural). | | Pre- | Artificial menopause. | Post-(natural). | | |
| Success | 3 | 2 | 2 | 7 | 3 | 0 | 2 | 5 | 2 | 0 | 0 | 2 | 14 |
| Moderate success | 3 | 2 | 4 | 9 | 0 | 0 | 3 | 3 | 1 | 0 | 3 | 4 | 16 |
| Failure | 2 | 4 | 14 | 20 | 2 | 2 | 7 | 11 | 2 | 1 | 1 | 4 | 35 |
| Radiotherapy as well | 4 | 0 | 0 | 4 | 0 | 0 | 1 | 1 | 0 | 0 | 0 | 0 | 5 |
| Total | 12 | 8 | 20 | 40, | 5 | 2 | 13 | 20 | 5 | 1 | 4 | 10 | 70 |

### *Duration of Observations.*

It will be seen from Table V that of the 59 patients followed up for more than 3 months, 38 have been observed for 6 months or longer, of whom 16 were observed for over 1 year after first receiving androgen therapy. Three of the 5 patients observed for over 18 months are classified as "successes." One of them (Case 37) is of great interest and is more fully described below. The other two patients observed for more than 18 months (Cases 18 and 28), one aged 68, and one aged

TABLE V.—*Duration of Observations* and Length of Remission.*

| Months. | 3–6. | 6–12. | 12–18. | 18+. | Total. |
|---|---|---|---|---|---|
| Total treated | 21 | 22 | 11 | 5 | 59 |
| Failure | 12 | 11 | 4 | 2 | 29 |
| Total remissions | 9 | 11 | 7 | 3 | 30 |
| Still in remission | 3 | 3 | 2 | 2 | 10 |
| Relapsed | 6 | 8 | 5 | 1 | 20 |

* Excluding 6 patients observed less than 1 month (classed as failures in Tables III and IV) and 5 patients treated concurrently with X-rays.

71, are both classified as failures. They were transferred to oestrogen therapy after 6 months' trial of androgens, and both responded well.

Of the 30 patients who derived benefit from the treatment, 21 obtained remissions lasting 6 months or more, 10 of them over 1 year, and 3 over 18 months. Twenty patients have relapsed between 3 and 18 months later, but 10 are still in remission at the time of writing, 4 of them 12 months or more after institution of therapy. By a relapse is meant any evidence of renewed activity of the disease, whether or not accompanied by symptoms. Case 37 has been classed as a relapse, although she has had a second remission since androgen therapy has been withheld. Cases 35 and 44 are also classed as "relapses" although both are in normal health, the former on account of a lymph node found at follow-up, and the latter because of two small recurrences in an otherwise healed chest wall ulcer.

### *General Survey of Results.*

It will be seen from Tables IV and VI that 30 of the 70 cases showed some specific response to androgen therapy, although only 14 of these have been classed as "successes." Among the 22 pre-menopausal cases were 8 successes, whereas there were only 4 successes among the 37 patients who had passed the menopause. Of 11 cases who were given androgens after the induction of artificial menopause, only 2 could be counted as successes. Of the 15 Stage IV

TABLE VI.—*Distribution of "Successes" in Relation to Age, Menopause, and Time since Radical Surgery.*

| Age group. | | Relation to menopause. | | | | Cases with recurrence. Time from radical surgery. | | | | |
|---|---|---|---|---|---|---|---|---|---|---|
| | | Pre- | Artificial menopause. | Post-(natural). | Stage IV cases. | 1 year and under. | 1–3. | 3–5. | 5–10. | Over 10. |
| 30–4 | 0 | 0 | 0 | 0 | 0 | 0 | 0 | 0 | 0 | 0 |
| 35–9 | 13 (4) | 8 (3) | 4 (1) | 1 | 2 (1) | 6 (1) | 3 | 2 (2) | 0 | 0 |
| 40–4 | 8 (2) | 6 (1) | 1 (1) | 1 | 0 | 2 (1) | 4 (1) | 2 | 0 | 0 |
| 45–9 | 15 (5) | 6 (5) | 4 | 5 (1) | 4 (1) | 5 (1) | 2 | 1 (1) | 3 (3) | 0 |
| 50–4 | 18 (1) | 2 | 2 | 14 (1) | 5 | 2 | 1 | 5 | 3 | 2 (1) |
| 55–9 | 8 (1) | 0 | 0 | 8 (1) | 3 | 0 | 1 (1) | 1 | 2 | 1 |
| 60–4 | 3 (1) | 0 | 0 | 3 (1) | 0 | 0 | 1 (1) | 1 | 0 | 1 |
| 65–9 | 3 | 0 | 0 | 3 | 0 | 0 | 1 | 1 | 1 | 0 |
| 70–4 | 2 | 0 | 0 | 2 | 1 | 1 | 0 | 0 | 0 | 0 |
| 75–80 | 0 | 0 | 0 | 0 | 0 | 0 | 0 | 0 | 0 | 0 |
| Total | 70 | 22 | 11 | 37 | 15 | 16 | 13 | 13 | 9 | 4 |
| "Successes" | 14 | 9 | 2 | 4 | 2 | 3 | 3 | 3 | 3 | 1 |

Figures in brackets indicate the number of "successes."

cases previously untreated, only one (Case 20) responded successfully to androgen therapy, the only other success in this group having had an artificially-induced menopause.

Of the 30 patients who obtained specific benefit from androgens, 16 were cases with predominantly skeletal involvement, 7 of which (4 of them pre-menopausal) have been classed as successes.

From Table IV it is seen that specific response to therapy is independent of the general condition of the patient at the commencement of therapy. Some of the most dramatic effects of androgens have occurred in patients grossly debilitated and bedridden. Thus there were 7 successes among the 35 patients in Group 1 (good general condition), 5 among the 19 in Group II (poor general condition), and 2 among the 10 patients in the intermediate group.

*Cases with symptoms of skeletal involvement.*

As others have noted, the most gratifying results of androgen therapy have been in cases with skeletal deposits giving much pain. The relief of pain often came within a few days of commencing treatment, even when the pain had been present for many months beforehand, the patients crippled and requiring repeated doses of opiates. Sixteen out of 28 cases with skeletal pain obtained some relief of symptoms, but in 7 of these the effect can only be described as spectacular. Three bedridden patients were able to walk within a fortnight of commencing treatment, and were pain-free within a month, and able to return to their normal activities. Case 37 is described in detail below ; she remained in good health for 15 months, after which the pains recurred. The other 2 cases are symptom-free 14 months and 6 months after commencing treatment with testosterone. Two other patients severely handicapped by skeletal pain, though not bedridden, were able to resume their normal activities after receiving testosterone, and are symptom-free 8 and 18 months later. One, Case 4, obtained rapid initial relief of pain in the shoulder associated with a large osteolytic metastasis at the medial end of the clavicle after taking 50 mg. methyltestosterone daily. Five months later her condition deteriorated ; 6 months later she developed gross mediastinal obstruction. The shoulder pain has not recurred. She was transferred after 5 months to injections of 100 mg. testosterone propionate three times weekly without benefit after 6 weeks. She is classed as a " moderate success " in view of the initial response. The remaining patients obtained less benefit, and 3 were never able to leave their beds, although all volunteered that their pains were improved. One of these declared that she had not felt so well nor had so little pain for 2 years, 20 months of which had been spent in bed.

Case 66 was a woman of 44 years with extensive metastasis to the spine which was responsible for gross collapse of the bodies of dorsal vertebrae 7–11. She had been unable to walk or stand for 6 weeks owing to severe proprioceptive loss in both legs, a short course of radiotherapy having conferred no benefit. A mild paraplegia was present, and there was some impairment of sensation to light touch, deep pressure and pinprick from the level of the umbilicus downwards. There was, in addition, a girdle of almost complete anaesthesia to touch and pinprick over the distribution of dorsal segments 7–10. Curiously, sensation over the paravertebral regions of these segments was normal. There was no hyperalgesic zone, and no disturbance of sphincter control. The patient was given 28 daily injections of 100 mg. testosterone propionate intramuscularly, followed by 3 weekly injections for 2 weeks, and a further month of 2 injections weekly. During this period considerable improvement in proprioception occurred, the signs of paraplegia became less, though both plantar responses remained equivocal and right ankle clonus persisted, while perception of touch and pinprick returned to normal. She was able to stand with moderate rombergism, and after 3 months could walk 100 yards with one assistant. Whether this improvement reflects any specific effect of the hormone, or not, cannot be said, but it began within a month of starting treatment, whereas her condition was stationary during the preceding 6 weeks. After hesitation it has been decided to include her as a " moderate success."

*Cases with symptoms of intrathoracic involvement.*

Three patients whose main complaint was severe dyspnoea obtained relief after testosterone treatment. Large effusions were present in each case, bilateral in one (Case 63), and with multiple bilateral parenchymal deposits in another (Case 25). In each case the symptomatic relief was complete, and the patients were able to resume their normal activities. In Case 24 little change in physical signs or X-ray appearance occurred, but in the other 2 cases the effusions were almost completely absorbed, and in Case 25 the pulmonary deposits disappeared (Fig. 1 and 2). Case 24, with widespread recurrences in other organs, relapsed 9 months later, but the other 2 are free of symptoms and signs, and leading normal lives 8 months and 14 months later. Two other patients with dyspnoea as one of several symptoms obtained some relief after treatment. In Cases 3, 10, 24 and 63 the rapidity with which the breathing improved was astonishing, and comparable with the speedy relief of bone pain in the cases mentioned above. In Case 25, however, with dyspnoea at rest, no significant improvement had occurred after a fortnight. Ten oz. of fluid were aspirated from the left pleural cavity with some relief, and during the next two months rapid absorption of the remaining fluid and resolution of the lung deposits took place (Case 25, Appendix). A similar case in which relief of dyspnoea, absorption of effusion and resolution of parenchymal deposits followed removal of both ovaries has been described (Jochweds, Baranowicz and Horecki, 1948), and resolution of parenchymal deposits in a woman of 70 years has been described by Taylor, Slaughter, Smejkal, Fowler and Preston (1948).

*Symptoms of intracranial involvement.*

Adair (1949) has described a case in which Jacksonian attacks ceased, and physical signs improved, following testosterone administration. In the present series only 2 cases with clear evidence of cerebral metastases have been treated. Case 53, a nullipara, aged 44, remained well for 2 years after radical operation, and then developed frequent headaches and vomiting with progressive ataxia of the left arm and leg. Within a week of testosterone implantation (1000 mg.) the headaches and vomiting ceased, and decided improvement in the power and precision of movement of the left arm and hand took place ; the headaches and vomiting did not recur, but 2 months later the onset of severe backache and leg pains marked the beginning of a rapidly fatal downhill course. Case 43, a nullipara, aged 54, had remained well for 9 years after radical surgery when she developed pain over the sternum and double vision. The latter was associated with a conjugate defect of elevation, thought to indicate a lesion in the anterior part of the oculomotor nucleus. This lesion could have been of vascular origin, or might have been due to a metastasis. That the latter was the more likely is suggested by the development 6 months later of diminished sensation to touch and pinprick over the right trigeminal distribution. This patient took methyltestosterone 50–100 mg. daily for 6 months, but derived non-specific benefit only, the strabismus persisting, and further evidence of intracerebral involvement developing, as already indicated.

Seven cases developed cranial nerve lesions, which most probably resulted from local compression by cranial or dural deposits of growth. Four patients complained of numbness of the lips and tongue, and were found to have diminished sensation to touch and pinprick and deep pressure over areas of maxillary or mandibular nerve distribution (Cases 30, 49, 51 and 54). In addition 2 developed deviation of the tongue, with fibrillation and wasting (49, 51), and at autopsy one of these (49) was found to have hemi-atrophy of the tongue with fibrous replacement. The skull and dura were extensively infiltrated by growth, but a region of compression of the hypoglossal nerve was not found. Unilateral oculomotor palsy, facial anaesthesia and facial palsies were present in 2 cases (Cases 62 and 67), and one case developed unilateral nerve deafness 15 months after commencing testosterone treatment, at a time when widespread evidence of resistance to the hormone was appearing, including multiple bosses on the vault of the skull (Case 37). In none of these cases was any improvement in the cranial nerve affections noted as a result of testosterone treatment.

### *Objective Evidence of Benefit.*

Included in this section are instances of regression, partial or complete, observed in cutaneous recurrences, infiltrated lymph nodes, and localized bony swellings, and of healing of malignant ulcers. The histology of regressing lesions and the X-ray appearances of healing bone lesions are described.

### *Skin recurrences.*

In general skin recurrences show little change during testosterone therapy. This is true of recurrences in mastectomy scars, of lymphatic outspreads, and of distant deposits on trunk, scalp, face or limbs. However, in 2 out of 20 cases (not including those with ulceration) all skin recurrences regressed completely (Cases 20 and 37). In both of these cases, however, the growths were exceptionally sensitive to the hormone, and every other manifestation of disease regressed in as striking a manner as those in the skin (Appendix).

---

EXPLANATION OF PLATE.

Fig. 1.—*Case* 25. Female, aged 39. X-ray photograph of chest 1 week after subcutaneous implantation of 1000 mg. testosterone, showing left pleural effusion and bilateral parenchymatous opacities due to secondary deposits.

Fig. 2.—*Case* 25. Six months after implantation of 1000 mg. testosterone showing clear lung fields and no effusion.

Fig. 3.—*Case* 44. Aged 48. One week before commencement of methyltestosterone therapy, 50 mg. daily sublingually. Confluent ulcerating recurrence in flaps of mastectomy scar.

Fig. 4.—*Case* 44. Four months later. Healed lesion.

1

2

3

4

Of the 18 other patients with apparently similar deposits in skin, only 2 showed any regression at all, and in both it was partial in extent and of short duration.

*Ulcerated lesions.*

Eight patients had recurrent skin lesions on the chest wall, with more or less extensive ulceration. The ulceration was usually the result of necrosis in extensive plaques of growth formed by coalescence of multiple enlarging nodules scattered over the chest wall in the skin flaps on either side of the mastectomy scars, but in 3 cases ulceration had occurred in isolated large nodules of growth. In these three cases marked degrees of healing followed testosterone therapy, but in the more extensive lesions healing was complete in only one case (Case 44). The most important single factor delaying healing seemed to be the depth to which the underlying plaque of growth penetrated, and no substantial healing could be expected in those lesions which involved the thickness of the chest wall and had reached the pleura and lung, as had happened in Cases 31, 34 and 56.

The events when a malignant ulcer heals follow a very characteristic sequence, which was observed most completely in Cases 21, 37 and 44. In each case the pre-treatment appearance of the lesions was similar, namely coarsely granular, raised hard edges with more or less purulent slough and crusted exudate in the irregular necrotic granular floor. The first stage of healing is marked by a reduction in the coarseness of the granular nodules of growth, so that the entire lesion takes on a more smooth and uniform appearance. The quantity of exudate and necrotic material becomes less, and the granular appearance becomes finer and more uniform, giving way finally to a smooth glossy surface. At this stage the formerly raised edges are more or less flush with the surrounding skin, and the sharp line of demarcation is replaced by an imperceptible gradation between skin and ulcer floor as the opalescent epithelium grows over the floor from the periphery, following leashes of easily visible fine blood vessels roughly orientated at right angles to the advancing sheet of epithelium. Pieces cut from the edge for histological examination at this stage show much dermal fibrosis, often with hyaline change ; in Case 44 no viable tumour cells were found, but clumps of malignant cells, some in mitosis, were present among whorls of collagen in the sections from Case 37, but the lesion subsequently healed completely, and has not again broken down 18 months later although fresh cutaneous and osseous metastases have appeared. In Case 44 the ulcerated lesion occupied most of the chest wall, but healing took place in 6 months when all dressings were discarded (Fig. 3 and 4). Fourteen months later several small nodules have appeared in the upper part of the lesion, and a biopsy specimen from one of these shows carcinoma cells in dense fibrous tissue. The effect of depth of lesion in preventing healing was well seen in Case 64. This was a bun-shaped lesion in the upper end of the scar in a woman of 43. It measured 5 cm. in diameter and 1 cm. high, and was firmly fixed to the underlying chest wall. For 3 months after the commencement of testosterone therapy the lesion showed the early stages in the healing process described above. The centre of the lesion, however, became necrotic and infected, and 1 month later no further healing had occurred. The whole area, together with a portion of the underlying rib and pleura, was excised, and examination of the operation specimen showed unusually dense masses of fibrous tissue with very few tumour cells. There is no sign of recurrence 6 months later.

*Localized bony swellings.*

In Cases 4, 17 and 37 localized bony swellings were present before treatment. In Cases 4 and 37 the medial end of the right clavicle was expanded by an exquisitely tender uniform swelling, 4 cm. × 6 cm. in Case 4, and somewhat smaller in Case 37. In Case 17 a hemispherical swelling over the manubrium sterni was present, 2 cm. in diameter. During treatment with testosterone these bony swellings decreased in size, regressing completely in Cases 17 and 37, but coming to a standstill in Case 4, in whom evidence of intrathoracic metastases appeared 5 months later, although the shoulder pains did not recur, and the clavicular swelling was not again tender to percussion. In Case 37 a fresh hemispherical bony swelling appeared 1 year after treatment was commenced over the lateral third of the same clavicle, but the originally present swelling at the medial end has not recurred 20 months later.

*Lymph nodes.*

Like skin lesions, infiltrated lymph nodes rarely regress with testosterone therapy. In the present series lymph nodes were involved in 30 cases (not counting those treated previously by radiotherapy). Of these, complete disappearance of the enlarged nodes was observed in only one patient, Case 20, who has been mentioned above in connection with regression of skin deposits. In her case the growth seemed particularly sensitive to testosterone, and the complete disappearance in 10 weeks of a large hard node 4 × 4 cm. in diameter and filling the angle between the clavicle and the rat-tailed tendon of sterno-mastoid was very striking. This patient relapsed 11 months later. Case 3, a woman of 37, crippled with multiple bone deposits, who obtained complete relief of pain and regained full mobility of neck and limbs after one month's treatment with methyltestosterone, 100 mg. daily for 8 days, followed by 50 mg. daily thereafter, also showed marked regression of axillary and supraclavicular lymph nodes, though some of these were still just palpable 4 months later.

Case 35 was a Stage IV growth in a woman of 36 years. She had nodes in both axillae. One year previously she had been given radiotherapy to the primary growth and to the axilla, and 6 months previously had been given pelvic irradiation for induction of the menopause. Five months later she was given methyltestosterone sublingually 80 mg. daily for 6 months, during which the primary growth and homolateral and contralateral axillary nodes underwent very considerable regression. The nodes in the opposite axilla disappeared completely, so that the patient had become a "Stage II" case, and was therefore submitted to radical mastectomy. The operation specimen revealed a small scirrhous growth, and one axillary node was infiltrated by carcinoma. Six months after the operation nodes again appeared in the opposite axilla and were treated by irradiation. In 3 other cases temporary softening and some diminution in size of enlarged nodes was observed.

The incidence of regression in the several sites of involvement discussed is indicated in Table VII.

TABLE VII.—*Response to Lesions Involving Different Sites.*

| Site of lesions. | Regression. | Partial regression. | Failure. | Total. |
|---|---|---|---|---|
| Skeletal | 8 | 7 | 13 | 28 |
| Intrathoracic | 2 | 3 | 7 | 12 |
| Intracranial | 0 | 0 | 2 | 2 |
| Skin | 4 | 5 | 19 | 28 |
| Chest wall ulceration | 1 | 3 | 4 | 8 |
| Lymph nodes | 2 | 3 | 25 | 30 |
| Stage IV cases | 2 | 0 | 13 | 15 |

### *Some Features of Cases with Bone Deposits.*

*Distribution.*

In this series skeletal deposits were mostly multiple, and were associated with pain in 25 out of 31 cases. In 23 of these cases the spine was involved, the dorsal vertebrae being most often affected, the cervicals least often. There were 19 cases with pelvic involvement, and all of these had spinal deposits as well. In 12 cases with extensive pelvic deposits the femora were also involved, bilaterally 10 times, with destruction of most of the shafts in 1 case, in which pathological fracture of the shaft occurred 2 months after implantation of testosterone. Deposits in ribs, clavicles and scapulae have been recorded 14, 5 and 4 times respectively, but may well have been present in other cases. Five cases had erosion of the sternum with tender bony swelling. Precise data are not available with regard to metastases in the skull, but these were known to be present in 5 cases. The results in cases with skeletal involvement are summarized in Table VIII.

TABLE VIII.—*Cases with Skeletal Involvement. Response to Androgen Therapy in Relation to Menopause and to General Clinical Condition before Treatment.*

| | I. Good condition. | | | | II. Poor condition. | | | | III. Fair condition. | | | | Total. |
|---|---|---|---|---|---|---|---|---|---|---|---|---|---|
| | Relation to menopause. | | | Total. | Relation to menopause. | | | Total. | Relation to menopause. | | | Total. | |
| | Pre- | Artificial meno-pause. | Post- (natural). | | Pre- | Artificial meno-pause. | Post- (natural). | | Pre- | Artificial meno-pause. | Post- (natural). | | |
| uccess | 1 | 1 | 2 | 4 | 2 | 0 | 0 | 2 | 1 | 0 | 0 | 1 | 7 |
| oderate success | 2 | 1 | 1 | 4 | 0 | 0 | 3 | 3 | 1 | 0 | 1 | 2 | 9 |
| ailure | .. | 1 | 3 | 4 | 1 | 2 | 5 | 8 | 0 | 0 | 0 | 0 | 12 |
| adiotherapy as well | 2 | .. | .. | 2 | .. | .. | 1 | 1 | .. | .. | .. | .. | 3 |
| Total | 5 | 3 | 6 | 14 | 3 | 2 | 9 | 14 | 2 | 0 | 1 | 3 | 31 |

*Premenopausal cases with bone deposits.*

Of the 31 cases, 3 received palliative radiotherapy to the affected bones (Cases 2, 27, 69) and are not included in the figures. Of the remainder, 8 were premenopausal cases, all but 2 of whom received great benefit from androgen therapy. Three of these were the bedridden patients referred to above, who were able to resume their normal activities within a few weeks of being treated with testosterone, and remained in good health for over 1 year in 2 cases (Cases 3, 7, 37). The fourth was a housewife of 45 with extensive spinal, costal and pelvic deposits, whose activities were drastically curtailed by pain. She also secured complete relief from pain in the month after methyltestosterone therapy was begun and is well 6 months later (Case 26). Case 4 was a nulliparous woman of 49 complaining of pain in the left hip and right shoulder, the latter exacerbated by the smallest movements of the arm, active or passive. No deposits were seen radiologically to account for the hip pain, but the medial end of the right clavicle was swollen and tender, expanded by a tumour measuring 4 × 6 cm. After taking methyltestosterone 50 mg. daily for one month, the complete range of shoulder movements was restored, the patient was without pain, and the bony swelling no longer tender to percussion, this latter finding being a characteristic result of the treatment. Six months later the swelling is slightly smaller, but intrathoracic metastases have appeared and the general condition has deteriorated. Of the 3 remaining premenopausal cases, 1 (Case 47), a para-2 aged 38, attended hospital 9 months after radical mastectomy with severe anaemia and pains in back, hips and shoulders. She was intolerant to doses of methyltestosterone greater than 30 mg., and although the dose was increased in 5 mg. steps from an initial dose of 10 mg. on 3 occasions the treatment had to be abandoned each time, owing to the sudden onset of headache, nausea, fever, drowsiness and increased intensity of the pains. These symptoms subsided within a day or two on withholding the hormone. Case 66, who presented with paraplegia of 6 weeks' duration, has been referred to above. The last premenopausal case (Case 10), a para-1 aged 45, had had a right radical mastectomy 2 years previously for a lump in the breast present for 1 year. She attended hospital with cough, dyspnoea, pain in the chest and stiff neck. No X-ray evidence of cervical spine involvement was present at the time, but all the symptoms improved after implantation of 500 mg. testosterone. The improvement, however, was short-lived, and 2 months later the pains in the neck returned, and on this occasion radiography revealed extensive destruction of the axis vertebra. Further treatment with methyltestosterone (80 mg. daily) was without value, but the pain quickly responded to local radiotherapy.

*Postmenopausal cases with bone deposits.*

In contrast to the premenopausal patients in whom testosterone conferred marked benefit in 6 out of 8 cases, only 2 out of 15 patients with skeletal metastases who had passed the menopause responded favourably, although pain was relieved for 2 to 3 months in 5 others. Apart from the postmenopausal state and the slightly greater average age, there was little clinically to distinguish these patients from those who improved so greatly after treatment. The preparations and doses used were similar to those which had been given with success to the premenopausal patients. Both of the postmenopausal patients who were benefited by testosterone were in Group I (good general condition), whereas 2 of the most successfully treated premenopausal patients were in very poor condition before treatment (Cases 3 and 37), and a third (Case 26) was assessed as " fair " (Group III). Four of the 7 failures among the postmenopausal cases were in poor general condition before treatment, and accordingly placed in Group II (Cases 12, 49, 51, 67) ; three were in good general condition.

The two " successes " were Cases 14 and 52. The former was a woman of 60 who had had a right radical mastectomy $2\frac{1}{2}$ years before. She complained of increasingly severe " rheumatism " in the right hip, and was found to have a large area of rarefaction in the head, neck and intertrochanteric region of the right femur. She also had 2 large hard fixed parasternal swellings, probably arising in submammary lymph nodes. She was given 100 mg. methyltestosterone daily, and in 3 months' time was walking well and without pain. In addition the parasternal swelling had become greatly reduced in size. Surprisingly she showed on examination 3·5 cm. of shortening of the right femur, but no impairment of movement about the hip-joint, within the limits imposed by the rigidity of her paralysis agitans, and the X-ray revealed an unusual subcapital fracture of the neck with downward and backward displacement of the capital fragment. The second case was of a nullipara aged 51, who had been recurrence-free for 10 years after a radical mastectomy, when she developed backache, which persisted, increased in severity, eventually confining her to bed. Multiple deposits of secondary carcinoma filled most of the dorsal and lumbar vertebrae, and all the pelvic bones. Within a few days of the implantation of 500 mg. testosterone the pain abated, walking was quickly resumed, and she is pain-free 14 months later without further treatment. The nature of the pain is therefore open to question, but the patient gave no history of previous chronic backache, the onset of the pain coincided with the finding of multiple secondary deposits in the appropriate bones, and relief promptly followed implantation of a very small dose of testosterone.

Testosterone was also given to 5 young patients in whom an artificial menopause had been earlier induced (Cases 5, 30, 50, 62 and 70). In the first and last of these cases pain was relieved, but in the first it was never severe, and in the last its recurrence followed the appearance of further metastases.

The difference in response by premenopausal and postmenopausal cases just mentioned was not met with in the series of 70 cases described by Adair, Mellors, Farrow, Woodard, Escher and Urban (1949), or in the larger series of 285 cases analysed by the Council on Pharmacy and Chemistry (1949). In Adair's series 9 out of 48 cases with skeletal metastases benefited from androgen therapy, but no difference in response was noted in premenopausal and postmenopausal cases (12 of the 48 cases were premenopausal, compared with 8 out of 28 in the present series). In the Council's series of 285 cases, the ages ranging from 25–79 years, favourable effects were evidently independent of age. No explanation is offered for this discrepancy.

*Post-treatment changes in bone.*

It is difficult to account for the rapid relief of skeletal pain brought about by testosterone. Equally striking is the speedy disappearance of tenderness to percussion of affected bones, and the later diminution in size of bony swellings due to subperiosteal deposits. Radiologically all that can be observed are the changes in position and concentration of minerals in the neighbourhood of a deposit following testosterone administration, but these changes are characteristic. They consist in the homogeneous deposition of minerals in the bone surrounding the deposit, so that at first the deposits acquire a more definite outline, and may appear more extensive in size and number. This is a common appearance 2 months after commencing treatment, but soon gives way to a more uniform but rather structureless appearance, as the areas of decalcification in the deposits become filled in from the periphery with deposited minerals. After 6 months this process usually comes to a standstill. It is questionable whether this recalcification confers increased strength on the bones in which it takes place, for further collapse of vertebral bodies may occur while it is in progress, and in Case 14 early remineralization was occurring at the time of the fracture of the femoral neck referred to above. The illustration of one case in the paper of Hermann, Adair and Woodard (1947) also shows a pathological fracture of the shaft of the humerus in which much sclerosis had taken place. It may in this case have assisted the impaction which " cured " the fracture. Good examples of recalcification in this series are seen in Cases 3, 5, 7, 26 and 37, and in Cases 3 and 60 collapse of more vertebral bodies occurred during the process. Perhaps immobilization during the period of remineralization would prevent such further collapse, weight-bearing being avoided until the process was complete.

Androgen therapy does not of course prevent the development of fresh blood-borne metastases in bone or other tissues. Several authors have noted cases originally free of skeletal deposits, in which these appeared during treatment (Cutler and Schlemenson, 1948 ; Hermann, Adair and Woodard, 1947) ; Adair, Mellors, Farrow, Woodard, Escher and Urban, 1949). The same is true of cases with bone deposits, and in the illustrations in the first two papers just cited fresh osteolytic deposits are present in bones showing extensive recalcification round earlier deposits. Several instances of this phenomenon have been met with in the present series. The paradox is probably to be explained by the fact that the metabolic processes accelerated by the androgen proceed most rapidly at the beginning of treatment, and then slow down to a stationary though raised level. Bony metastases developing some time after treatment has begun are therefore no longer exposed to the same biochemical conditions as those present at the commencement of treatment.

### DISCUSSION.

From the therapeutic standpoint the effects of androgen administration resemble but are not superior to those of castration. Dramatic improvement occurs with both methods in a small percentage of cases, and in larger numbers less striking remissions are seen, but most cases do not benefit. In the most favourable cases remissions last for 5 years or more, but in the remainder, recurrence and deterioration in health appear between 6 and 12 months from the start of treatment. With neither method can a favourable result be foreseen ; probably more successes are met with in the premenopausal group, although striking improvements often occur in women who have passed the menopause, sometimes many years previously.

### *The Results of Surgical Castration for Mammary Carcinoma.*

#### *Early work.*

Schinzinger (1889) drew attention to the rapid course of the disease in young women as compared with that in elderly women, and suggested that castration in the former might be expected to improve the prognosis by artificially hastening the onset of old age. He does not seem to have put his idea into practice. The young science of bacteriology was advancing rapidly at that time, and it was hoped that infective agents would in time be found as specific causes of most of the major diseases. Several papers appeared at the turn of the century describing intracellular bodies in association with cancer, which were thought to be parasitic yeasts. In opposition to the parasitic theory of carcinogenesis, Beatson (1896), of Glasgow, published an account of two cases of mammary carcinoma in which striking improvement had followed castration. Beatson believed that carcinoma was the expression of an abnormal kind of lactation in which the mammary epithelium pushed its way outwards into the stroma instead of inwards towards the lumen, there to disintegrate in the form of milk. He had learned of " the custom in certain countries to remove the ovaries of the cow after calving if it is wished to keep up the supply of milk, and that if this is done the cow will go on giving milk indefinitely," and this " pointed to one organ holding the control over the secretion of another and separate organ, and thus explained the absence of that distinct nervous control that I pointed out as characteristic of the mamma." Beatson's first case will be described in some detail, as it illustrates very well the kind of result that may be obtained by testosterone therapy as well as by castration. The patient was a married woman of 33 years, with 2 children, in fair general health, and menstruating regularly. Three years previously she had noticed a lump in the breast while suckling her first child. This increased in size after the birth of her second child 20 months later. When first seen there was a mass $5 \times 3\frac{1}{2}$ inches with ulceration of the skin of the breast over an area of 1 square inch and adjacent skin nodules. Radical mastectomy was performed on 25.i.95, but 3 months later an ulcerated recurrence in the scar was found, and a fortnight later had increased in size to $2\frac{1}{2} \times 3\frac{1}{2}$ inches. The whole chest wall was studded with skin nodules, but no lymph nodes were felt. On 15.vi.95 both ovaries were removed ; the left was " cystic," the right normal. By 19.vii.95 the " larger mass was much less vascular, smaller, flatter and altogether less prominent, and the same may be said of all the other secondary foci of disease. The tissues around were also softer and more pliable." By 1.viii.95 the " largest area of disease " measured $1\frac{1}{4} \times 2\frac{3}{4}$ inches. Eight months later regression continued. The patient lived until April, 1899, dying 46 months after castration, with fresh recurrences in skin and spine (Thomson, 1902). The second case was that of a woman aged 40, with massive involvement of the whole breast by carcinoma, with extensive infiltration of skin, axillary and cervical nodes as far as the chin ; the opposite cervical nodes were also involved, and she complained of pain in neck and arm. After castration the pain was relieved, and regression and softening of the primary mass and of the nodes occurred, lasting 5 months.

Thus in the first 2 cases in which castration was used, shrinkage of the primary growth, regression of skin deposits and of involved nodes, general improvement, and relief of pain were described. Six years later the method had been used extensively, and its value summed up by Beatson (Morris, 1902) as follows : " It is not easy to say beforehand if the operation will do good ; but from the cases recently published by Dr. Abbe, of New York (Thomson, 1902 ; Abbe, 1901), it would seem that it is not contra-indicated after the menopause . . . the value of an operation may be judged by statistics and by personal impressions. Both may be deceptive, though both may contain in them the germs of truth. I believe that from both standpoints the evidence is in favour of the utility of oöphorectomy in certain cases, in prolonging life and lessening pain. It has never, as far as I know, done any harm, and its mortality has been *nil* . . . the treatment has been placed before the profession, because I felt that they should judge its effects themselves ; and if, in their opinion, after a fair trial, it is found wanting, then I hope it will be abandoned and forgotten." Oöphorectomy passed out of fashion a few years later, and was slowly superseded by " X-ray castration," and in the last 10 years by " medical castration " with androgens also.

Boyd (1900) reported the results of oöphorectomy in 54 cases of mammary cancer. Six cases out of 46 showed remissions lasting for 2 years or more, and 17 out of 46 derived some benefit, but in most of these, recurrences occurred between 6 and 12 months later. With one exception the treatment failed in all the patients who had passed the menopause, and the maximum benefit seemed to be derived by women over 40 and still menstruating. Two years later Thomson (1902) of Edinburgh collected and published the results in 80 inoperable cases castrated by several surgeons. In 40 of the cases some improvement took place, although this was negligible in 11, and only temporary in 11 others (under 12 months' survival following oöphorectomy). In 18, however, decided improvement was recorded, " remarkable " in 4. Thirteen cases survived more than 20 months, and 4 between 40 and 46 months. Of the 29 more favourable cases, 6 had ceased menstruating, and 3 were 70 years of age, and of the 20 women still menstruating, 12 were between 40 and 50 years of age. Thomson concluded that the value of castration, though limited, had been established beyond doubt, but, like Beatson (1896), stressed the unpredictability of the results. He drew attention to the cases in which marked improvement had occurred after the menopause, and although he noted the frequent relief of pain and general improvement following the operation, he considered it to be contra-indicated when metastases were present in the viscera or bones. However, in only 6 of the 80 cases listed is skeletal disease mentioned, and 2 of these were in the group in which the best results were obtained. The limited use and poor quality of the diagnostic radiography of the time invalidates the figures relating to bone metastasis. Thomson also considered that the use of thyroid extract, which Beatson had earlier thought to be an important part of the treatment, was without value.

Early experience of castration at the Cancer Hospital, Brompton, was not so favourable as that from other hospitals. Thus F. B. Jessett " had operated on 5 cases, and admitted that the patients complained of less pain, but in no single case was there any improvement in the local disease, which ran its ordinary course, and the patients died. He had used the X-rays in a few cases . . . but regretted his experience was not that of other speakers, who had recorded such admirable results " (Morris, 1902). Herbert Snow had no better fortune : " some 10 or 12 oöphorectomies had been performed, but never with any useful result ; the X-rays had been used for some 20 miscellaneous cases with no better consequence " (Morris, 1902). " The X-rays " mentioned by these surgeons referred to local irradiation of disease, not to pelvic irradiation.

Lett (1905) reported the results of castration in 99 cases of mammary carcinoma in 1905. Of these 23·2 per cent derived marked benefit, and if the patients over 50 years of age were excluded, the percentage of successes rose to 41·3 per cent. The nature of the improvement was similar to that described by previous writers. " In successful cases the benefit has been great, and was mainly shown in relief from pain, marked improvement in health, diminution or even disappearance of the growth, healing of ulcers and prolongation of life." One of Lett's patients remained alive and well for 5 years after castration, in 15 improvement was maintained for more than 12 months, and 4 others were in good health $4\frac{1}{2}$ years or more afterwards. He also noted that beneficial results occurred sometimes after the menopause.

Bruce Clarke recorded a case in which castration was followed within 6 weeks by rapid subsidence of pain and regression of a chest wall recurrence, the patient being in good health 5 years later. In this case unfortunately the age was not stated, but the original excision of the growth was performed 2 years before castration, and a recurrence excised 1 year before (Lett, 1905).

In spite of the well-documented reports described above, castration for mammary cancer fell out of favour, no doubt because of the absence of precise indications, the large number of poor results and the increasing success obtained by local radiotherapy. Morris (1902) stressed the difficulty of assessing the results, and compared " Dr. Beatson's treatment " with the favourable results claimed for the injection of Coley's fluid (heat killed broth cultures of streptococci obtained from erysipelas lesions, and of *Bacillus prodigiosus*) in cases of spindle-celled sarcoma. He also felt that Beatson's view of the ovarian control over mammary cancer was incompatible with the frequent onset of the disease in elderly women, in whom the ovaries were presumed to be inactive. The difficulty of assessing the value of castration was also pointed out by Michels (1905), and thereafter interest waned, although sporadic reports continued to appear (Torek, 1914).

*Recent work.*

In recent years attention has again been directed to surgical castration. Herrell (1937) reviewed the records of 1906 women treated for mammary cancer, and of 1011 women in a similar age-group without mammary cancer. He found that in the cancer-bearing group the incidence of complete oöphorectomy before carcinoma was diagnosed was 1·5 per cent, whereas in the non-cancer group it was 15·4 per cent, or ten times as great. These figures implied that castration might have protected a small number of women from the subsequent development of mammary carcinoma.

Arguing on these lines Horsley, late in 1937, performed bilateral oöphorectomy on the first of a series of patients immediately after radical mastectomy, in the hope of deferring the onset of recurrence. By June, 1944, recurrences had occurred in only 4 out of the 25 patients who had been observed for between 9 months and nearly 7 years. Horsley felt that the appearance of metastases had been delayed by castration (Horsley, 1944, 1945). Snapper castrated 22 patients after radical mastectomy, and in addition treated them for 3 months with injections of testosterone propionate 25 mg. thrice weekly, and intermittent courses subsequently. Fifteen of these patients had involved axillary lymph nodes at the time of radical operation. Between 12 and 27 months later 4 had already developed recurrences, all 4 belonging to the group with initial axillary involvement (Stage II cases). Snapper concluded that no significant protection had been conferred by the method (Snapper, 1947).

Ulrich carried out hysterectomy and bilateral oöphorectomy in a woman of 42 with metrorrhagia from uterine fibroids and with bilateral mammary carcinoma with skin infiltration and axillary node involvement. The patient also received several short courses of testosterone acetate (40 mg. daily for 5 days). The local pain associated with the right breast disappeared after the first course of injections, and some decrease in size of the growths with increased mobility and lessened skin infiltration followed (Ulrich, 1939*a*).

Farrow and Adair (1942) reported a case of mammary cancer in a man of 72, in which marked improvement followed castration. Partial regression of the ulcerated primary growth occurred, there was complete relief from bone pain within 2 weeks, and increased density was described in the osteolytic deposits in ribs, vertebrae and scapula, accompanied by slight elevation of serum alkaline phosphatase. Castration for carcinoma in males was not described by the earlier British workers, though Beatson (1896) felt that the testis exerted the same distant influence on other organs as the ovary. Adair, Treves, Farrow and Schnagel (1945) summarized the results of castration by surgery and by X-rays in 335 females, and in a small number of males. Of 304 women in whom artificial menopause was induced by X-irradiation, 47 (15 per cent) were improved, and 4 out of 31 women surgically castrated were improved. The figures are not quite comparable with those of Beatson (1896), Boyd (1900), Thomson (1902), and Lett (1905), as only premenopausal cases were included, and no patient was over 55 years of age. Of the 51 improved cases in the whole series, 33 were 40 years of age or less. Of the male cases the results of surgical castration were considered to be better in older men, and " as dramatic as in cases of prostatic carcinoma." The patient mentioned above (Farrow and Adair, 1942) remained well for 2½ years. Boger (1946) described the case of a married woman of 27, crippled with pain from skeletal deposits of mammary cancer, in whom relief of pain was obvious within 48 hours of castration. In this case 30 mg. of methyl-testosterone was given on the day after oöphorectomy, but in view of subsequent experience with this hormone, it is probable that the immediate effect was due to the oöphorectomy alone.

A case of intrathoracic recurrence in a woman of 40 years, 2 years after radical mastectomy for Stage II carcinoma, in whom relief of dyspnoea was observed 4 days after castration, and during the next 2 months absorption of malignant pleural effusion and resolution of pulmonary parenchymal deposits took place, was reported by Jochweds, Baranowicz and Horecki (1948).

The effects of surgical castration may be summarized by saying that it confers definite benefit on a small percentage of cases of carcinoma of the breast, even in an advanced stage, as shown by relief of severe bone pain, recalcification of areas of destroyed bone, regression of primary and secondary manifestations of disease, and conspicuous improvement in general health lasting usually for several months only, but sometimes for several years. The most favourable cases are those in the premenopausal group in women, though striking improvement occasionally follows castration in older women, and in the small number of cases reported in males the best results are to be expected in elderly patients.

In passing it should be noted that some of the remarkable spontaneous regressions which occur, and which may resemble in all respects those already described, are probably the result of " self-castration," both ovaries being completely destroyed by metastases. One such case, in a young woman with widespread recurrences in bones and skin, in whom rapid deterioration was suddenly arrested and followed by 18 months of sustained improvement, was described by Adair, Treves, Farrow and Schnagel (1945).

*The results of X-irradiation of the ovaries in mammary carcinoma.*

Irradiation of the ovaries as a substitute for surgical castration was first used by de Courmelles (1909, 1926), whose first report appeared in 1909, the method having been used since 1904. He considered the results superior to those obtained by surgery. Wintz began to practise ovarian irradiation in 1920, and described his results 6 years later (Wintz, 1926). Ahlbom (1930) attempted a statistical analysis of his cases, but as 147 out of 163 cases received treatment to other sites as well as to the pelvis, and his series included very few instances of women in the most favourable age group, he failed to demonstrate any positive effects. Very few clinical data are presented in Ahlbom's paper, and in spite of his obvious anxiety to attain statistical precision, it is perhaps surprising that he could quote no examples of undoubted success similar to those reported so often in cases subjected to surgical castration. Clarkson and Barker (1936) reported a case of mammary carcinoma in a woman of 41 with axillary node involvement, and deposits in a metacarpal, a tarsal phalanx, femur and ilium, in which recalcification of destroyed areas in the skeleton followed ovarian irradiation and general body irradiation. The patient was in good health 5 years later ; the ovarian irradiation was considered to be the main cause of the good result.

Dresser (1936) reported the results of ovarian irradiation in 59 cases with skeletal metastases, having selected this group for special observation because of the dramatic benefit conferred on two women with generalized skeletal and other deposits after they had received small doses (700 r) of X-rays directed to the pelvis for local relief of pain. In both cases the periods ceased, general regression of deposits occurred, with skeletal recalcification, and in the second case multiple pulmonary deposits disappeared ; the patient led an active life for another 3 years, largely symptom-free. Of Dresser's 59 cases, 30 were under 45 years of age, and 29 had passed the menopause. None of the post-menopausal patients derived benefit from irradiation of the ovaries. Temporary pain relief occurred in 13 of the patients under 45, but no regression was observed, but in 9 of these patients the relief of pain was accompanied by marked skeletal recalcification and improvement in general condition lasting from 2–3 years in several instances.

Between 1938 and 1945 Halberstaedter and Hochman carried out ovarian irradiation on 60 patients with mammary carcinoma, most of whom were advanced recurrent cases. Of these patients 34 derived some benefit, not necessarily with objective evidence of regression, lasting from 6 months to 2 years (Halberstaedter and Hochman, 1946).

Reviewing the position Taylor (1938) concluded that there was " a small sub-group of young women . . . in whom artificial menopause has shown striking benefit," and suggested that the ovarian hormones were necessary for the growth of mammary carcinoma.

*Selective regression of primary growths and metastases.*

Although cases are occasionally seen in which every accessible deposit of growth regresses completely after surgical or X-ray induced castration or after administration of hormones, the commoner occurrence is for some deposits to undergo more marked regression than others. This was noted by the early observers. Thus Morris (1902) wrote: "cancerous nodules in the same tissue lying side by side are not uniformly affected by Beatson's treatment; lymphatic glands yield less readily than cutaneous or subcutaneous nodules, and Stanley Boyd thinks the supraclavicular glands yield more slowly than the axillary." In the case described by Archer and Cooper (1940), X-ray induced menopause was followed by regression of the primary growth and of the supraclavicular mass, but the axillary mass and pulmonary metastasis remained unchanged. The present tendency is to regard skeletal metastases as the most sensitive to irradiation castration. This is exemplified by Dresser's (1936) series, by the cases reported by Farrow and Adair (1942), Adair, Treves, Farrow and Schnagel (1945), Boger (1946), Hunt (1940), Clarkson and Barker (1936), and Ritvo and Peterson (1944). The last-named authors thought that one-third of selected patients with osseous metastases might be expected to improve, and they do not recommend the treatment for other groups. However, relief of respiratory symptoms and regression of pulmonary deposits have been reported many times (Adair, Treves, Farrow and Schnagel, 1945; Jochweds, Baranowicz and Horecki, 1948; Dresser, 1936; Hunt, 1940; Taylor, Slaughter, Smejkal, Fowler and Preston, 1948).

The results of X-ray induced castration may therefore be summarized by saying that in general they resemble those obtained by the surgical method, both with regard to the percentage of successes, the group of cases in which benefit is to be expected, and the nature and duration of the improvement. The discrepancy between the results of the early surgical cases, in which castration was believed to be contra-indicated in the presence of bone metastasis, and the more recent results in which skeletal metastasis is regarded as one of the most favourable indications for ovarian irradiation, is probably adequately explained by the lack of satisfactory X-rays in the early work, so that the extent and incidence of bone metastasis was less accurately known.

*The effects of androgens in mammary carcinoma.*

Among the first reports of the use of testosterone for mammary carcinoma were those of Ulrich (1939*a*, *b*) and of Loeser (1941). The latter used intramuscular injections and also subcutaneous implants of testosterone propionate. The first 3 cases were recurrence free at the time treatment was begun, but remained so for the next 3 to 4 years, whereas all had been treated by X-rays or surgery for recurrences shortly after the original operation (6 months, 8 months and 2 years respectively). Farrow and Woodard (1942) felt that there was a specially close relation between susceptibility to skeletal metastasis and ovarian activity, and were thus led to use testosterone for cases with bone deposits. They analysed the distribution of skeletal metastases in a group of 1380 cases of mammary carcinoma, and commented on the tendency to develop bone deposits early in the course of the disease in the premenopausal group, and late in the post-menopausal group, so that in the distribution curve there were two peaks of high incidence of skeletal deposits, one in the years preceding the menopause, and a trough of low incidence during the menopausal years.

Their earlier results with testosterone were, however, discouraging. In 3 cases administration of the hormone was rapidly followed by headache, nausea and vomiting, depression, flushing and persistence or even exacerbation of bone pain.

X-rays showed rapid extension of the bone disease. These occurrences were correlated with rapid skeletal decalcification with elevation of serum calcium level, and the authors felt that the hormone should not be used in cases of skeletal metastasis. In subsequent years the same workers reported much better results when larger doses of testosterone propionate were used, and were inclined to attribute their earlier lack of success to inadequate dosage (Adair, 1947). Nevertheless good results have been reported with doses of the same order as were originally employed by Farrow and Woodard, for example, by Fels (1944), and by Schwander and Marvin (1947). Bone metastases were present in the cases of Fels and of Schwander and Marvin, and the most impressive result of the treatment in one of Fels' cases was that the patient, a woman of 34, was able to leave her bed for the first time for 18 months, having received 700 mg. of testosterone propionate in 25 mg. doses given on alternate days.

The case reports published by Adair and Herrmann (1946) illustrate very well the kind of result which may follow the use of testosterone therapy. Weight gains, general improvement, rapid relief of pain previously intractable, regression of primary growths and secondary deposits, and increased calcification of bony deposits were recorded. The following are especially noteworthy :

(1) *The ages of the patients.*—One of the best responses occurred in a woman of 63 years, though the other successes were in women of 47, 44 and 42 years of age. Compare also Taylor, Slaughter, Smejkal, Fowler and Preston (1948), who report and illustrate regression of pulmonary deposits in a woman of 70, and the progress report of the American Council on Pharmacy and Chemistry (Council on Pharmacy and Chemistry, 1949), in which the response to androgens in 285 patients ranging from 25–79 years was evidently independent of age.

In a later paper Adair (1947) reports a dramatic result in a woman of 71 who had been confined to bed for 10 months with severe pain and pathological fractures due to widespread metastases in spine, pelvis and ribs. Three weeks after commencing testosterone therapy she remarked on her improved sense of well being, and 4 weeks later left her bed. Two years and 4 months after she was still up and about and in good health, the only untoward effect being irritation from the enlarged clitoris. This case recalls some of those treated by surgical castration (Thomson, 1902).

(2) *The speed of response.*—In Case 4 relief of pain was evident within 1 week of the first injection, and in Case 3 regression in primary and metastatic growths was noted one month later.

(3) *The favourable effect on skeletal metastases.*—The earlier results of Adair (1947) suggested that the best results of androgen therapy were to be expected in cases with metastases in bone. However, equally dramatic results have been obtained in cases with pulmonary deposits and skin deposits, but no secondaries in bone of the common osteolytic type revealed by X-rays. These results are said to be much less common than in cases with bone deposits. Thus Adair stated: " In only a small percentage of cases is improvement to be anticipated where carcinoma involves soft tissues such as liver, lungs, brain and local skin recurrence. It is true that in a few instances there have been striking improvements where an advanced ulcerating breast cancer with surrounding skin nodules, axillary nodes and neck nodes were involved ; where masses were metastatic to the lungs ; and in one instance where the patient was having convulsions from metastatic disease in the brain . . . most such . . . receive no benefit

4

aside from the temporary general improvement which frequently comes as a result of testosterone injections. On the other hand, a most striking improvement is obtained in most of those cases having bone metastasis " (Adair, 1947).

In general the earlier results of Adair and his colleagues have been confirmed by others, but it is now clear that the proportion of cases with skeletal metastases which responds is not higher than that of all types of case taken together, although the remissions obtained in these cases are more often of practical therapeutic value, owing to the great disability so commonly associated with osseous metastases. Moreover, the beneficial effects of androgens are not superior to those of surgical castration, and a common mode of action probably underlies both methods, though it is premature to speculate further on this matter.

Cutler and Schlemenson (1948) observed 19 consecutive cases of advanced mammary carcinoma for not less than one year after treatment with testosterone propionate. All the patients died within 14 months after the commencement of treatment, 11 of them received no benefit from the treatment, including 6 with skeletal deposits, but some undoubted improvement took place in 8 cases, transient in 4, but more substantial in the remaining 4. The case with the longest benefit was that of a woman aged 42 years with multiple bone deposits and dyspnoeic from a right pleural effusion. The dyspnoea was relieved a fortnight after testosterone therapy was begun, the effusion was absorbed and the bone deposits became recalcified. The disease again became active 11 months later. In another case, a woman of 58 years, a large hemispherical scalp deposit regressed, and its site became again covered with hair. These authors are careful to point out that in 3 cases skeletal deposits occurred during testosterone therapy, and are anxious to stress the limitations of the method, although fully recognizing its value. They recommend continuing injections until benefit is no longer conferred. In view of the small proportion of cases in which favourable responses are seen, it is not surprising that many workers who have treated small numbers of cases have failed to demonstrate the specific action of androgens in mammary carcinoma. Thus Luft (1948) and Watson and Fetterman (1949) each treated 7 cases without observing any objective improvement.

In an analysis of 285 patients treated at various centres in the United States, and compiled by the Council on Pharmacy and Chemistry, regressions in soft tissue lesions were found to occur in about 20 per cent of cases, and in skeletal lesions in 18 per cent. The response was apparently independent of age, and no support was given to the earlier trends towards increasing the dosage to higher and higher levels. In 20 per cent of the cases which derived benefit, the improvement lasted for 9 months or more (Council on Pharmacy and Chemistry, 1949). Instances in which patients have led useful lives for 3 years or more are by no means uncommon (Adair, 1947, 1949), even when the disease was in a most advanced stage, but in a majority of cases relapse occurs within 1 year of the commencement of androgen therapy.

In practice the chief limitations of androgen therapy are : (1) the poor response in 80 per cent of cases ; (2) the impossibility of pre-selecting the 20 per cent of successes ; (3) the inevitability of relapse ; (4) the expense of the hormones.

At present nothing can be said about the first 3 points. With regard to the last, the expense of treatment can be minimized by using the implantation method in all cases which have been shown to respond favourably to oral or injection therapy, by discontinuing treatment in every case in which specific response fails to occur within two months, and by avoiding excessive doses.

All but 5 of the cases here reported were too far advanced to warrant any other form of treatment, but there are many cases in whom androgen therapy and radiotherapy might profitably be given simultaneously, and work on these lines is in progress.

### CONCLUSIONS.

Fourteen out of 70 patients with advanced mammary carcinoma (55 recurrent cases, 15 inoperable cases) obtained substantial remissions as a result of androgen therapy.

These remissions permitted resumption of normal activity for more than 6 months in all but one case which is still in remission after 5 months, for more than 1 year in 10 cases, 6 of which are still in good health.

Sixteen other patients obtained some benefit from the treatment, but the regressions were incomplete, and lasted 6 months or less.

Seven out of 31 patients with skeletal metastases obtained complete relief of pain, 4 having been bedridden before treatment. Four are in good health over one year later, 2 between 6 and 12 months, and 1 five months later.

Of the 14 "successes," 5 received implants of testosterone (3 of 500 mg., 2 of 1000 mg.) and 9 received sublingual methyltestosterone (6 of 50 mg. daily, 3 of 50–100 mg. daily). Three patients who originally received implants were later given methyltestosterone, and 2 patients who relapsed were given large doses of intramuscular testosterone propionate, but without benefit. One of them again improved when the injections were discontinued.

Testosterone implantation and sublingual methyltestosterone are considered to be as effective as intramuscular therapy with testosterone propionate. Implantation is recommended in cases responding favourably to a "therapeutic trial" with one of the other methods.

Androgen therapy offers a convenient alternative to surgical castration, but the results are probably no better.

### SUMMARY.

The treatment of 70 cases of advanced mammary carcinoma by means of androgens is described, with special reference to the use of sublingual methyltestosterone and of subcutaneous implants of testosterone.

The literature relating to surgical castration, X-irradiation of the ovaries and androgen therapy in inoperable or advanced recurrent mammary carcinoma is briefly reviewed.

Useful remissions are to be expected in 20 per cent of cases when one or other of these methods is used, but the group cannot be pre-selected by clinical means.

This investigation has been supported by generous grants made to the Royal Cancer Hospital by the British Empire Cancer Campaign, the Jane Coffin Childs Memorial Fund for Medical Research, the Anna Fuller Fund, and the Division of Research Grants of the U.S. Public Health Service.

I wish to express my gratitude to Professor A. Haddow and Professor E. Boyland for their encouragement, advice and criticism, and to the honorary surgical staff for allowing me to treat the patients under their care. I am greatly indebted to Miss A. Greenwood and Miss G. Reghers for help with the manuscript, and to Miss J. Hunt for the photographs.

The testosterone implants were given by Organon Laboratories, Limited, London, W.C.2, through the courtesy of Dr. J. W. Tindall, to whom I am also indebted for much helpful advice.

### REFERENCES.

Abbe, R.—(1901) *Med. Rec. N.Y.*, **60**, 951.

Abels, J. C., Nelson, F., Young, N. F., and Taylor, H. C.—(1944) *J. clin. Endocrinol.*, **4**, 198.

Adair, F. E.—(1947) *Surg. Gynaec. Obstet.*, **84**, 719.—(1949) *Proc. Roy. Soc. Med.*, **42**, 468.

*Idem* and Hermann, J. B.—(1946) *Ann. Surg.*, **123**, 1023.

*Idem*, Mellors, R. C., Farrow, J. H., Woodard, H. Q., Escher, G. O., and Urban, J. A.—(1949) *J. Amer. med. Ass.*, **140**, 1193.

*Idem*, Treves, N., Farrow, J. H., and Schnagel, I. M.—(1945) *Ibid.*, **128**, 161.

Ahlbom, H.—(1930) *Acta Radiol.*, **11**, 614.

Anning, S. T., Dawson, J., Dolby, D. E., and Ingram, J. P.—(1948) *Quart. J. Med.*, **17**, 203.

Archer, V. W., and Cooper, G.—(1940) *Amer. J. Roentgenol.*, **44**, 108.

Beatson, G. T.—(1896) *Lancet*, ii, 104, 162.

Boger, W. P.—(1946) *J. clin. Endocrinol.*, **6**, 88.

Boyd, S.—(1900) *Brit. med. J.*, ii, 1161.

Butenandt, A., and Hanisch, G.—(1935) *Z. physiol. Chem.*, **237**, 89.

Carlinfanti, E., D'Alo, F., and Cutolo, L.—(1949) *Lancet*, i, 479.

Clarkson, W., and Barker, A.—(1936) *Amer. J. Roentgenol.*, **36**, 615.

Council on Pharmacy and Chemistry.—(1949) *J. Amer. med. Ass.*, **140**, 1214.

de Courmelles, F.—(1909) *C.R. Acad. Sci.*, **148**, 1281.—(1926) *Acta Radiol.*, **6**, 322.

Cutler, M., and Schlemenson, M. L.—(1948) *J. Amer. med. Ass.*, **138**, 187.

Dresser, R. –(1936) *Amer. J. Roentgenol.*, **35**, 384.

Farrow, J. H., and Adair, F. E.—(1942) *Science*, **95**, 654.

*Idem* and Woodard, H. Q.—(1942) *J. Amer. med. Ass.*, **118**, 339.

Fels, E.—(1944) *J. clin. Endocrinol.*, **4**, 121.

Foss, G. L.—(1938) *Lancet*, i, 992.

Geist, S. H., Salmon, U. J., and Gaines, J. A.—(1938) *Endocrinol.*, **23**, 784.

*Idem*, Salmon, U. J., and Walter, R. S.—(1940) *J. Amer. med. Ass.*, **114**, 1539.

Haddow, A., Watkinson, J. M., Paterson, E., and Koller, P. C.—(1944) *Brit. med. J.*, ii, 393.

Halberstaedter, L.—(1905) *Berl. klin. Wschr.*, **42**, 64.

*Idem* and Hochman, A.—(1946) *J. Amer. med. Ass.*, **131**, 810.

Hayden, J. R.—(1895) *Med. Rec., N.Y.*, **47**, 612.

Hermann, J. B., Adair, F. E., and Woodard, H. Q.—(1947) *Surgery*, **22**, 101.

Herrell, W. E.—(1937) *Amer. J. Cancer*, **29**, 659.

Horsley, J. S.—(1944) *Surgery*, **15**, 590.—(1945) *J. Amer. Med. Ass.*, **128**, 166.

Huggins, C., and Hodges, C. V.—(1941) *Cancer Res.*, **1**, 293.

Hunt, H. B.—(1940) *Radiology*, **34**, 235.

Jochweds, B., Baranowicz, P., and Horecki, J.—(1948) *Polski Tygodnic Lekarski*, **33**, 992.

Kenyon, A. T., Sandiford, I., Hughes, B. A., Knowlton, K. and Koch, F. C.—(1938) *Endocrinology*, **23**, 135.

Lathrop, A. E. C., and Loeb, L.—(1916) *J. Cancer Res.*, **1**, 1.

Lett, H.—(1905) *Lancet*, i, 227.

Loeser, A. A.—(1941) *Ibid.*, ii, 698.

LUFT, R.—(1948) *Nordisk Med.*, **39**, 1348.
MICHELS, E.—(1905) *Munch. med. Wschr.*, **52**, 1136.
MORRIS, H.—(1902) *Brit. med. J.*, ii, 1161.
PAPANICOLAOU, G. N., RIPLEY, H. S., AND SHORR, E.—(1938) *Proc. Soc. exp. Biol., N.Y.*, **37**, 689.
PATERSON, E., HADDOW, A., APTHOMAS, I., AND WATKINSON, J. M.—(1946) *Lancet*, i, 677.
PRUDENTE, A.—(1945) *Surg. Gynaec. Obstet.*, **80**, 579.
RHOADS, C. P.—(1946) *J. Amer. med. Ass.*, **131**, 656.
RITVO, M., AND PETERSON, O. S.—(1944) *Amer. J. Roentgenol.*, **51**, 220.
ROUG, H. R., AND ZAKON, S. J.—(1943) *Arch. Derm. Syph., N.Y.*, **48**, 601.
SCHINZINGER.—(1889) *Chir. Kongress I*, 28.
SCHWANDER, H., AND MARVIN, H. N.—(1947) *J. clin. Endocrinol.*, **7**, 423.
SNAPPER, I.—(1947) *J. Mt. Sinai Hosp.*, **14**, 618.
TAYLOR, G. W.—(1938) *Amer. J. Roentgenol.*, **39**, 419.
TAYLOR, S. G., SLAUGHTER, D. P., SMEJKAL, W., FOWLER, E. F., AND PRESTON, F. W. —(1948) *Cancer*, **1**, 604.
THOMSON, A.—(1902) *Brit. med. J.*, ii, 1293.
TOREK, F.—(1914) *Ann. Surg.*, **60**, 476.
ULRICH, P.—(1939*a*) *C.R. Soc. franç. Gynéc.*, **9**, 70.—(1939*b*) *Acta Union Int. contre Cancer*, **4**, 377.
WATSON, J. R., AND FETTERMAN, G. H.—(1949) *Surg. Gynaec. Obstet.*, **88**, 702.
WHITE, J. W.—(1893) *Ann. Surg.*, **18**, 152.—(1895) *Ibid.*, **22**, 1.
WINTZ, H.—(1926) *Brit. J. Radiol.*, **31**, 150.
ZUCKERMAN, S.—(1937) *Lancet*, i, 992.

### APPENDIX.

### *Illustrative Case Reports.*

The following summaries of 8 case histories illustrate androgen therapy in the advanced untreated case of mammary carcinoma (Cases 3 and 20 " successes," Case 28 a " failure " subsequently responding to oestrogen), in the recurrent case with skeletal metastasis (Case 37 a " success," Case 12 a " failure "), with intrathoracic metastasis (Case 25 a " success," Case 13 a " failure "), and with confluent chest-wall ulceration (Case 44, a " success ").

*Case* 3.

K. B—, aged 37. One child, 6½ years. Menstruation regular.

*June*, 1947.—First noticed small mobile nodule in left breast.

*June*, 1948.—Reported to private doctor. Referred promptly for radical mastectomy.

*August*, 1948.—Felt stiff down left leg and developed constant pain in small of back.

*October*, 1948.—Pain so severe that she could not walk without support.

*November*, 1948.—Pain worse. Fresh pains across shoulders.

*January*, 1949.—Bedridden.

28.ii.49.—First seen Royal Cancer Hospital ; slightest movement of arms or legs distressed her. Kyphotic telescoped spine with lower ribs splayed over iliac crests. Several small hard nodes right axilla, right supra-clavicular fossa. X-rays : Extensive metastases throughout dorsal spine, with compression fractures of dorsal vertebrae 4, 5, 6, 8, 12 and lumbar vertebra 2. Fourth cervical almost completely destroyed, spreading into lamina and base of spine and to body of 5th cervical. Multiple pelvic deposits ; upper ends of both femora involved.

*Treatment.*—Between October and November, 1948, diathermy, radiant heat, ultra-violet light, massage, infra-red treatments without benefit.

28.ii.49.—Methyltestosterone 50 mg. *b.d.* for 8 days commenced.

7.iii.49.—Dose reduced to 50 mg. daily.

*Progress.*—Pains diminished during first fortnight after taking tab. methyltestosterone, and after 1 month she was pain-free and able to walk short distances without aid. Gained 7 lb. in first 6 weeks. By 27–vi.49 was leading an active normal life, her only complaint being of recurrent crops of pimples on face, chest and shoulders. Amenorrhoeic. She walked normally, showed full range of movements of head, neck and limbs. Kyphosis of spine more exaggerated with gibbus over upper dorsal spine. Soft nodes only in axilla and right supraclavicular fossa. Slight facial hirsuties. but marked seborrhoeic folliculitis with pustules on face, chin, interscapular and intermammary areas. X-rays showed marked increase in density with filling in of many deposits, especially in the pelvis. In the spine further collapse of 11th dorsal vertebra present. Last seen 3.x.49 in excellent health. Therapy discontinued.

*Case* 20.

M. F—, female, aged 48, 1 child.

First seen 12.iv.48 with complaint of a mass in the left breast believed to date from a blow 2 years previously, and of decline in general health and loss of 1 stone in weight over the past 6 months.

*On examination.*—Pale, tired-looking woman with signs of recent weight loss.

*Local conditions.*—Diffuse hard mass occupying whole of left breast disc. Retracted nipple, diffuse skin attachment and deep fixation. Plaque of skin infiltration with several satellite nodules over outer half of the breast. Right breast normal.

*Regional nodes.*—Large mobile nodes both axillae, and both supraclavicular regions. In the right sternomastoid-clavicular angle was a large partially fixed hard mass 4 $\times$ 4 cm. No evidence of distant metastases. No radiological evidence of skeletal deposits.

*Treatment.*—12.iv.48. Methyltestosterone 8 $\times$ 5 mg. daily by mouth commenced.

*Progress.*—A normal menstrual period began 11 days after beginning methyltestosterone therapy. When seen again on 10.v.48 the mass in the left breast had become softer and somewhat smaller, and the right supraclavicular mass was half its former size and freely mobile. The skin deposits were paler in colour, softer and smaller. In view of this occurrence the dose of methyltestosterone was doubled. On 7.vi.48 she had gained 8 lb. in weight, her periods had not recurred, and the mass in the breast was no longer palpable. None of the nodes was more than 1 cm. across, and the skin deposits had undergone complete regression, being visible only as brownish impalpable mottlings. A section through one of these areas showed only hyaline fibrous tissue in the dermis.

By 12.vii.48 signs of masculinization had appeared, namely slight hirsuties of chin, and lips, and a deepening of the voice. She complained of slight swelling of the feet towards the end of the day. Her weight was 10 st. 12 lb., and she had gained 1 stone in weight since the commencement of treatment. At this time no vestige of primary or secondary growth remained, and the dose of methyltestosterone was reduced to 50 mg. daily, and on 23.ix.48 to 40 mg. daily. On 1.x.48 slight vaginal bleeding occurred, probably representing a menstrual period.

In January, 1949, she complained of slight backache at the end of the day. On examination small soft nodes were felt in both supraclavicular regions, but thought to be of no significance. X-rays of the spine revealed no evidence of metastases, and she was given no additional treatment. However, on 7.ii.49 she attended hospital complaining of severe backache, and on examination the nodes felt previously were found to be hard and much enlarged, numerous deposits not exceeding 3 mm. in diameter were present in the skin of the left breast, and the liver edge, which was thickened, firm and somewhat irregular was felt in the right iliac fossa. One of the skin nodules was excised, and found to contain loose columns of undifferentiated tumour cells invading dense hyaline fibrous tissue. The original primary mass in the breast was not felt. The dose of methyltestosterone was increased to 100 mg. daily, but deterioration continued.

*Case* 28.

E. H—, aged 71, nullipara.

Mass present in left breast since 1943 and probably earlier. " Burst " towards end of 1946, and continued to spread and discharge until January, 1948, when patient noticed a small lump on opposite breast, and sought medical advice for the first time.

*Condition on* 9.ii.48.—Very thin, frail old woman of low intelligence.

*Local condition.*—*Left breast* replaced by a hard nodular tumour deeply attached to muscle, and infiltrating skin over it. The upper outer quadrant deeply ulcerated, and the excavated floor heavily infected, the edges red, hard, raised and nodular. The upper edge of the ulcer involved the contiguous axillary mass of nodes which was fixed to the surrounding structures. Nipple retracted and mainly embedded in and replaced by growth. *Right breast* : Atrophic. Hard mass in axillary tail 2 × 2 cm., mobile, lightly attached to skin.

*Regional nodes.*—Large mass of hard fused fixed nodes left supraclavicular region. Several hard mobile nodes right side and right axilla.

*Skin.*—Two small nodules 0·5 cm. in diameter in skin of right anterior chest wall.

*Treatment.*—10.ii.48 : Implantation of 5 × 100 mg. pellets fused testosterone.

*Progress.*—One month later the mass in the right axillary tail had increased slightly in size, as had the mass of left supraclavicular nodes. A hard subcutaneous nodule 1 cm. across, not attached to the skin, was felt over the left scapular region. Five months later it was clear that considerable extension of the primary mass had occurred, and the ulcerated part had encroached further into the axilla.

Treatment with 15 mg. stilboestrol daily by mouth was commenced on 12.vii.48, and by 30.viii.48 marked regression of the primary mass, of the mass in the right axillary tail and of the 3 cutaneous deposits had taken place, though little change in the nodes was noted.

By 26.ix.48 the ulcerated part of the primary growth had completely healed ; the mass in the right axillary tail, and the skin deposits, could not be found. She took stilboestrol (15 mg. daily) until 20.vi.49, when an irregular contracted tethered pale scar was all that remained of the left breast. The very hard masses of nodes remained unchanged throughout the observation period of 19 months. By 5.ix.49 several small nodules which had appeared at the periphery of the scar during the previous 3 months were shown to contain active malignant cells.

*Case* 37.

M. K—, female, aged 37. Nullipara.

1943.—First noticed lump in left breast.

1944.—Left radical mastectomy.

1947.—Onset of pain in right shoulder and neck in January, increasing in severity during the year, and appearing in back, left shoulder and hips towards the end of the year.

*Nov.*, 1947.—Confined to bed owing to severity of pain, and its exacerbation by slight movements of head, trunk or limbs.

16.xii.47.—Admitted to Royal Cancer Hospital.

On examination she was in good general condition, but bedridden with crippling pain. There were 3 ulcerated recurrences along the left mastectomy scar, the two larger measuring 2 × 4 and 5 × 3 cm. respectively, with irregular thickened raised edges, fixed bases and coarsely nodular floors. The opposite breast was uniformly hard, fixed to pectoral fascia, and to the skin by multiple points of attachment. There was extensive involvement of right axillary and both supraclavicular groups of nodes, and a tender diffuse bony swelling of the medial end of the right clavicle. There were 3 skin metastases, one 3 cm. in diameter in the scalp over the left parietal region, two smaller deposits, one in the left lumbar region, one in the left groin. Radiologically there was extensive infiltration of the entire vertebral column, and there were multiple deposits in the ribs, clavicles and pelvis. Mobility of the head and neck and of the right shoulder-joint was limited to a few degrees in all directions by pain, and the slightest active movement of the legs aggravated the neck pain. The left shoulder was affected to a lesser extent.

*Other investigations.*—Hb 86 per cent, W.B.C. 12,800, serum calcium 12·0 mg./100 c.c. Serum alkaline phosphatase 49·2 units, serum phosphate 3·3 mg./100 c.c.

*Treatment.*—For the first few days in hospital bed rest, analgesics and sedatives alone were given. The pains were usually relieved by Mist. A.P.C. and nepenthe (g. 30).

31.xii.48.—500 mg. testosterone was implanted in the subcutaneous abdominal fat in 10 pellets of 50 mg. fused testosterone.

*Progress.*—Two days after the testosterone implantation the pains were distinctly better, the nepenthe mixture was no longer necessary, and during the subsequent fortnight she became well enough to get up and walk. From then on she continued to improve, and at the end of 3 months had regained full movements of head and neck, spine and shoulders. By the middle of April she had gained 9 lb. in weight and was leading a normal life. Menstruation, which had ceased after the implantation, recurred on 19.iv.48, and on 29.iv.48 a further 1000 mg. testosterone in 10 pellets of 100 mg. was implanted.

During this period the ulcerated chest wall recurrences were undergoing slow regression; the skin deposits on trunk and scalp regressed completely, the latter leaving only a flat hairless plaque of skin. The right breast had become normal in consistence and mobility, and the attachments to the skin over it could no longer be demonstrated. The clavicles and vertebral spines were no longer tender to moderate percussion; the supraclavicular nodes on both sides diminished greatly in size, but the right axillary nodes remained unchanged, though no longer tender. X-rays showed increased calcification round many deposits.

By August, 1948, two of the ulcers on the chest wall had healed, but the third, although flatter, had increased slightly in diameter, and shortly after the patient complained of pains on the left side of the neck, shoulders, back and hips. These pains were found to be relieved by 50 mg. of oral methyltestosterone daily, but again recurred when the dose was reduced to 30 mg. daily.

However, by 5.iv.49 there was marked deterioration in general and local condition. She had lost 12 lb. in 9 months, had recurrence of low back pain; the mass in the upper inner quadrant of the right breast was larger, as were the axillary nodes; the bony parasternal swelling in the left 4th interspace had ulcerated through the skin. There were numerous hemispherical bony projections on the vault of the skull, on the frontal bone, on the right clavicle. X-rays showed many fresh osteolytic deposits in spine, ribs and pelvis, side by side with old sclerosed deposits, which were less dense than before.

Injections of testosterone propionate 100 mg. 3 times weekly were begun, but one month later there was no improvement and hormone therapy was discontinued. Her condition rapidly improved thereafter; she was given a short course of palliative X-rays to the lumbar spine and to the fungating chest wall deposit. When last seen on 6.ix.49 she was pain-free, well and active, and the right breast mass and axillary nodes were much smaller, and the bony projections on skull and clavicle were no longer felt.

*Side effects of testosterone.*—In this patient the side effects of the testosterone therapy were less marked than in many others, although she was under the influence of androgens for 16 months. Facial hirsuties was minimal, and there was no voice change, and no oedema of the ankles. However, general increase in skin thickness occurred, especially over the face, neck and back, its texture became more coarse, and it was more greasy than before. In the fortnight following the second implantation she complained of pruritus vulvae, and pustular acne appeared on the face, though the more common diffuse inflammation of the sebaceous duct orifices was not marked. There was marked hypertrophy of the clitoris, and an increase in the amount of perineal hair. The redistribution of subcutaneous fat reduced the feminine contours of the body to some extent, and this, together with the change in skin texture, probably accounted for the changed facial appearance noticed by her friends.

*Case* 12.

V. C—, nullipara, aged 52. Menopause at 45.

1945.—Radical mastectomy for Paget's disease of nipple.

*June*, 1948.—Onset of backache. Metastases in dorsal spine. Pain temporarily relieved by radiotherapy.

*July*, 1948.—Recurrence of pain, now involving neck, hips and both thighs.

*X-rays*.—Extensive destruction of pelvic bones and femora by metastases.

23.ix.48.—Implantation 1400 mg. (14 × 100 mg.) testosterone.

*Progress*.—No relief of pain.

*Case* 25 (Fig. 1 and 2).

M. G—, aged 38, nullipara.

11.viii.44.—Left radical mastectomy for Stage I carcinoma of breast. Post-operative radiotherapy.

Remained well until April, 1948, when first noticed breathlessness on effort, which subsequently became worse.

*June*, 1948.—Signs of large left pleural effusion. No other clinical evidence of recurrence. X-ray chest : Large left-sided effusion. Multiple small opacities both lung fields. Pleural fluid : opalescent straw-coloured fluid. Total protein content 4·45 mg./100 ml. Histology of deposit : Many malignant cells, some of them grouped to form tubules ; secondary adenocarcinoma.

*Treatment*.—29.vi.48 : Implantation of 10 pellets of 100 mg. testosterone.

*Progress*.—For the first month after implantation dyspnoea was still increasing, and the patient was unable to continue with her work as telephone exchange supervisor, but during the second month rapid improvement ensued, and she was back at full work 6 weeks after implantation. She has remained symptom-free since, the signs of effusion became gradually less, and on 3.ii.49 the only abnormality found was slightly diminished expansion at the left base. Radiologically there is now only obliteration of the left costophrenic sulcus, and the parenchymal opacities are no longer visible. She was last seen on 25.viii.49.

*Side effects*.—Cessation of menstruation from 29.vi.48 to 17.i.49. Periods since regular. Seborrhoeic folliculitis noted 26.viii.48, huskiness of voice 23.ix.48, loss of hair from both temples 18.xi.48.

*Case* 13.

L. C—, nullipara, aged 39. Menstruation regular 5/21, onset 15 years.

27.ix.48.—Left radical mastectomy for painless lump present 1 month. Single hard axillary node.

10.iii.49.—Several small subcutaneous nodules in mesial skin flap of scar found.

21.iii.49.—Rapidly increasing dyspnoea during past fortnight. Loss of appetite. Large left pleural effusion. Fluid contained malignant cells.

*Treatment*.—21.iii.49. Radiotherapy to skin recurrences commenced.

30.iii.49.—Methyltestosterone 50 mg. daily commenced.

28.iv.49.—Dyspnoea not relieved. Aspirated from left pleural cavity 32 oz. fluid. Dose of methyltestosterone increased to 100 mg. daily.

5.v.49.—Further 3 pints fluid aspirated from pleural cavity.

*Progress.*—Steady deterioration. Methyltestosterone was discontinued on 21.vi.49. By 12.ix.49 widespread deposits in skin, lungs, skeleton and liver.

*Case* 44 (Fig. 3 and 4).

E. M—, aged 48.

*June*, 1943.—Subradical mastectomy at 12th week of pregnancy for lump in left breast first noticed several months earlier.

1946.—Noticed several small nodules in neighbourhood of scar, soon followed by others, gradually increasing in size with confluence and spreading ulceration.

10.vii.48.—Admitted to Royal Cancer Hospital complaining of recent loss of weight, and swelling of left arm.

*On examination.*—Good general condition. Local condition: Multiple areas of ulceration with confluence over a wide area on either side of the mastectomy scar extending to the apex of the axilla above and to the level of the 8th rib in the mid-clavicular line below. Most of the raw surface, which was finely granular, was covered by thick crusts of solid exudate, and the edges of the ulcer were only slightly raised above the level of the surrounding skin, and had not the appearance of a rapidly growing neoplasm. Several shotty cervical nodes on both sides. Enlarged right axillary nodes.

*Treatment.*—The crusts were soaked off with warm saline, exposing the entire ulcerated surface. Soft paraffin gauze dressing.

16.vii.48.—Tablets methyltestosterone 50 mg. daily commenced.

*Progress.*—Menstrual periods ceased after commencement of treatment. Within 5 weeks the surface of the edge of the ulcerated areas had merged with the adjacent skin surface, and in places opalescent epithelium could be seen growing over the surface of the ulcer, now uniformly smooth. By 23.ix.48 large areas had become epithelialized from the periphery, and by 28.x.48 only one small area about 1 cm. square in the upper part of the ulcer was unhealed. The left arm was less swollen, and the patient had gained 21 lb. in weight. There was marked seborrhoeic folliculitis on the skin of the back, and some larger pustules on the chin. There was coarsening of the facial skin, but no marked hirsuties and no voice change. The dose was reduced to 35 mg. daily. By 2.xii.48 the entire area was paler in colour, the young skin over it thicker, but there was still one small discharging area. This dried up during the next two months, dressings were discarded and therapy discontinued, but on 1.ix.49 two hard pinkish nodules a few millimetres in diameter appeared at its margins, sections of one of which showed active scattered carcinoma cells in fibrous tissue.

### Addendum.

Since the manuscript was completed M.K. (Case 37) has attended (28.2.50) with recurrence of pain, M.G. (Case 25) is still in remission (16.2.50). The lesions of E.M. (Case 44) was unchanged on 8.12.49, and K.B. (Case 3) was in remission on 13.2.50, having had a second course of methyltestosterone 50 mg. daily since 12.12.49 for recurrence of right shoulder pain and stiffness. V.C. (Case 12), L.C. (Case 13) and M.F. (Case 20) have died.


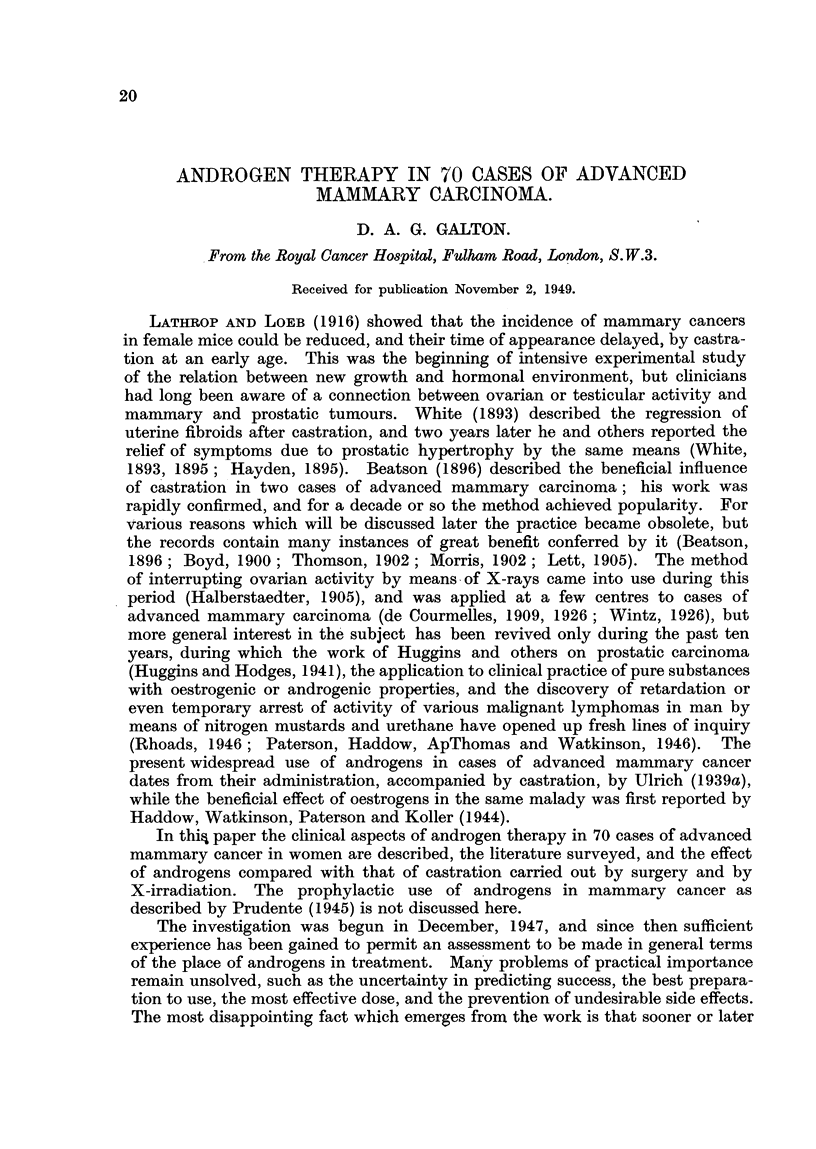

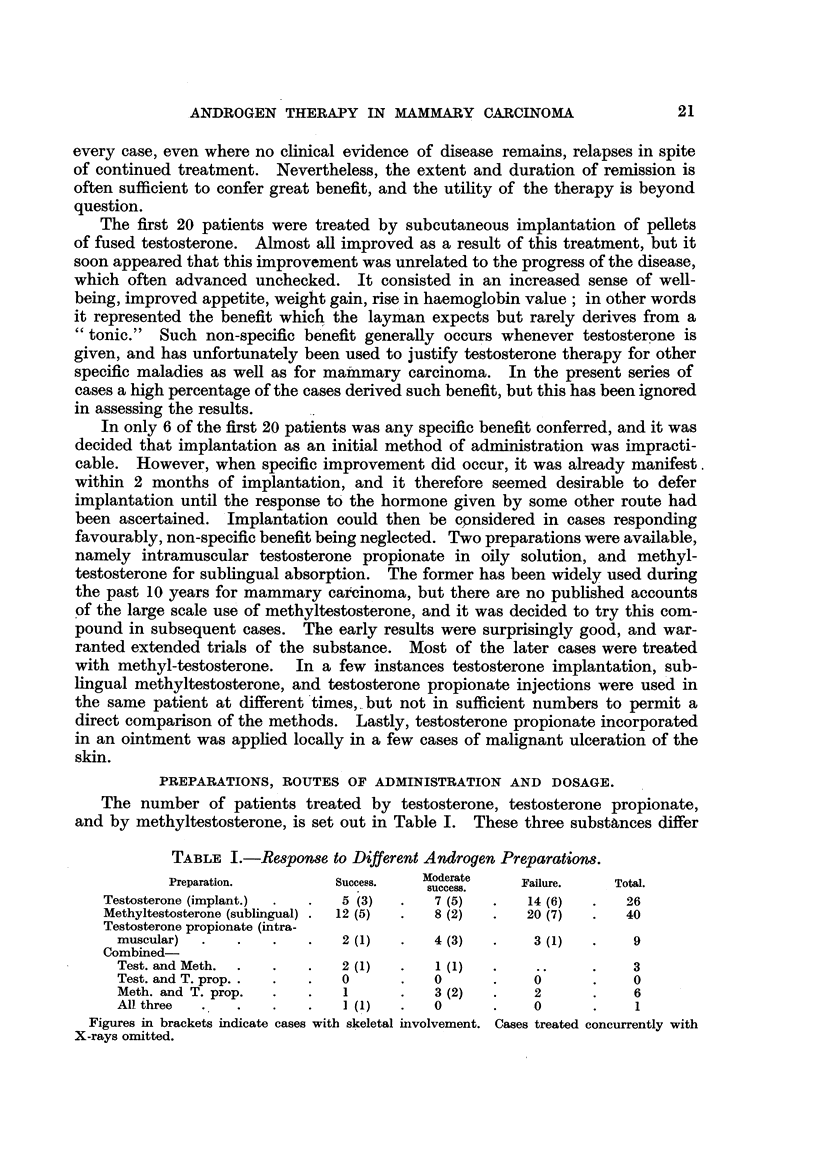

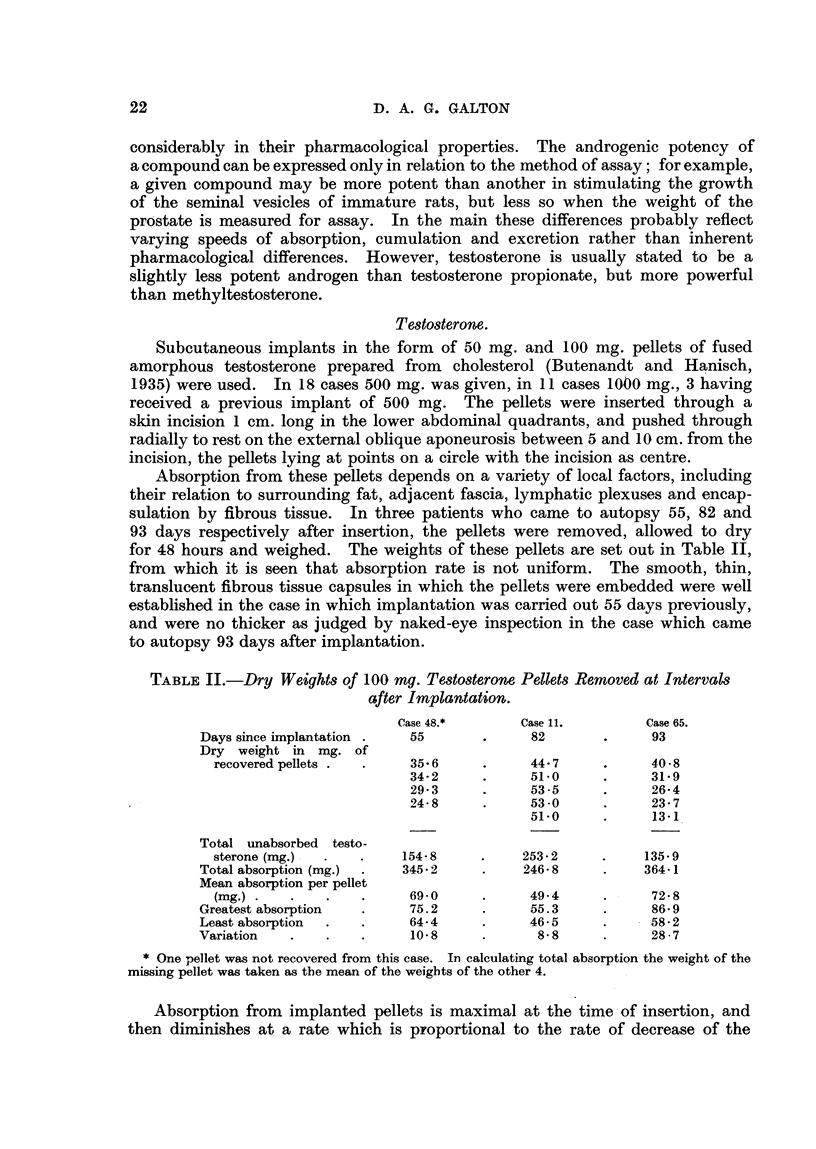

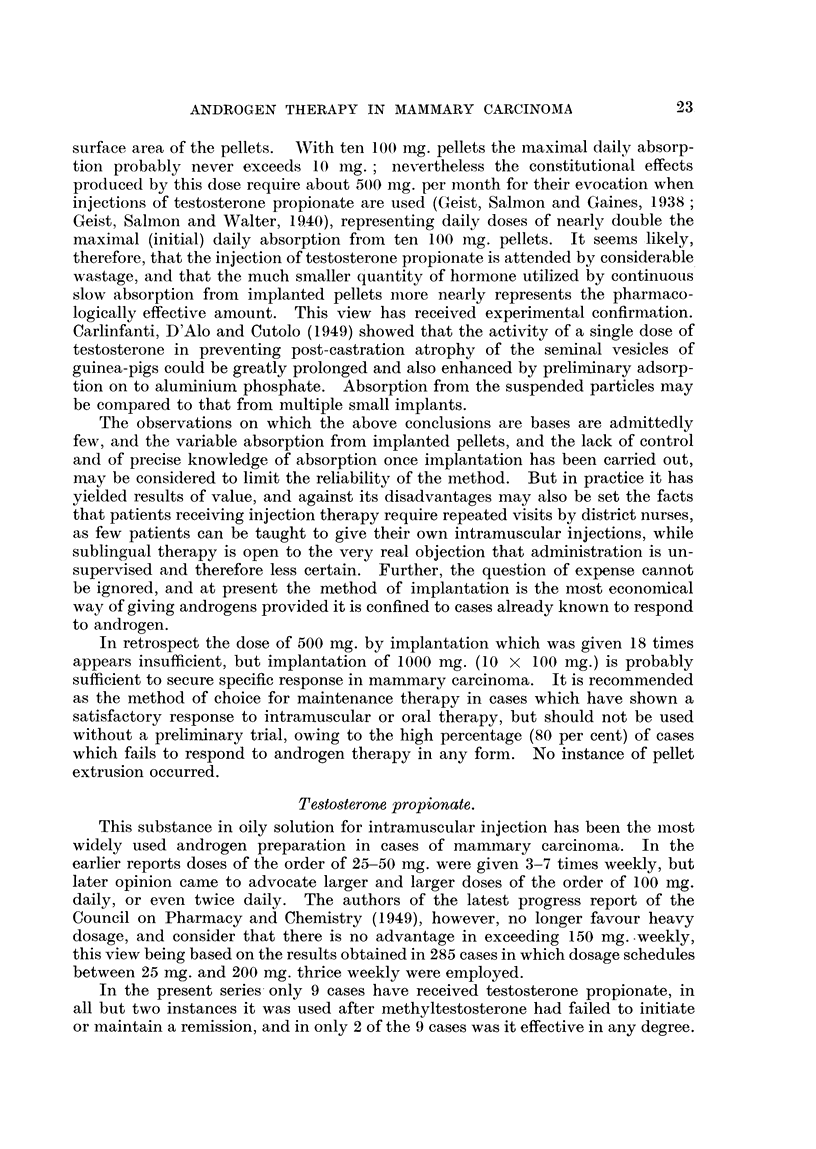

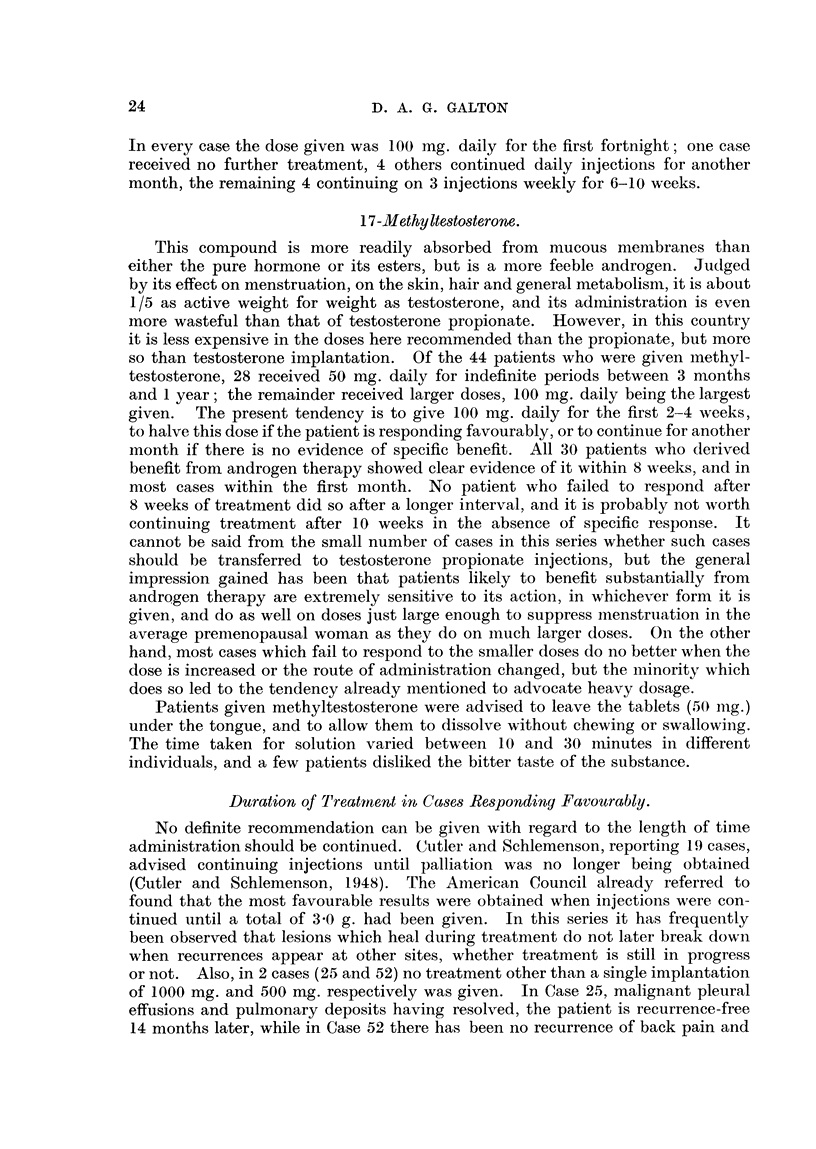

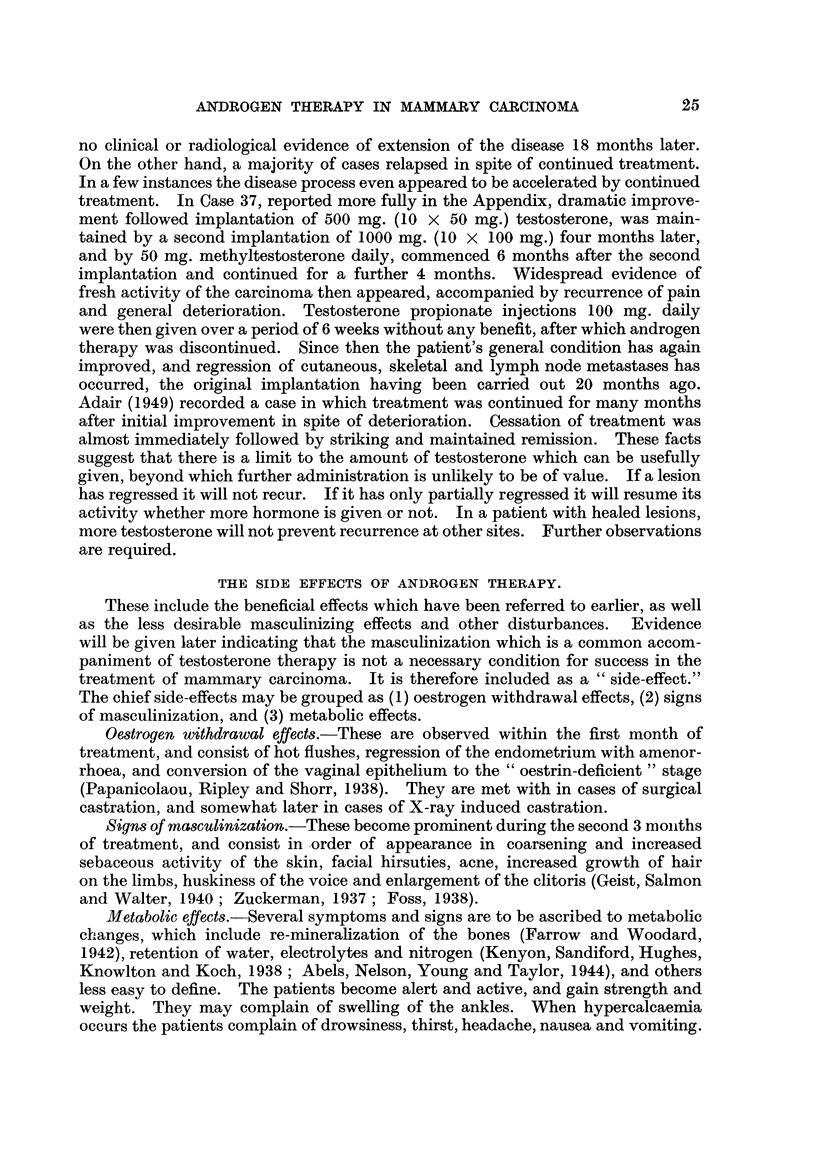

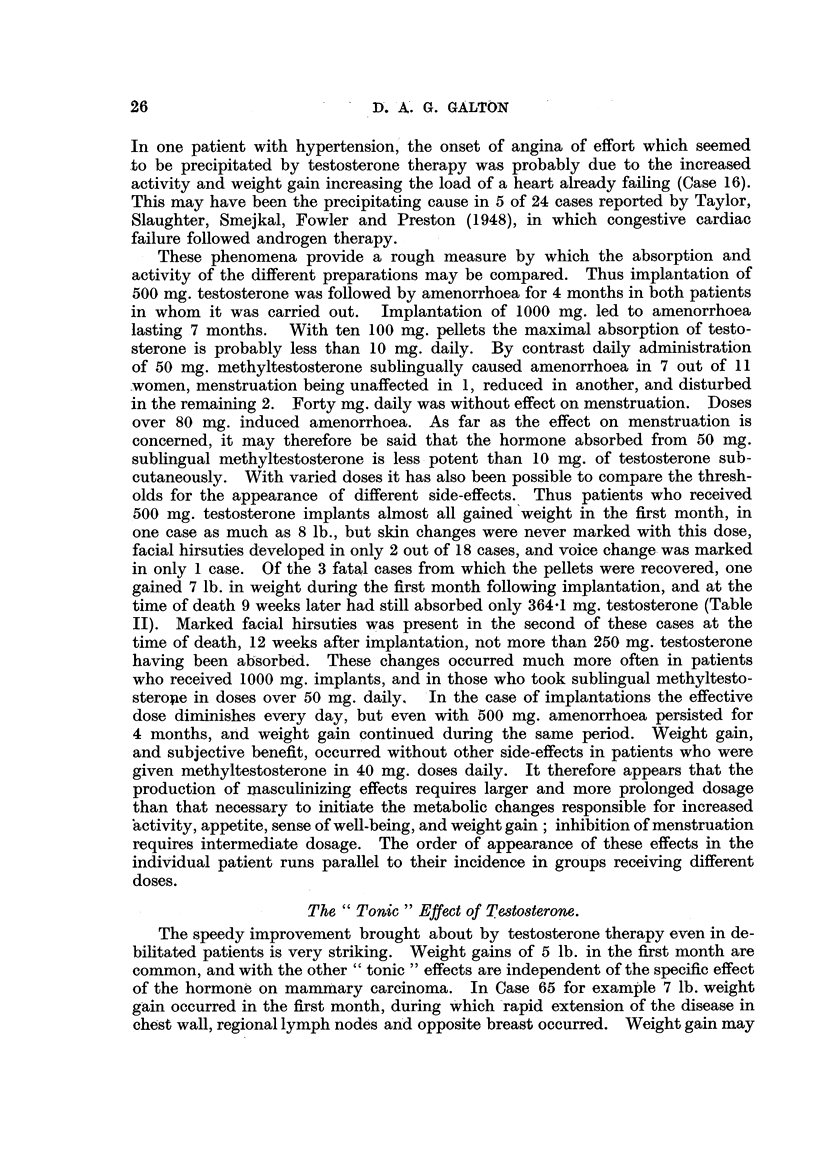

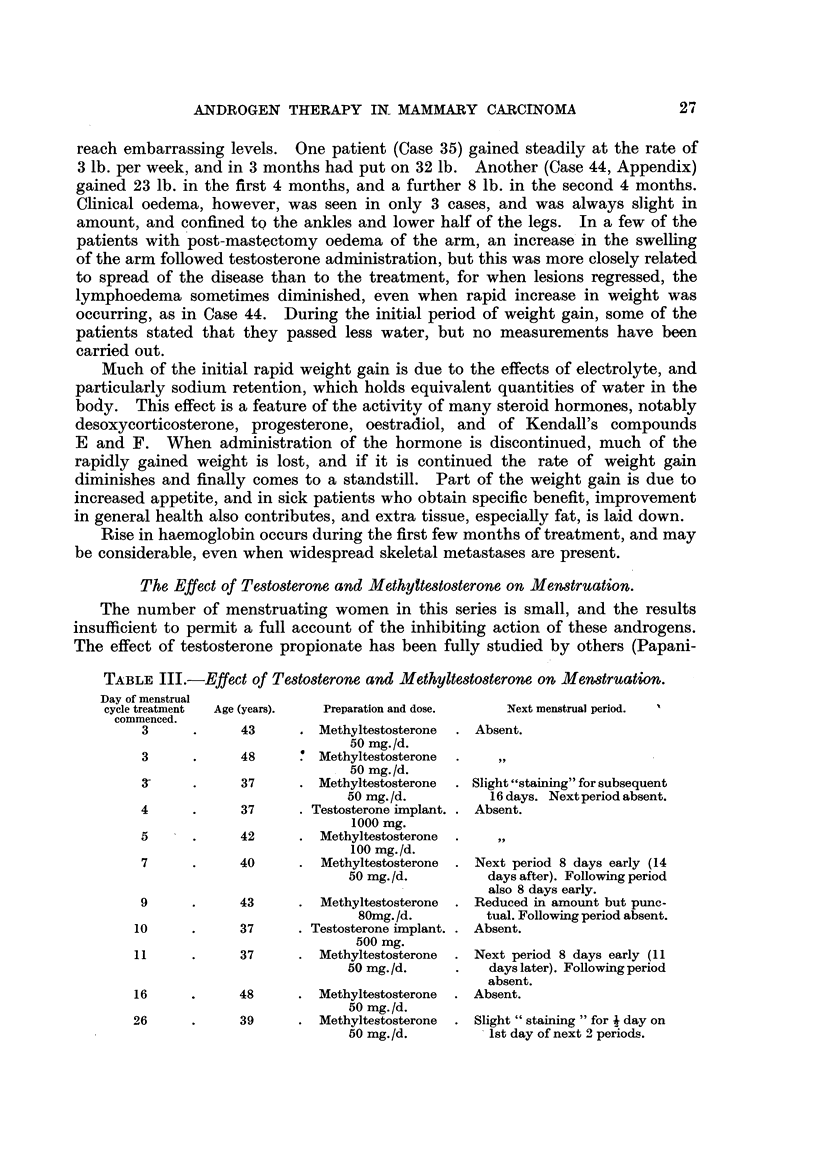

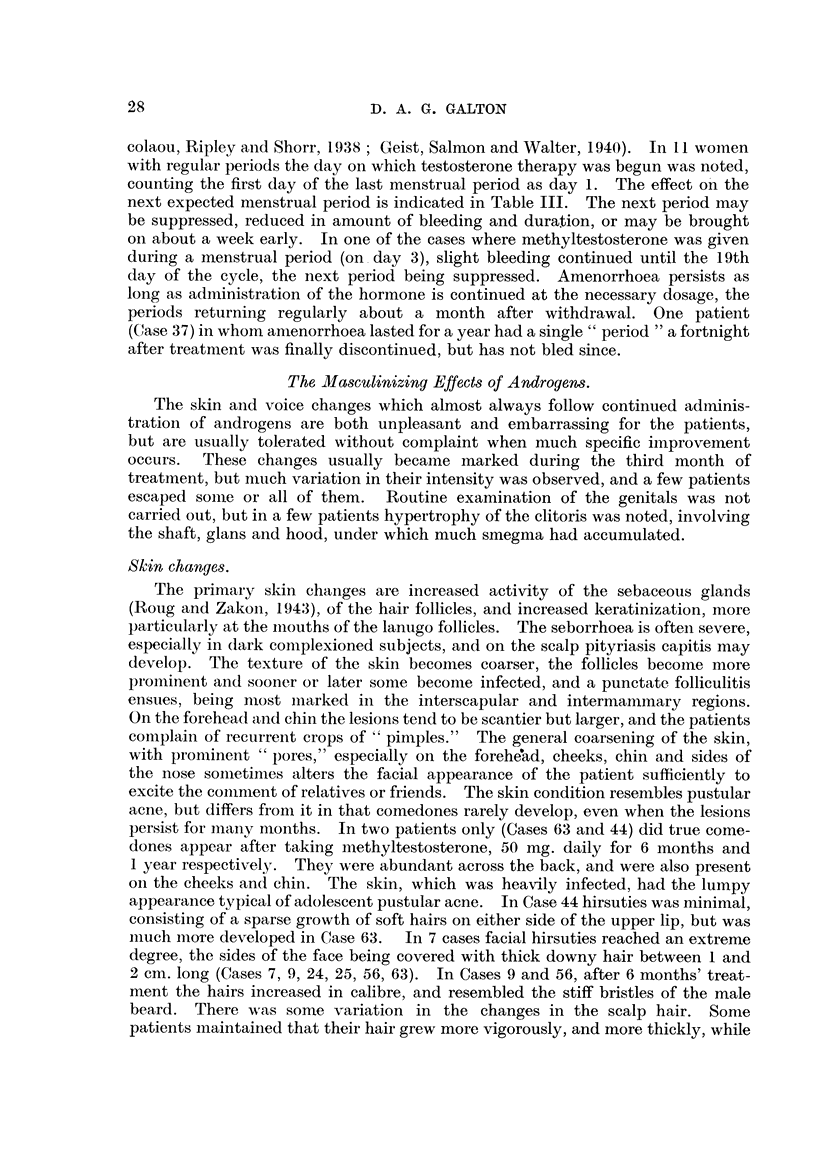

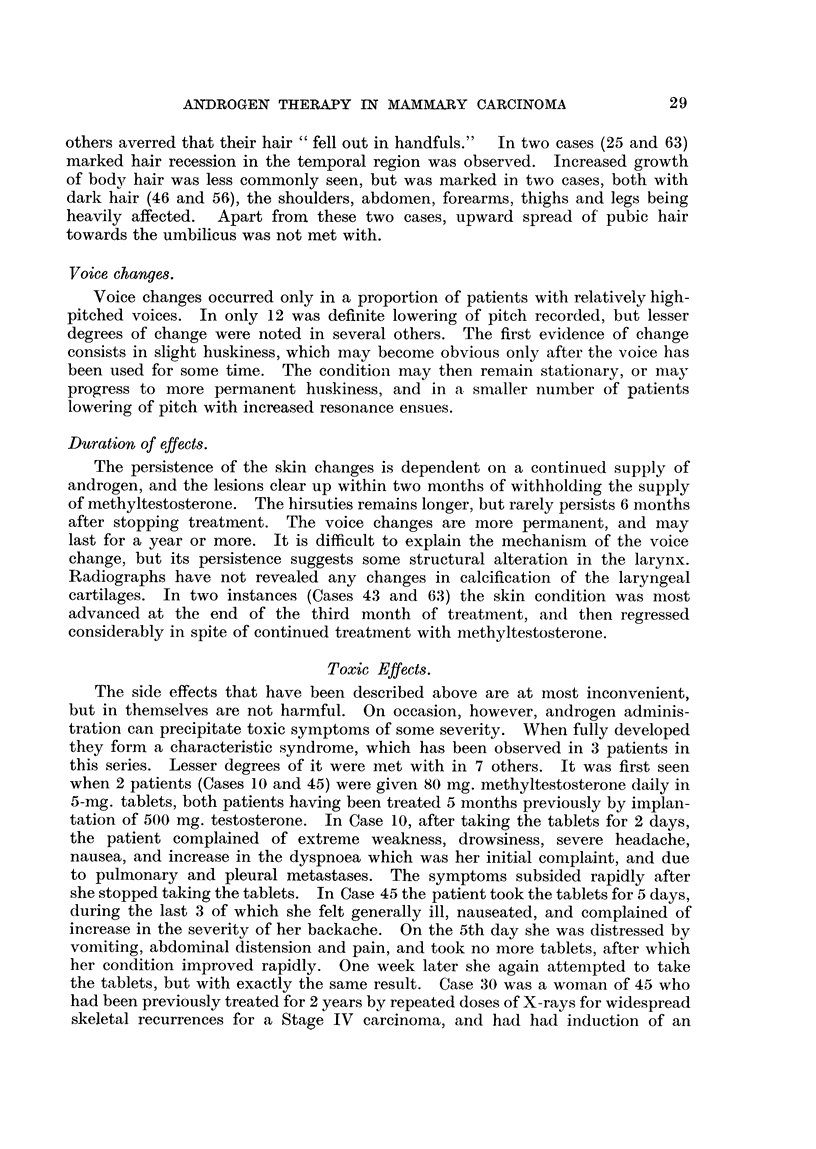

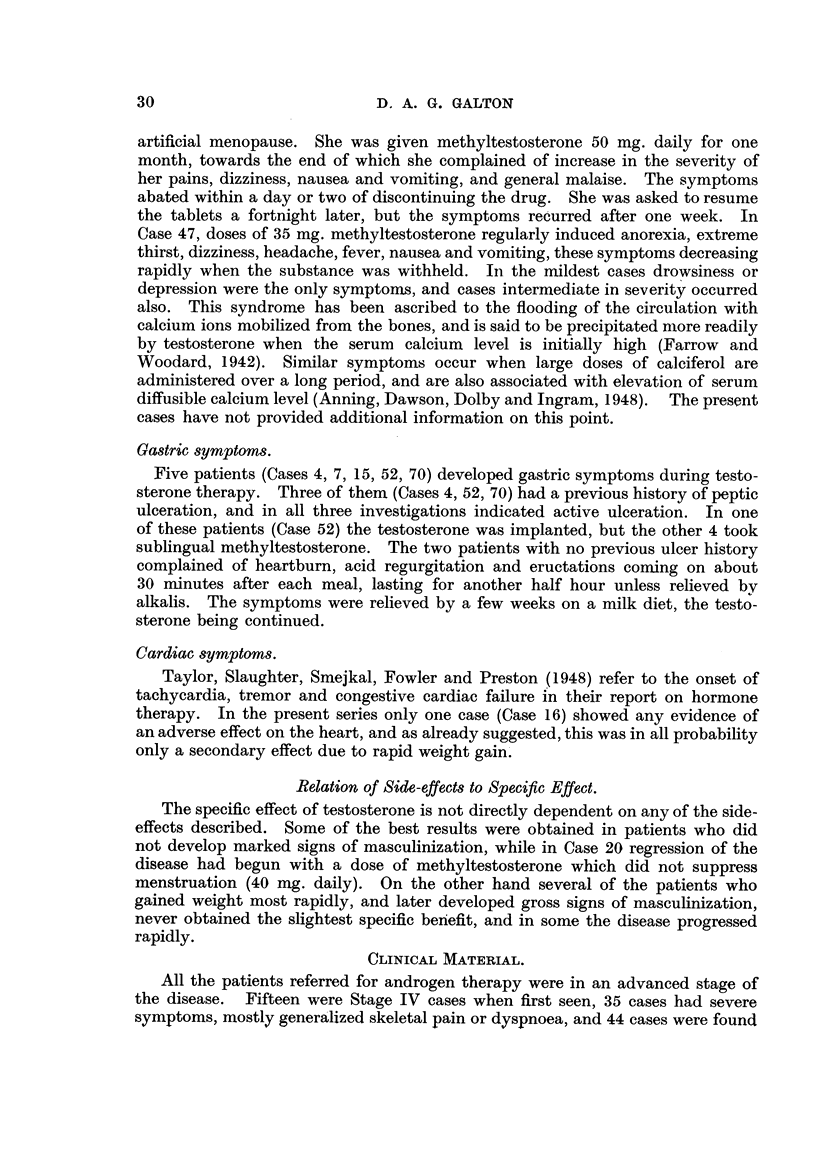

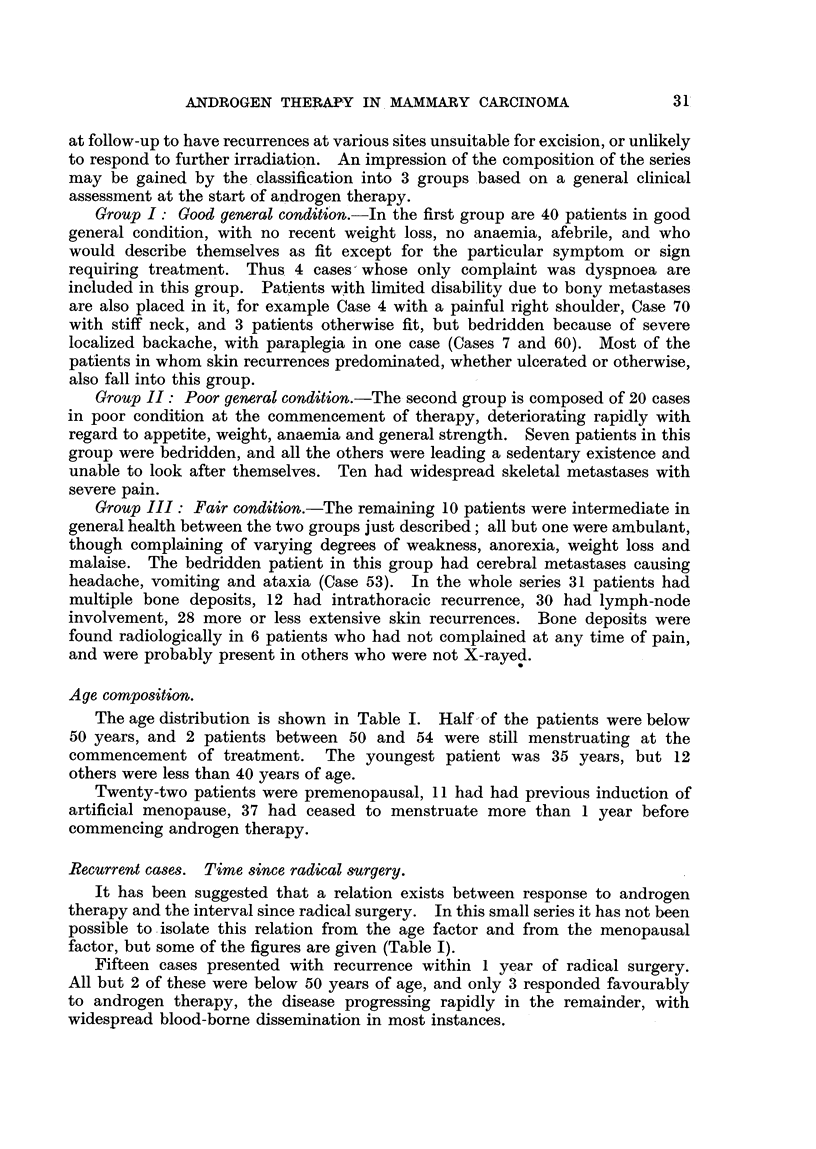

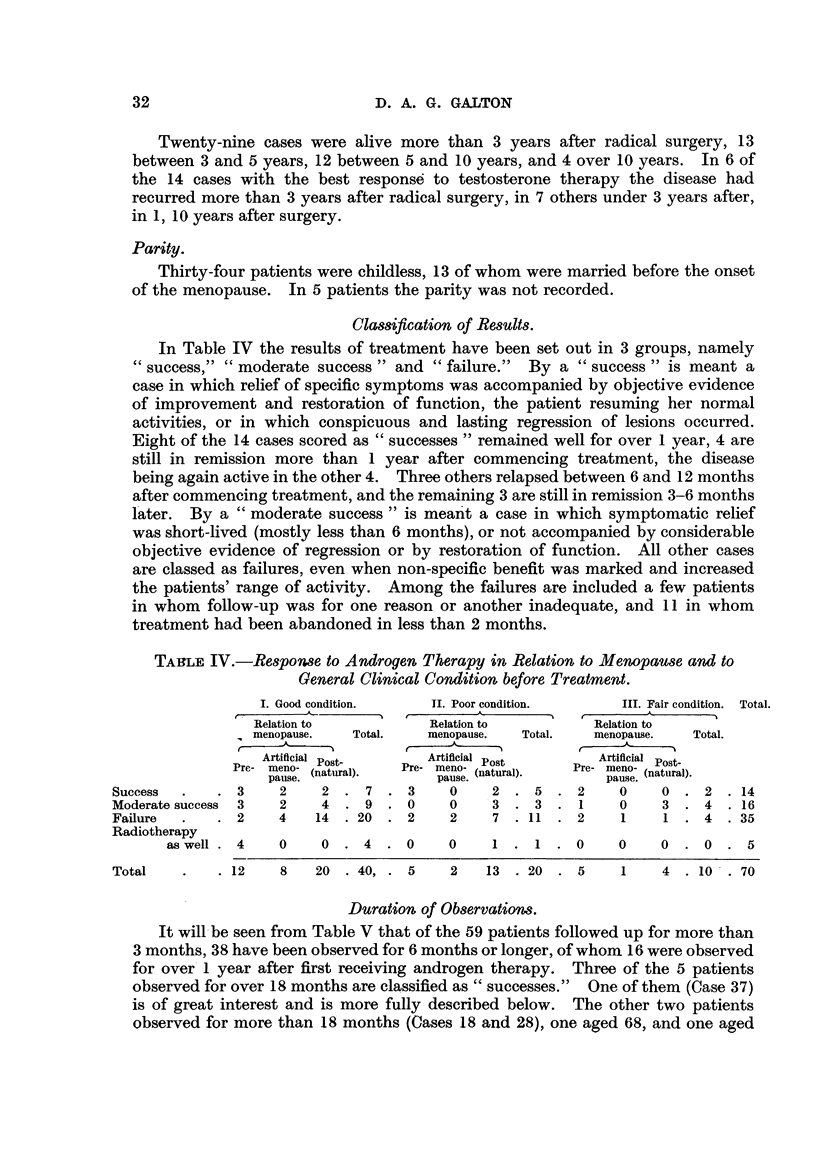

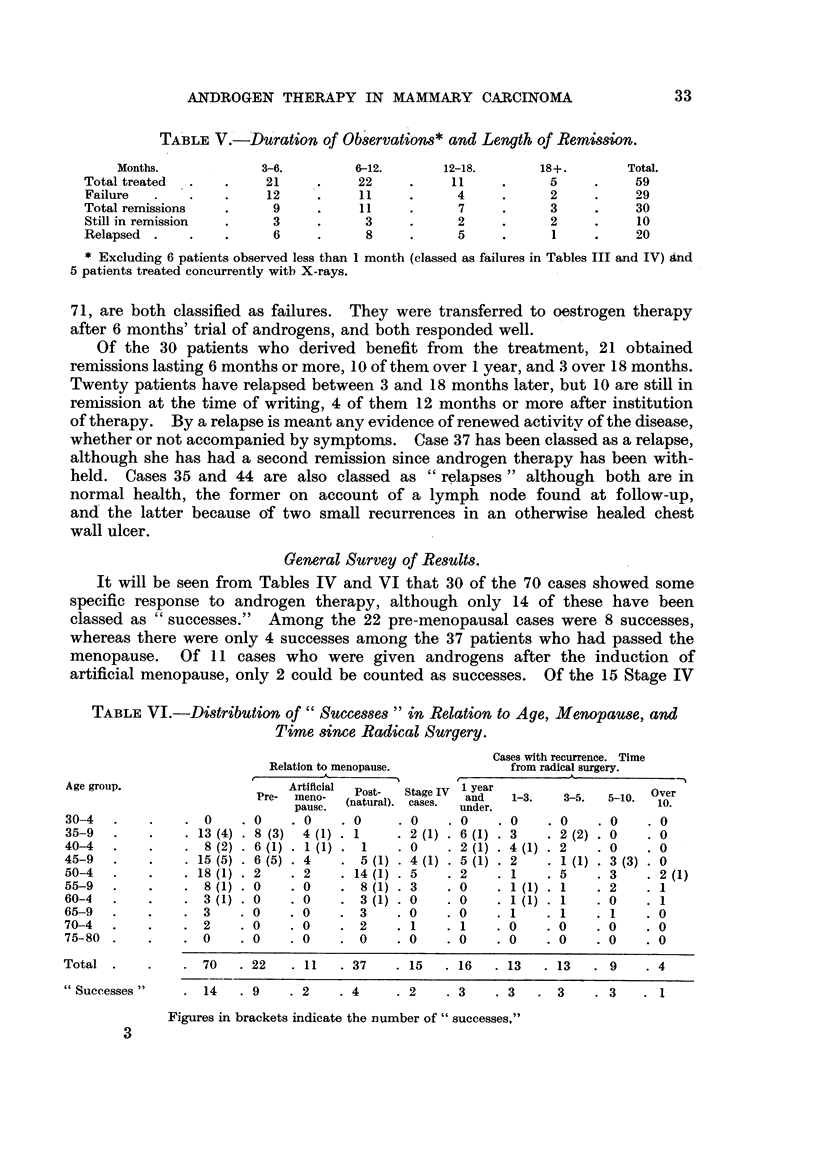

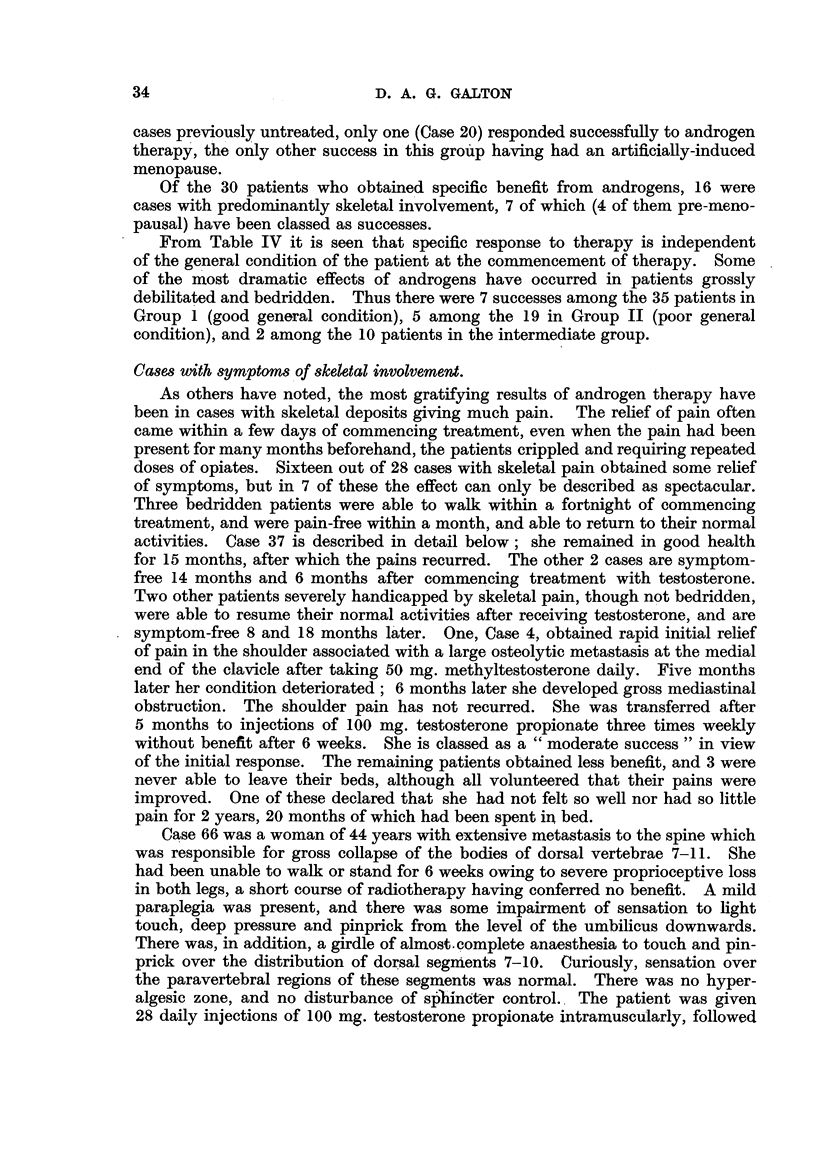

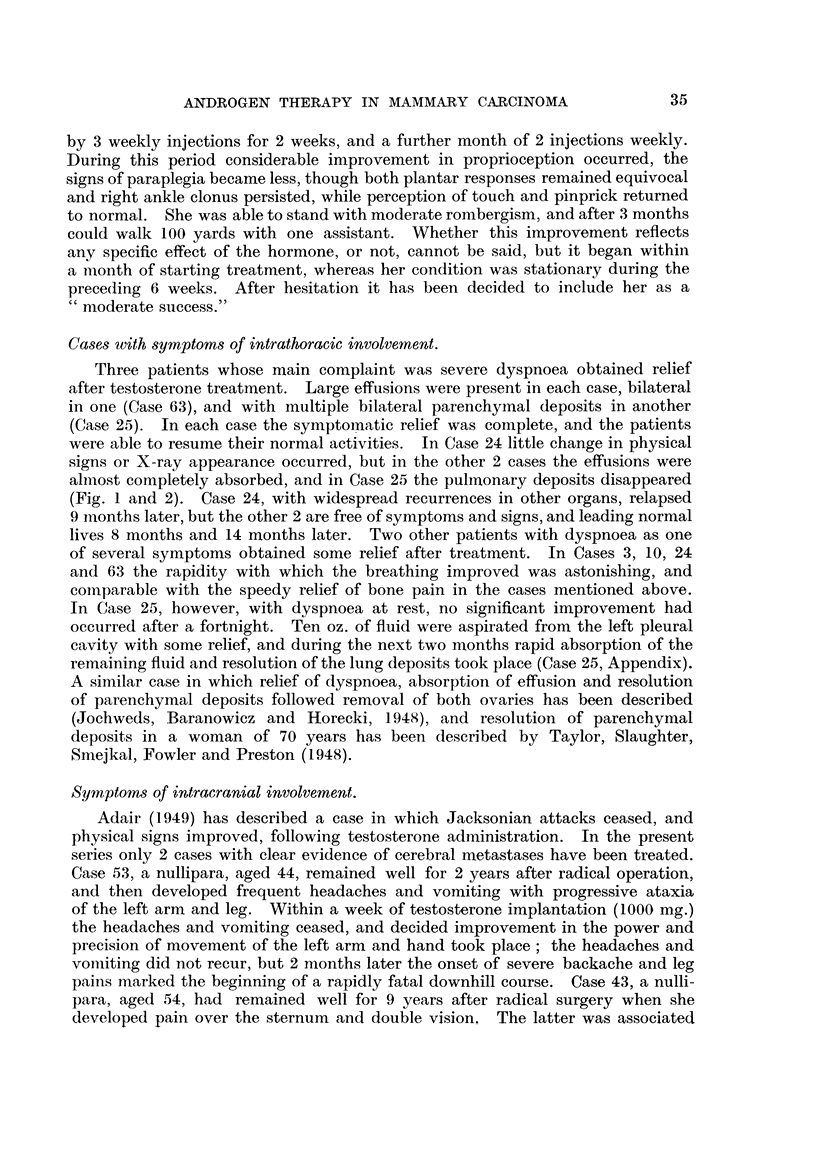

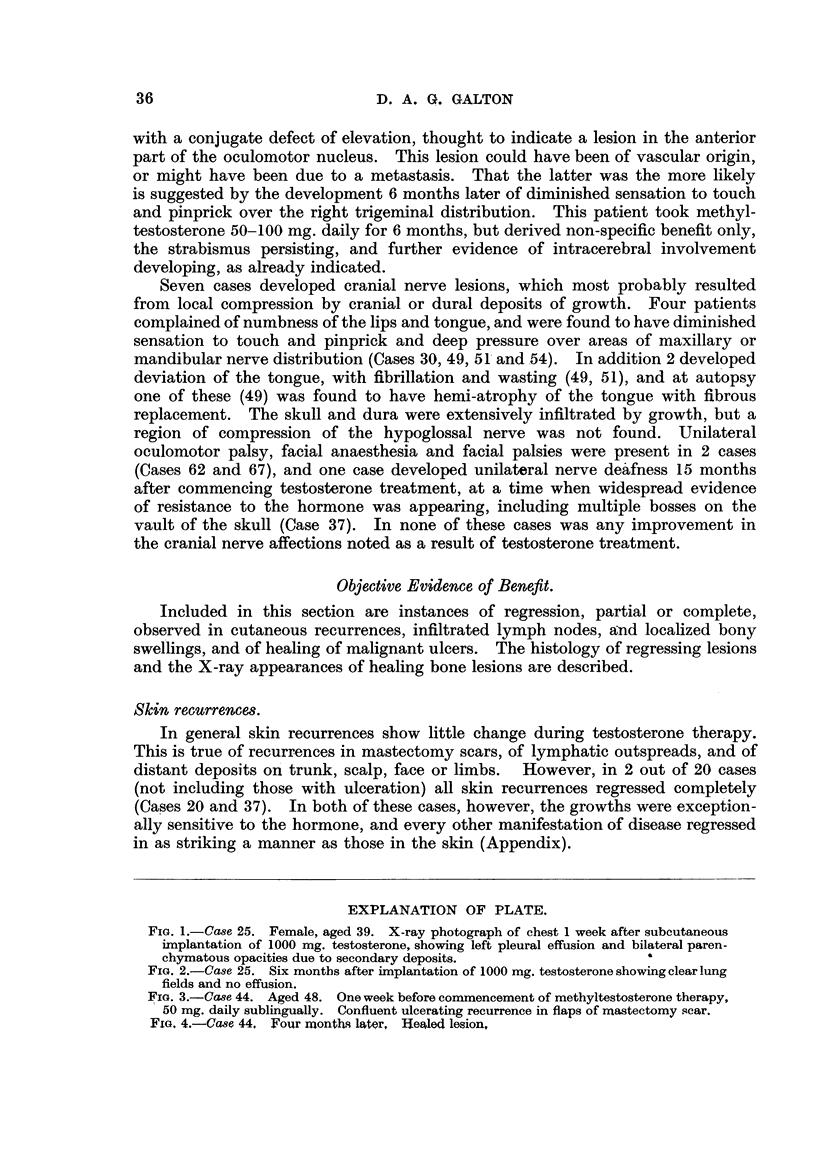

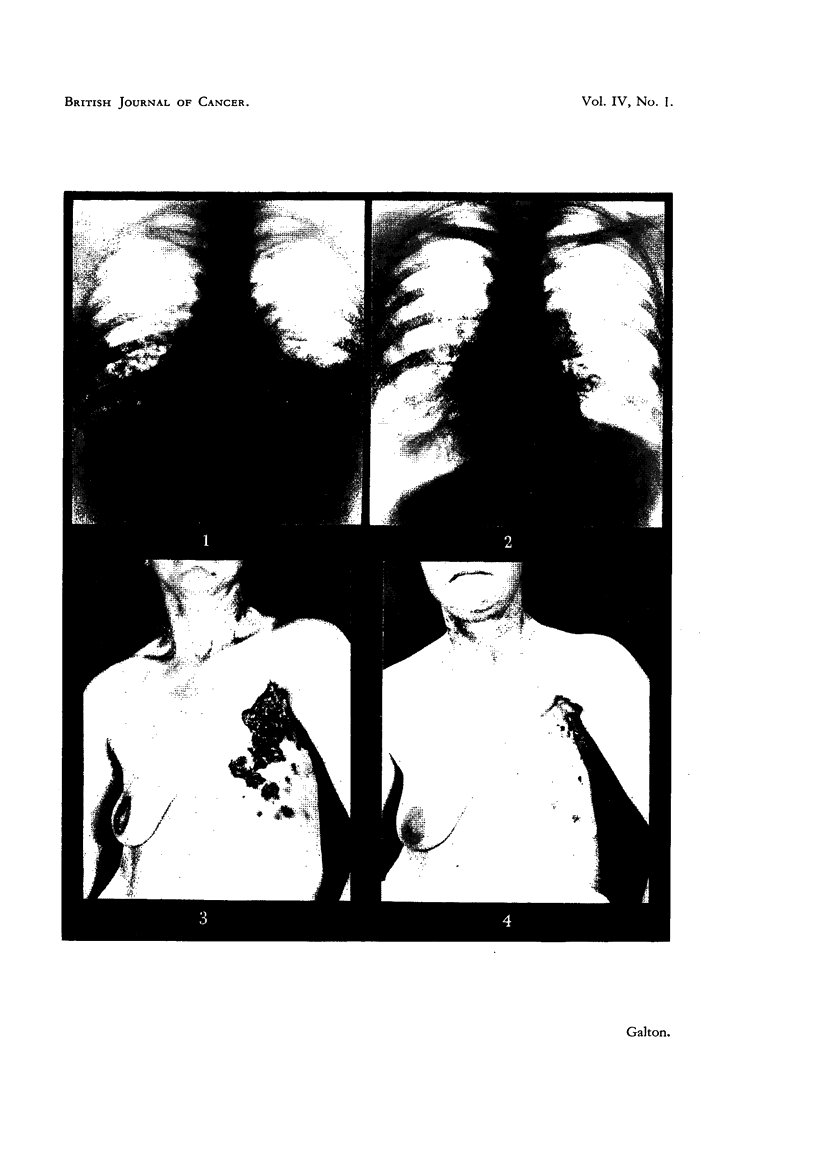

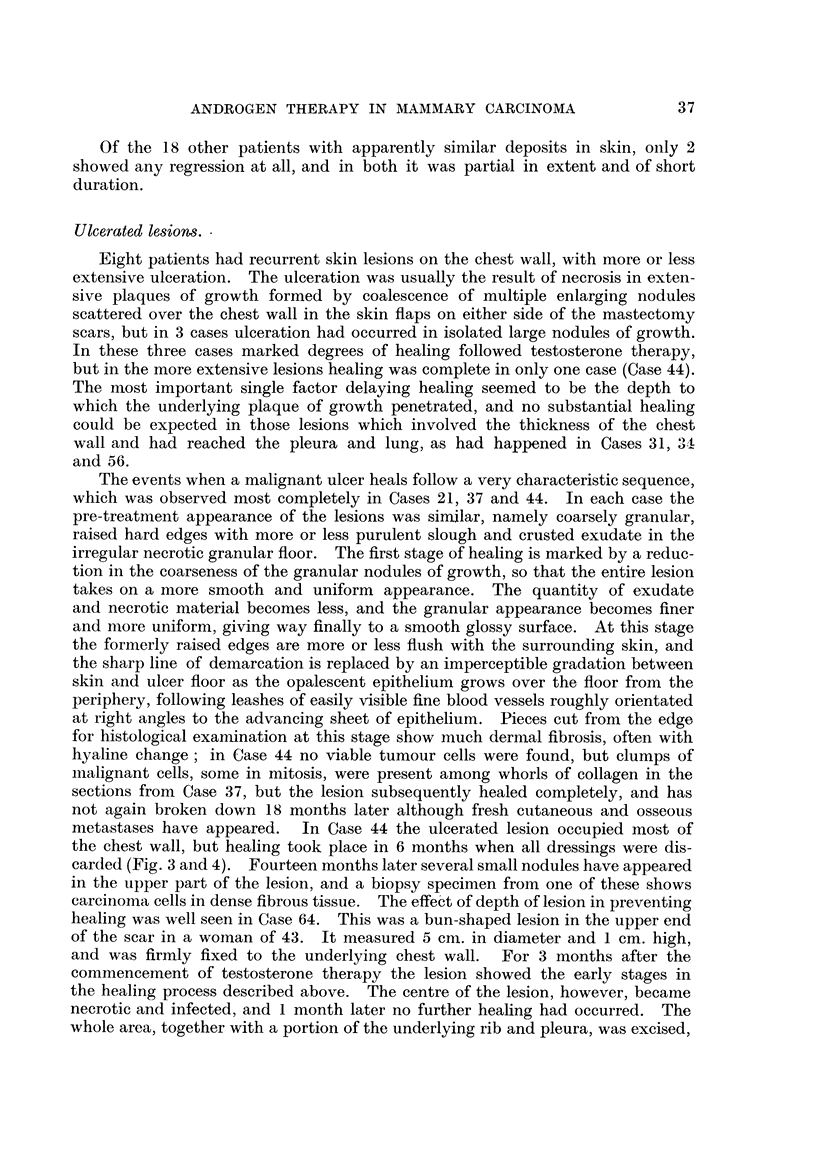

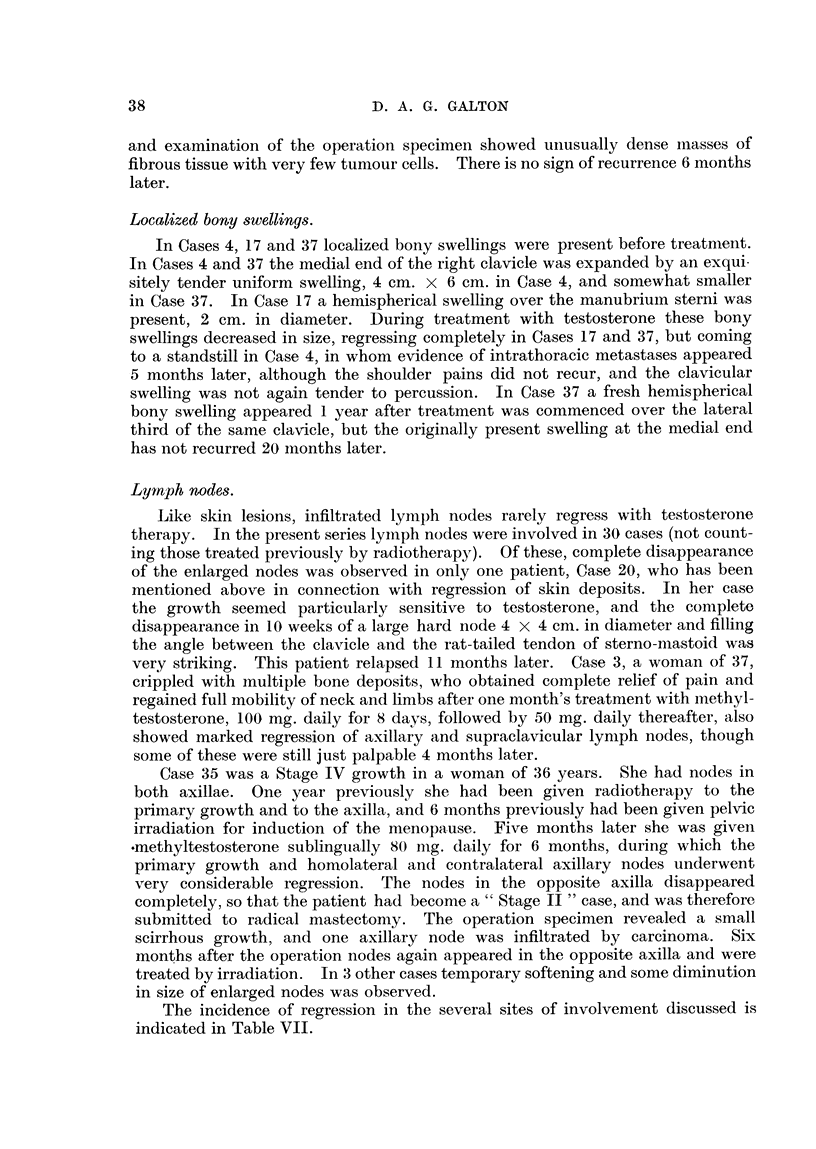

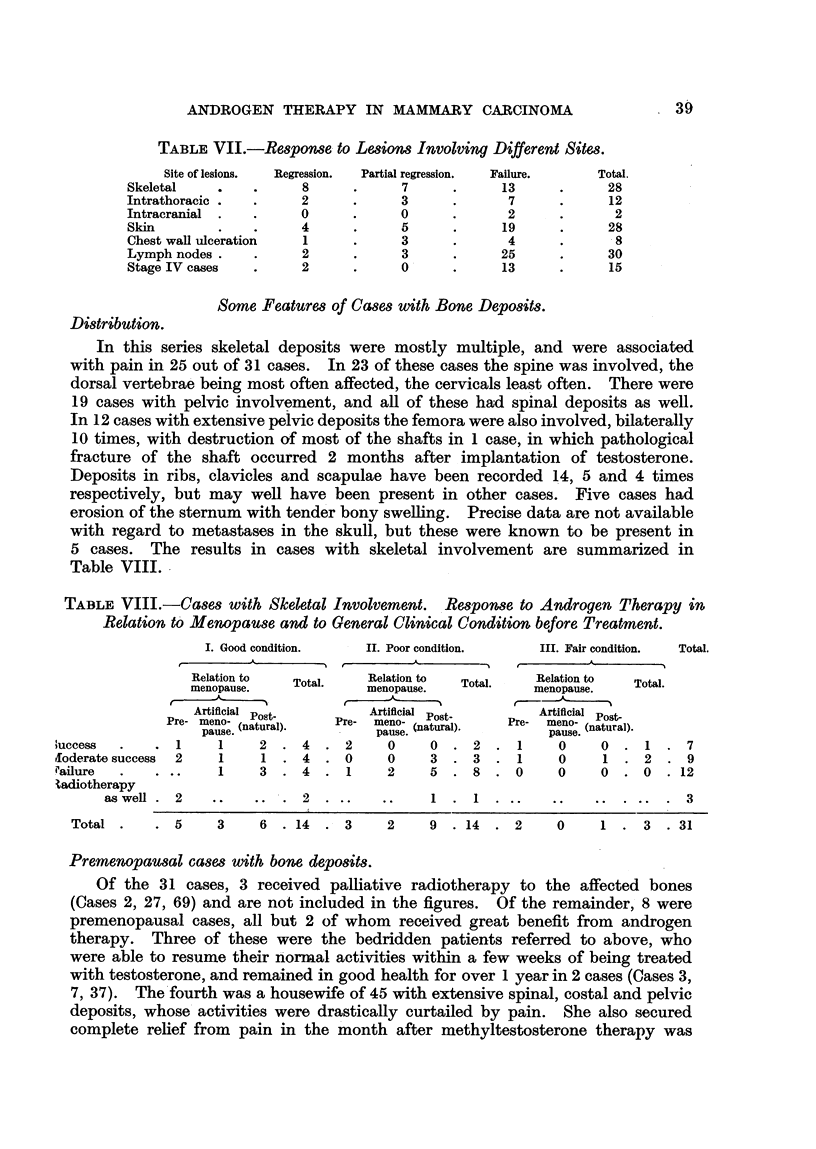

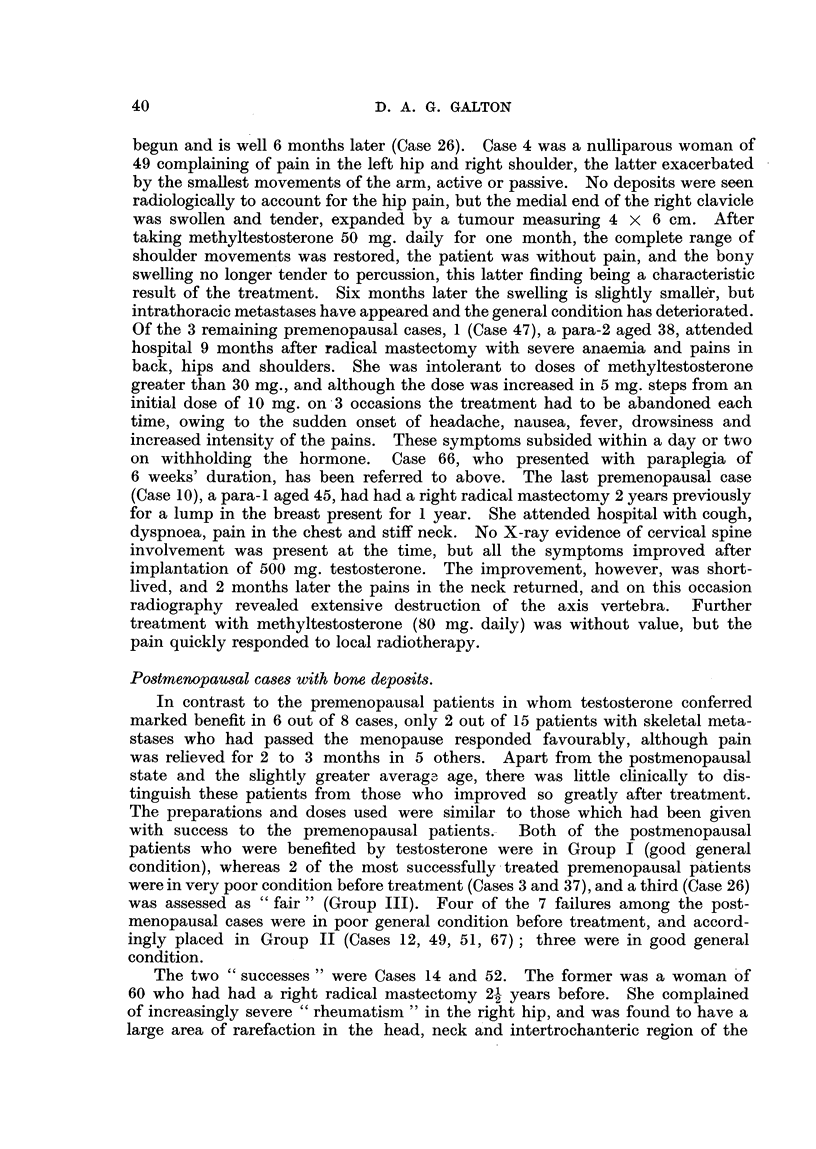

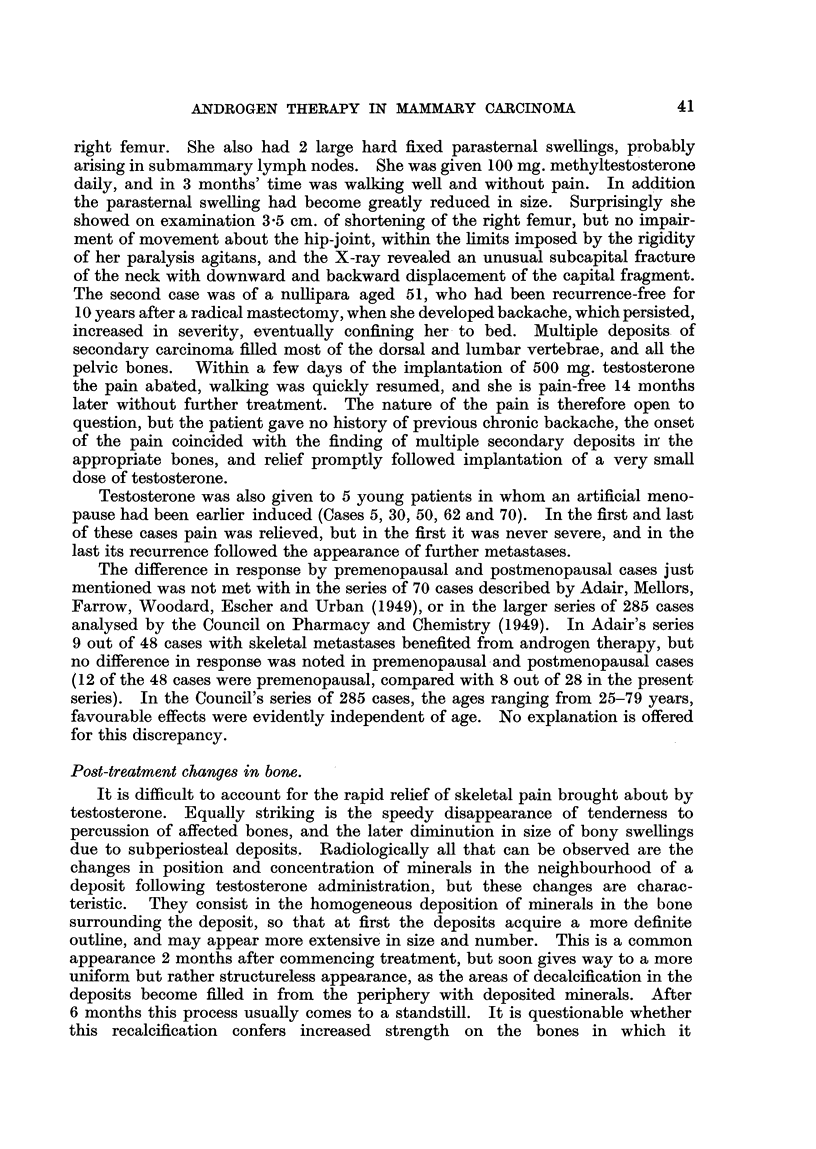

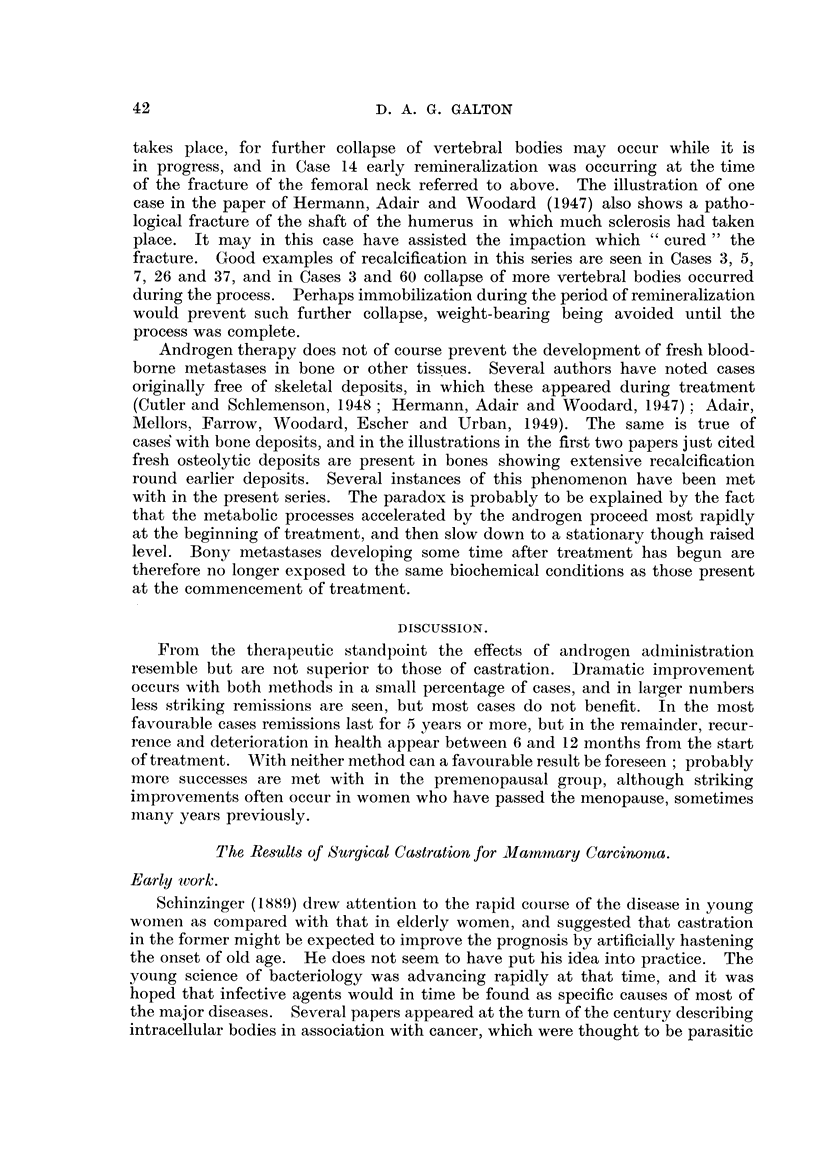

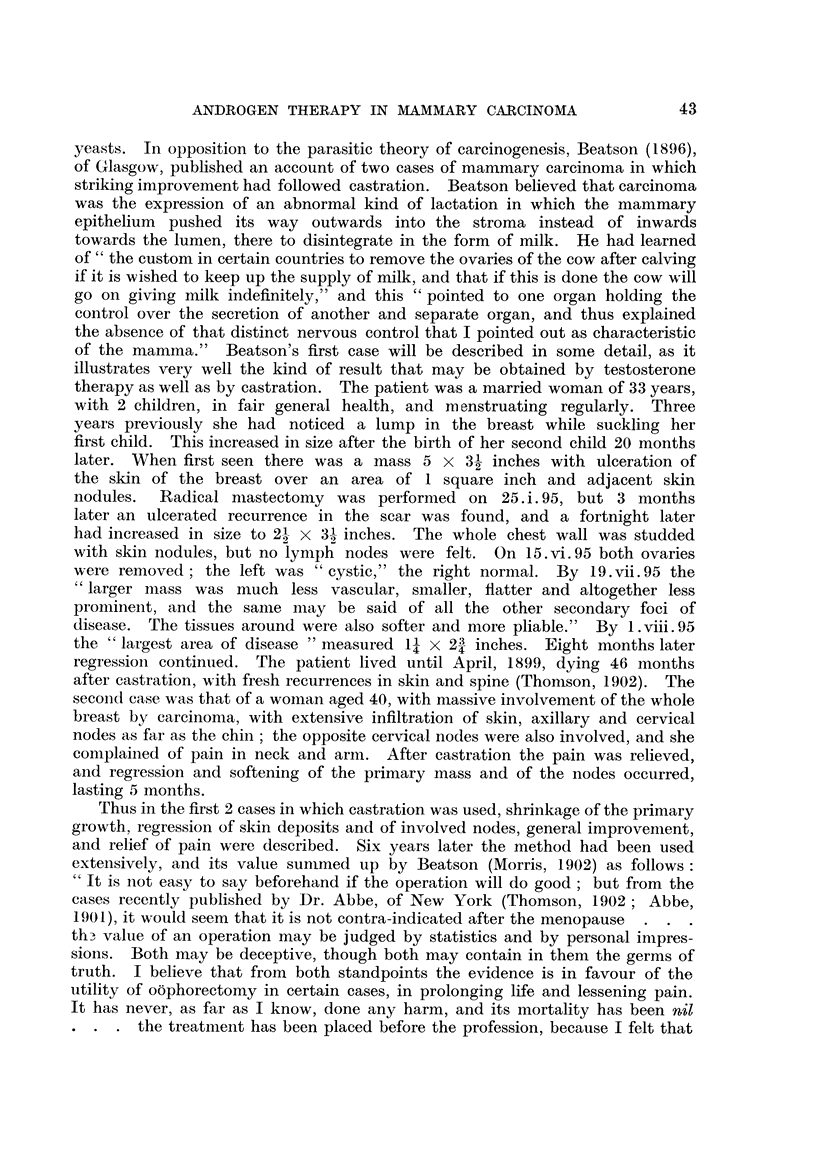

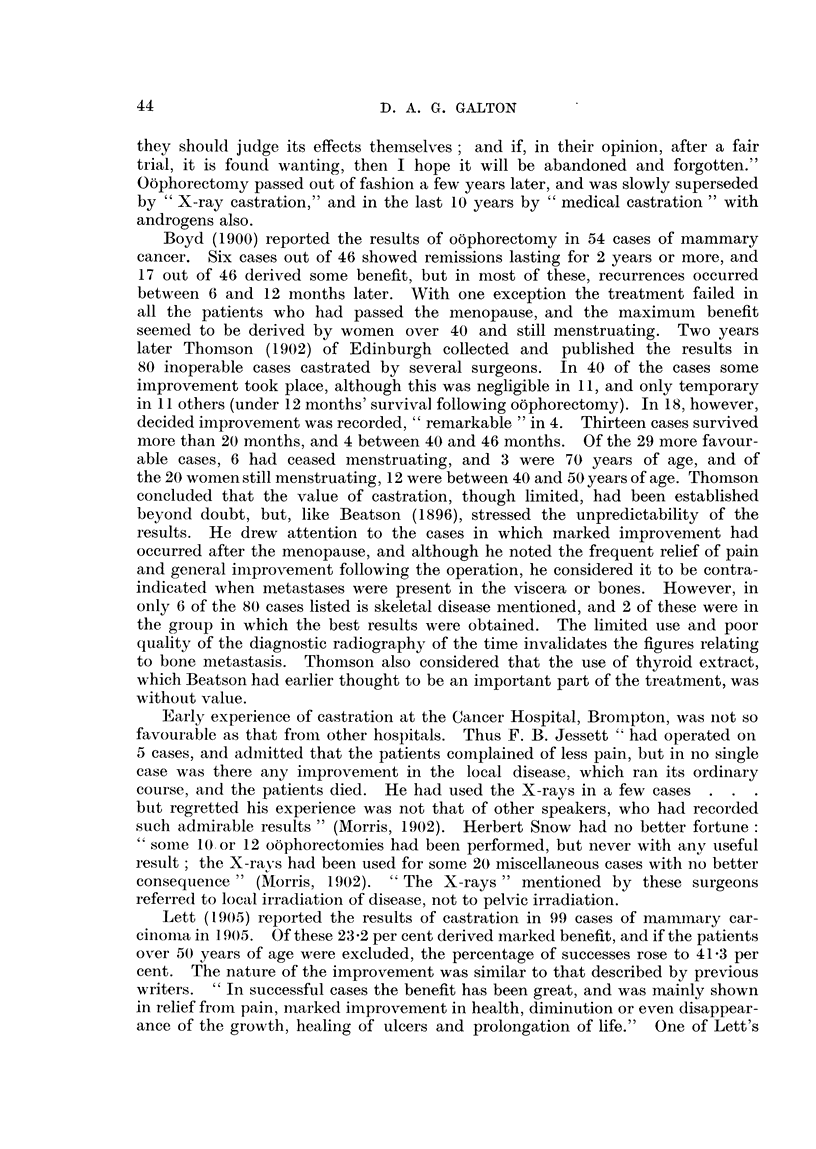

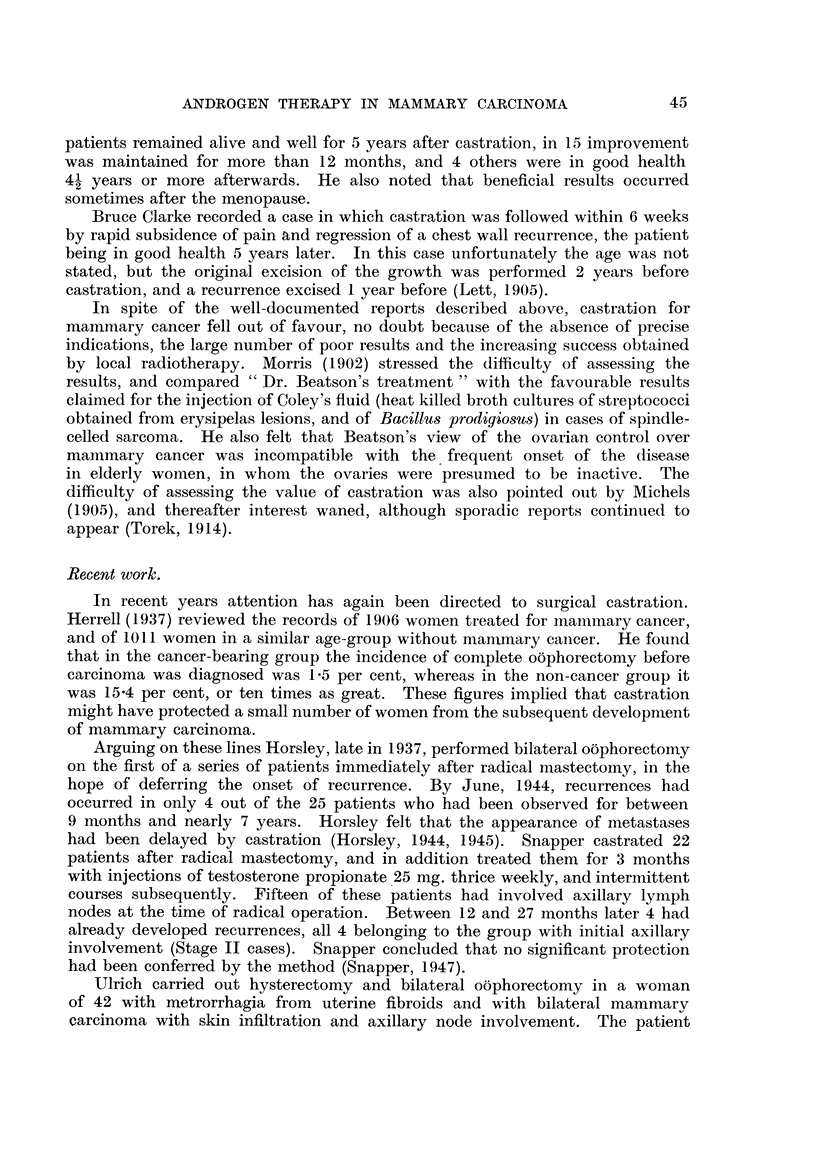

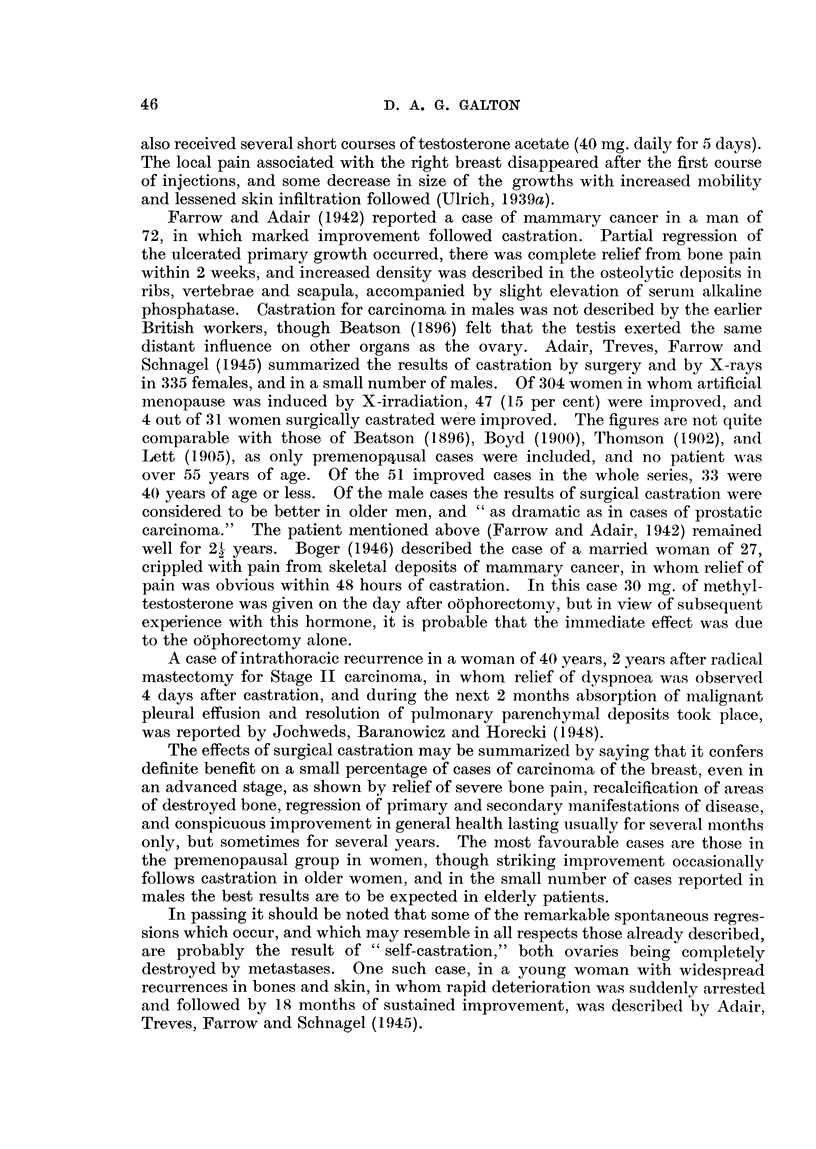

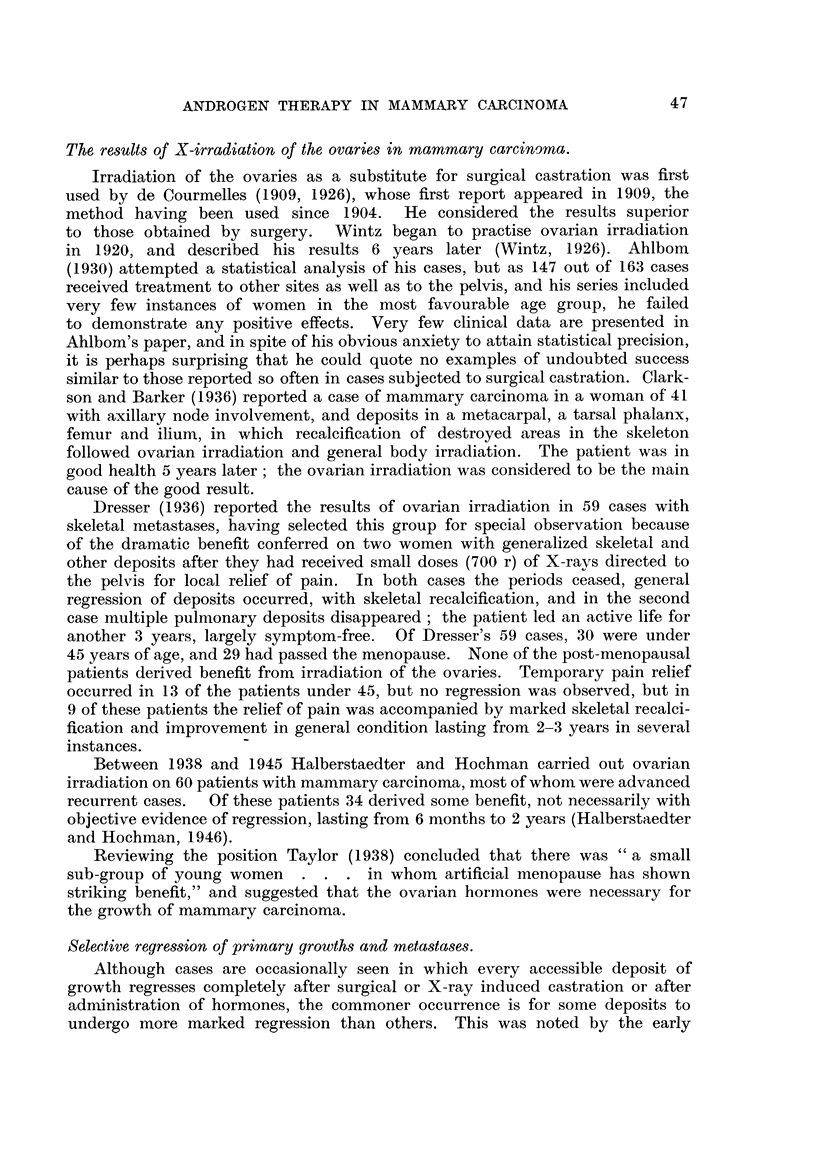

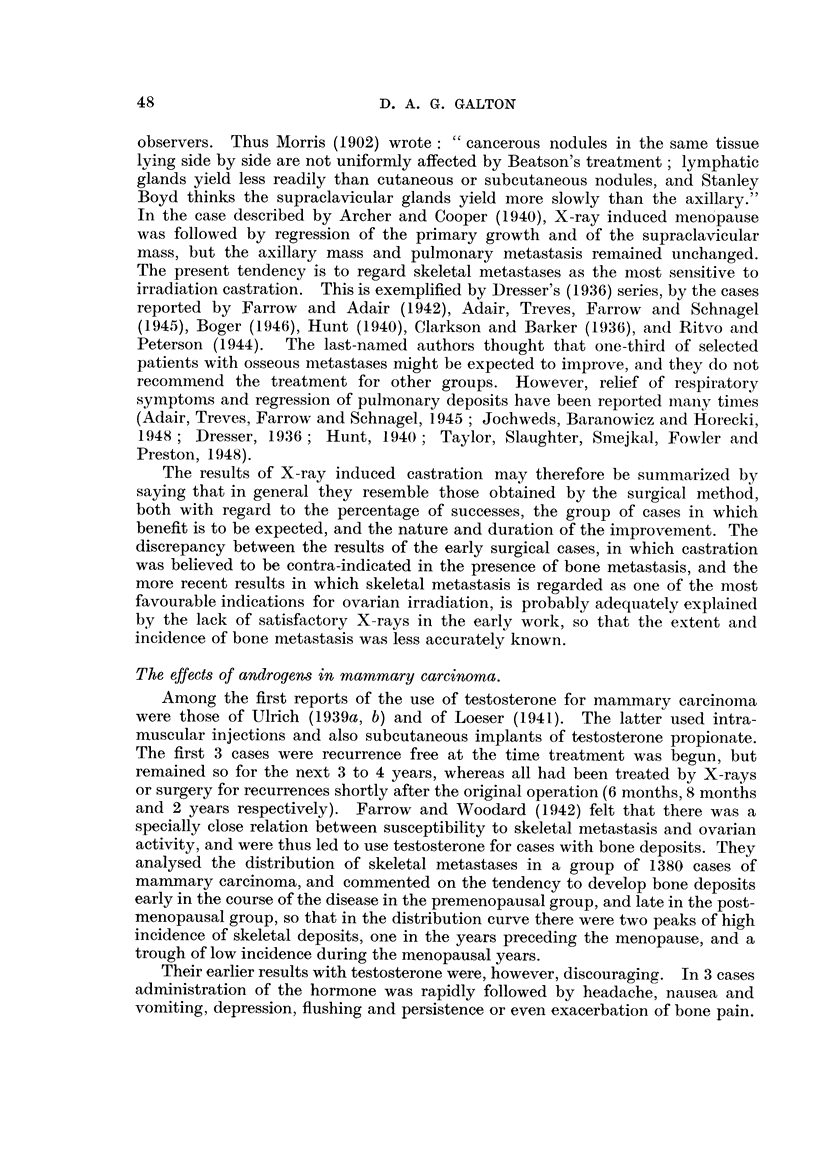

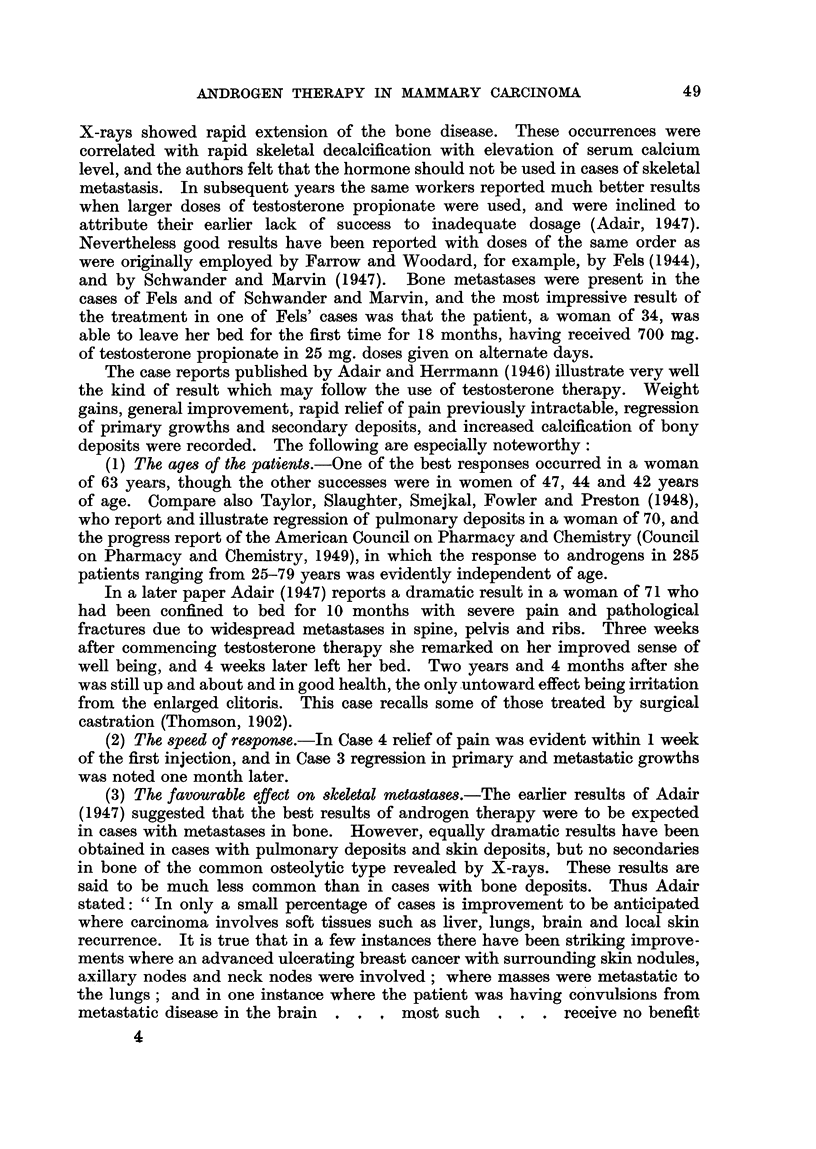

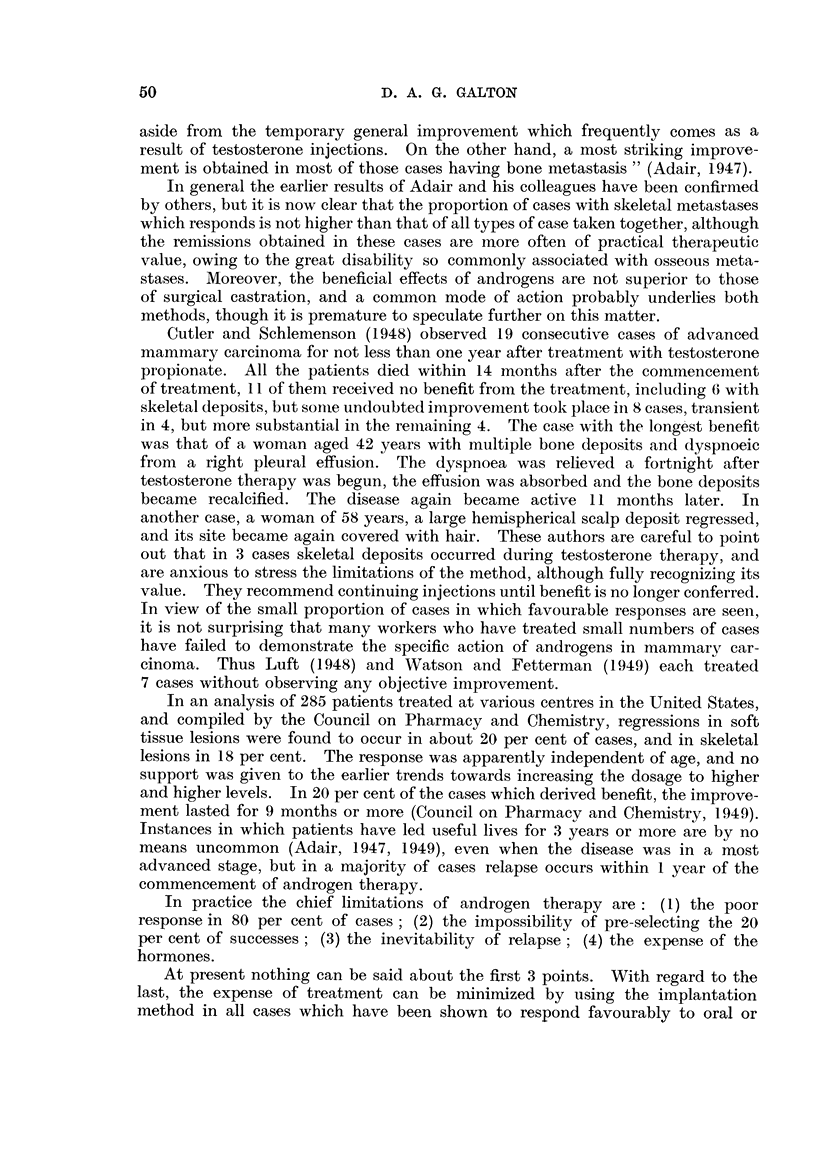

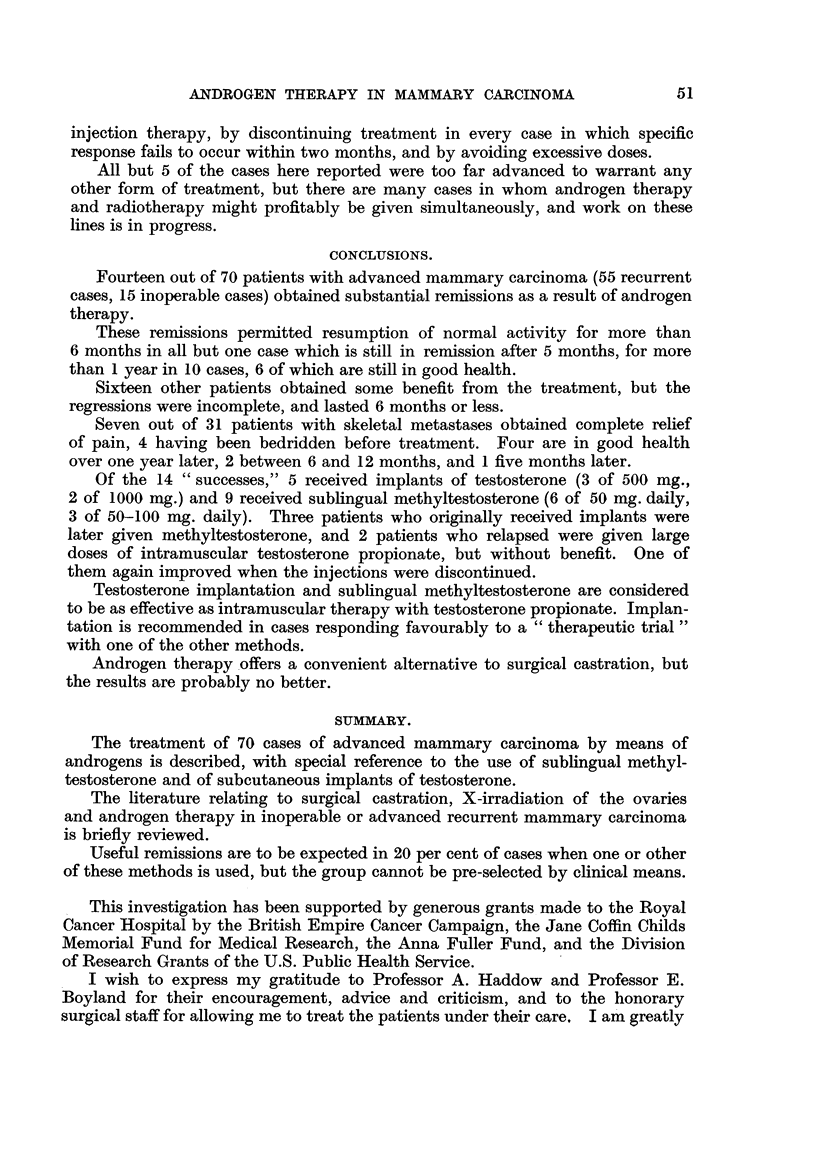

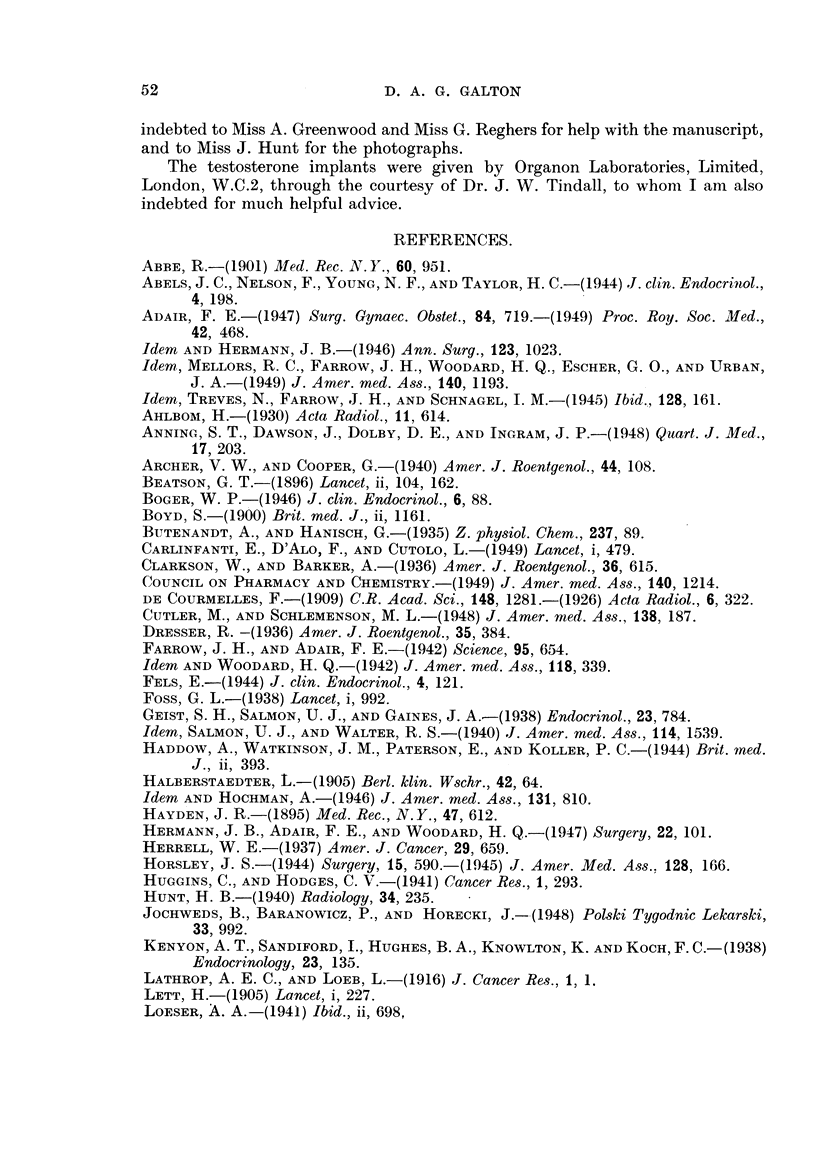

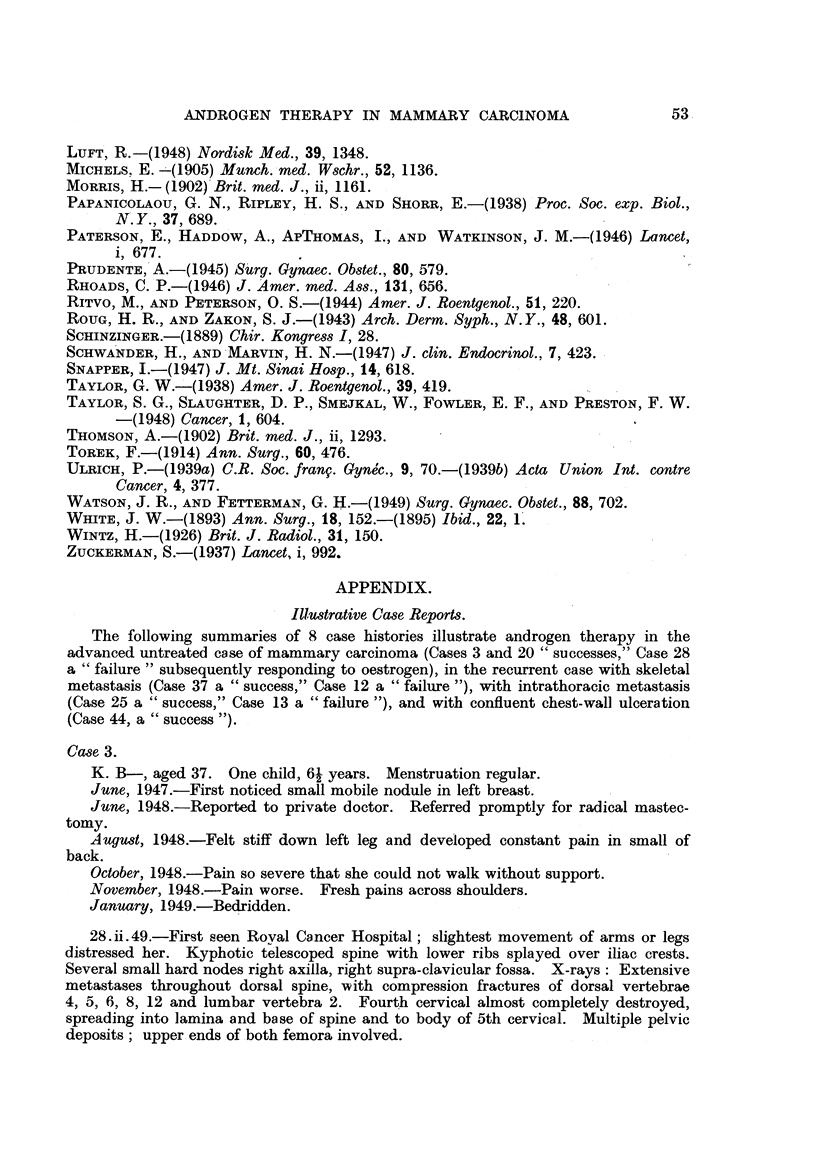

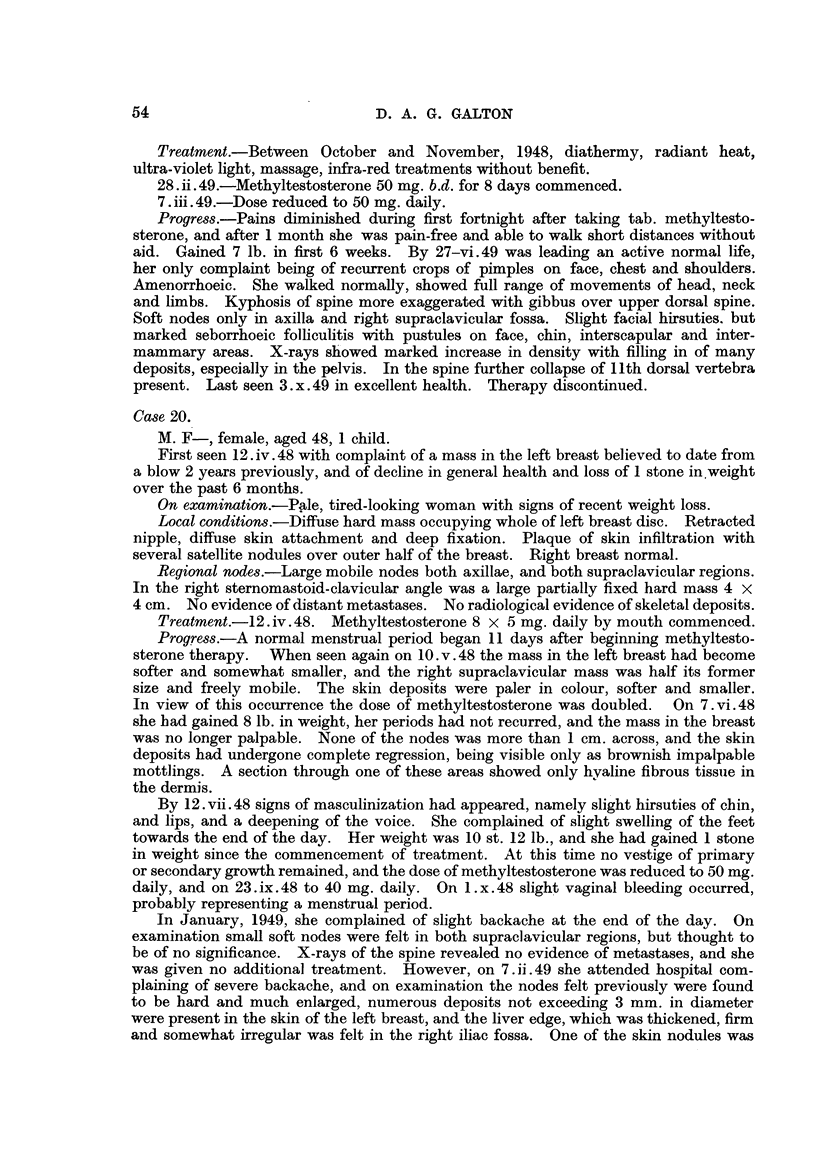

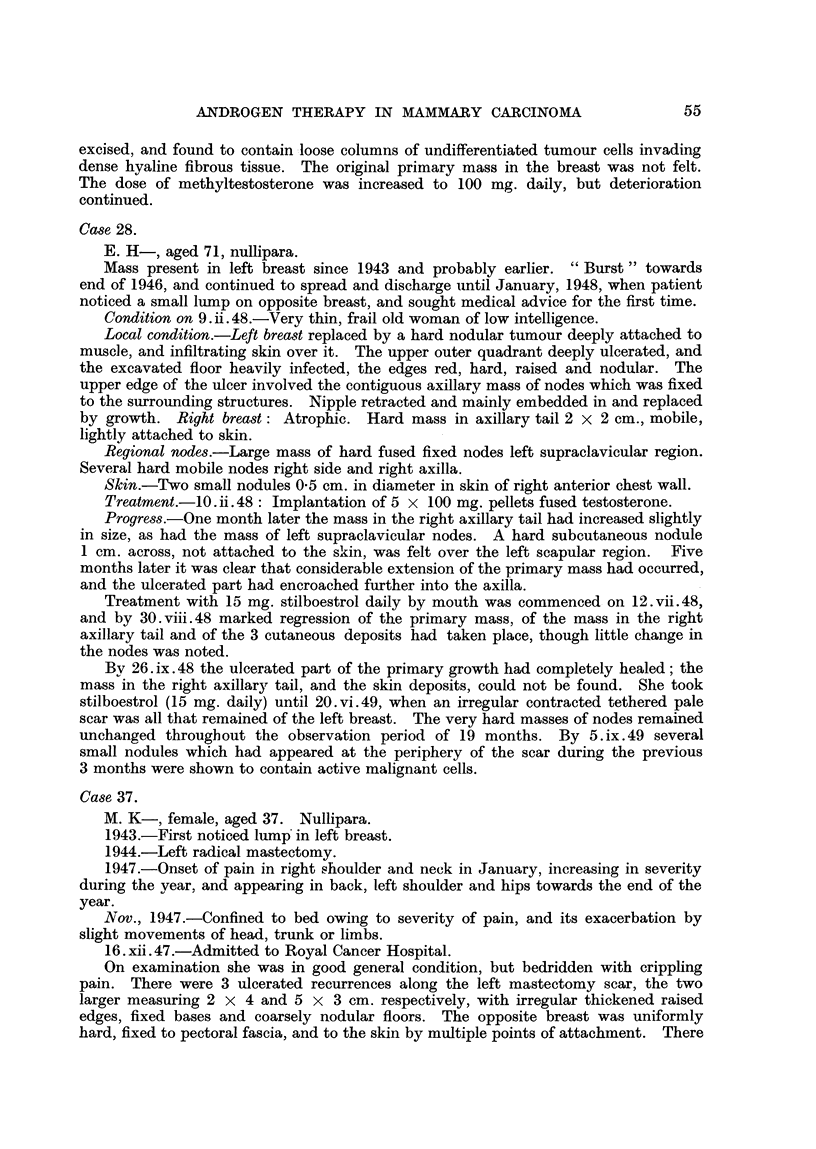

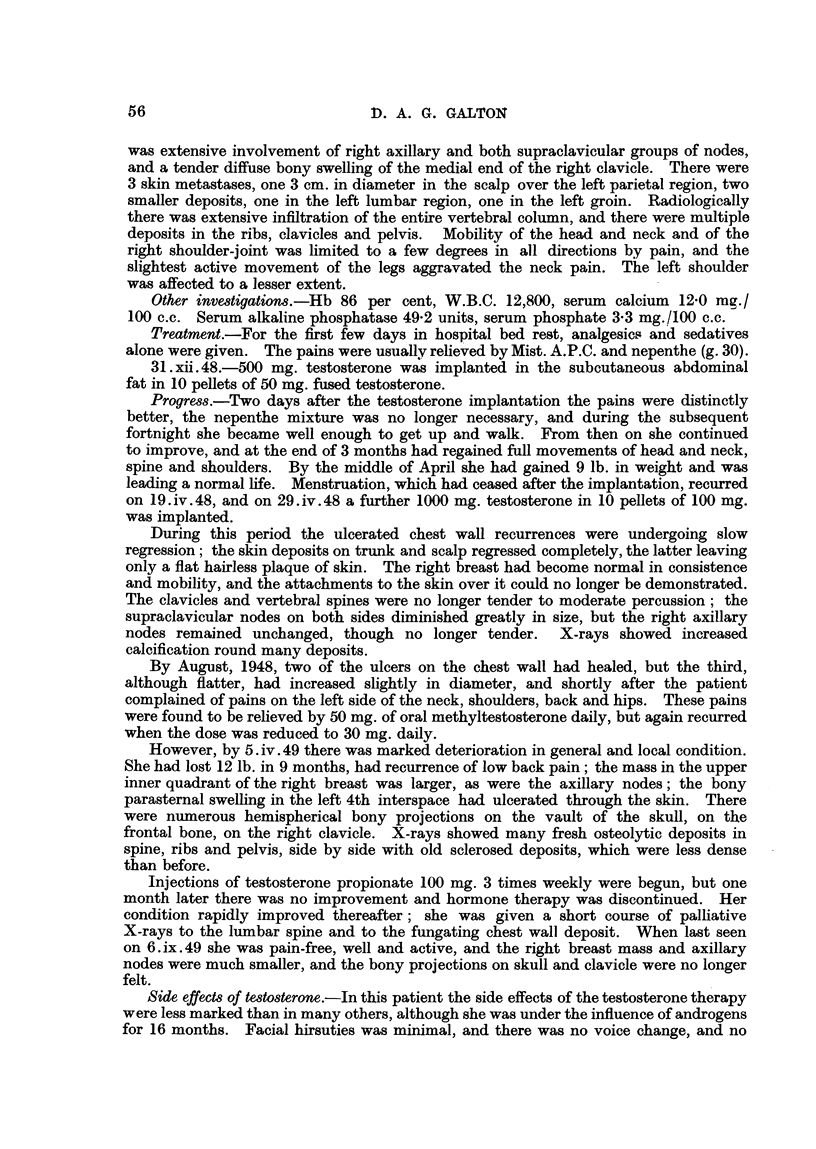

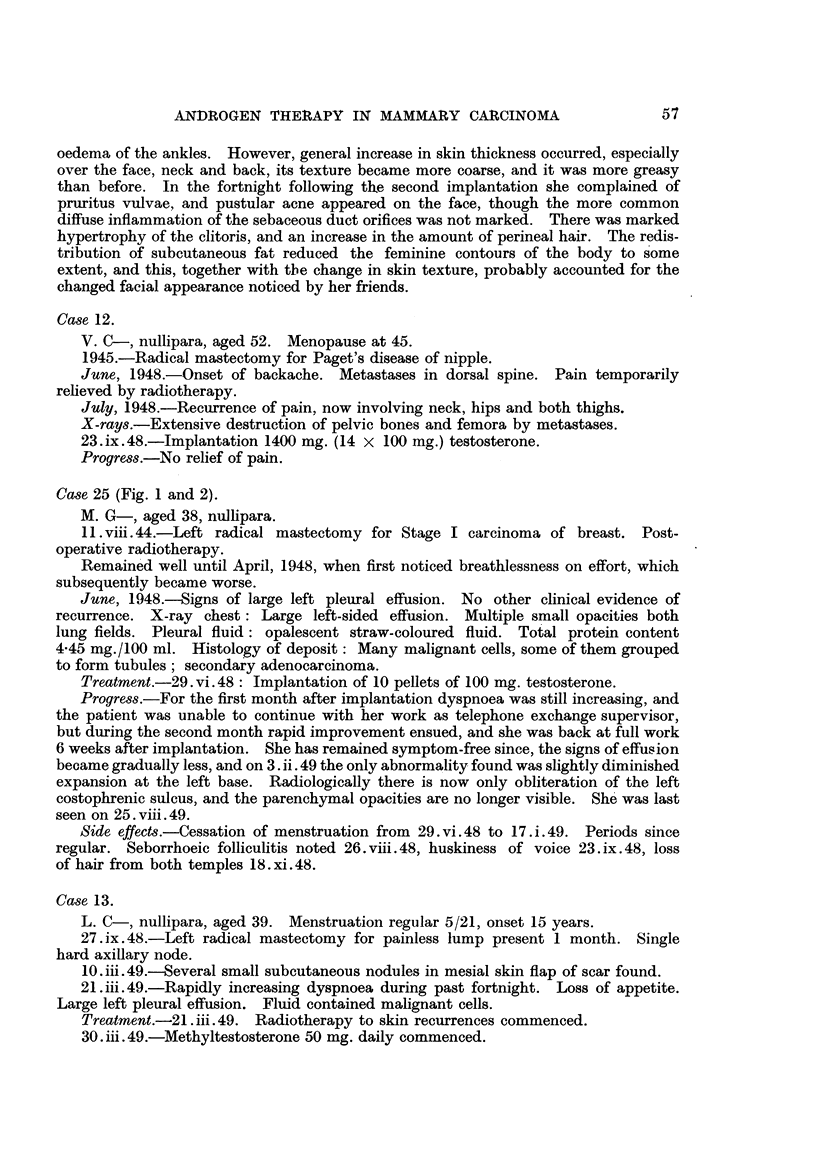

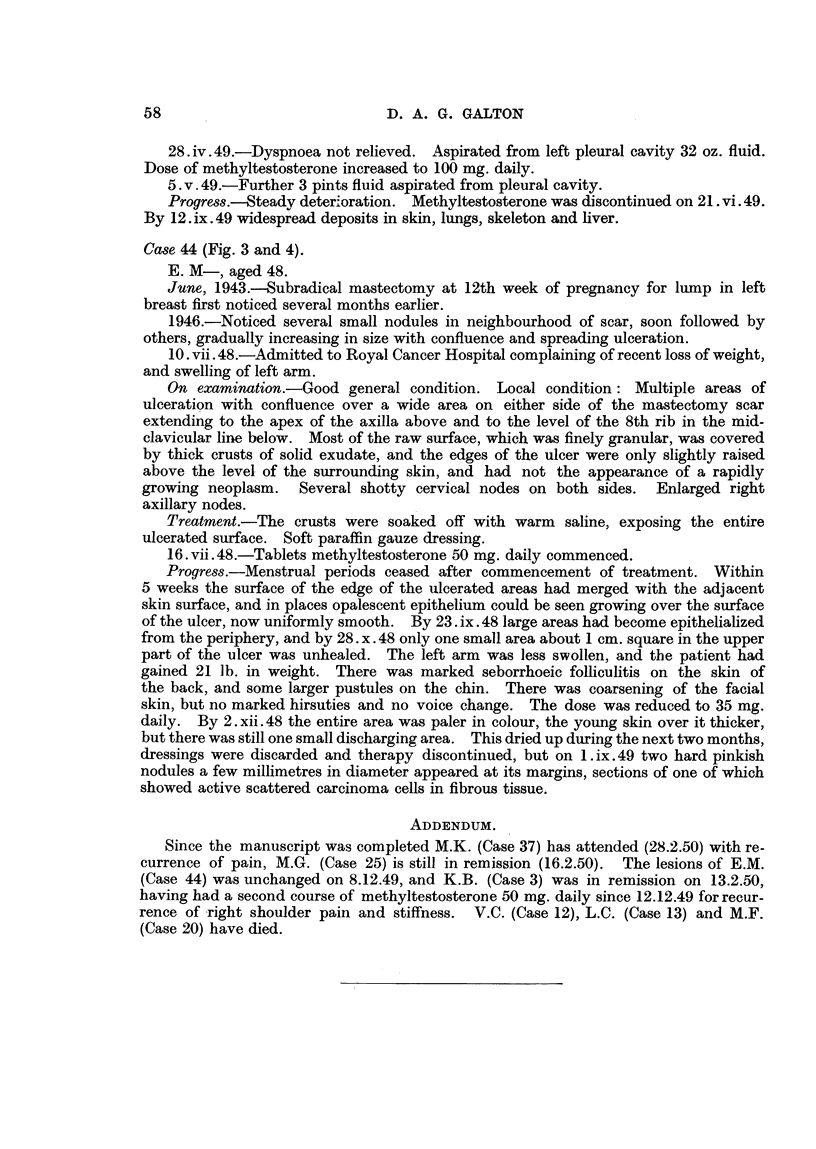


## References

[OCR_02096] Farrow J. H., Adair F. E. (1942). EFFECT OF ORCHIDECTOMY ON SKELETAL METASTASES FROM CANCER OF THE MALE BREAST.. Science.

[OCR_02170] Torek F. (1914). DISAPPEARANCE OF RECURRENT MAMMARY CARCINOMA AFTER REMOVAL OF THE OVARIES.. Ann Surg.

[OCR_02175] White J. W. (1893). II. The Present Position of the Surgery of the Hypertrophied Prostate.. Ann Surg.

